# Supplementary material for: Versatile Platinum(IV) Prodrugs of Naproxen and Acemetacin as Chemo-Anti-Inflammatory Agents
Source: Cancers (Basel). 2023 Apr 26;15(9):2460. doi: 10.3390/cancers15092460 (PMC10177380; doi:10.3390/cancers15092460)
Supplement: Supplementary file 1 [file cancers-15-02460-s001.zip › cancers-2280953-supplementary.pdf]

SUPPLEMENTARY MATERIALS

Versatile Platinum(IV) Prodrugs of Naproxen and Acemetacin as Chemo-Anti-Inflammatory Agents

Angelico D. Aputen [1], Maria George Elias [1,2], Jayne Gilbert [3], Jennette A. Sakoff [3], Christopher P. Gordon [1], Kieran F. Scott [2,4] and Janice R. Aldrich-Wright [1,*]

[1] School of Science, Western Sydney University, Locked Bag 1797, Penrith South, Sydney, NSW 2751, Australia
[2] Ingham Institute, Liverpool, Sydney, NSW 2170, Australia
[3] Calvary Mater Newcastle Hospital, Waratah, Newcastle, NSW 2298, Australia
[4] School of Medicine, Western Sydney University, Locked Bag 1797, Penrith South, Sydney, NSW 2751, Australia
* Correspondence: j.aldrich-wright@westernsydney.edu.au; Tel.: +61-246203218

SYNTHESIS, INSTRUMENTATION AND MEASUREMENTS

SYNTHESIS

This section summarises all procedures undertaken in synthesising the NHS esters of NPX and ACE, and the studied complexes, **1–6**.

1. Synthesis of NHS esters

Each acid was reacted with 1 mol eq. of NHS and DCC in C_3H_6O. Each reaction solution was left stirring at room temperature for 24 h. The precipitated by-product, dicyclohexylurea (DCU), was removed through syringe filtration. The filtrate was collected and reduced to dryness through rotary evaporation. All NHS esters were used without further purification.

2. Synthesis of platinum(IV) complexes, **1–6**

The appropriate precursor, **$[Pt^{IV}(H_L)(A_L)(OH)_2]^{2+}$**, was reacted with 2 mol eq. of NHS esters of NPX or ACE in DMSO (1 – 2 mL) for 72 h at room temperature, in the dark. The reaction solution was washed with excess Et_2O and vigorously mixed using a plastic pipette, followed by centrifugation to afford a colourless supernatant and an oily brown layer. The colourless supernatant was discarded while the oily brown layer was collected and dissolved in methanol (1 – 2 mL), followed by the addition of excess Et_2O to induce precipitation. Centrifugation was undertaken to collect the final precipitate. Excess C_3H_6O was mixed with the precipitate and sonicated, affording a pure and solidified yellow or green precipitate that was collected through centrifugation.

INSTRUMENTATION

This section summarises all procedures undertaken in confirming the structure and purity of the studied complexes, **1**–**6**.

1. Flash Chromatography

A Biotage Isolera™ One flash chromatography system (Shimadzu, Sydney, NSW, Australia) equipped with a Biotage® Sfär C18 D (Duo 100 Å 30 µm 30 g) (Shimadzu, Sydney, NSW, Australia) was employed to purify the platinum(IV) complexes. The mobile phase consisted of solvents, A (d.i.H_2O) and B (MeOH). The samples were dissolved in d.i.H_2O/MeOH (50:50) and eluted through the column with a 0 – 40% linear gradient for 50 min with a flow rate of 4 mL.min^{-1}, collected within the set wavelengths of 200 – 400 nm.

2. High-Performance Liquid Chromatography (HPLC)

An Agilent (Melbourne, VIC, Australia) Technologies 1260 Infinity instrument equipped with a Phenomenex Onyx™ Monolithic C_{18}-reverse phase column (100 × 4.6 mm, 5 µm pore size) (Sydney, NSW, Australia) was utilised for the complexes. The mobile phase consisted of solvents, A (0.06% TFA in d.i.H_2O) and B (0.06% TFA in CH_3CN/d.i.H_2O (90:10)). An injection volume of 5 µL was utilised and eluted with a 0 – 100% linear gradient over 15 min with a flow rate of 1 mL.min^{-1}, at the set wavelengths of 214 and 254 nm. An Agilent ZORBAX RX-C_{18} column (100 × 4.6 mm, 3.5 µm pore size) (Sydney, NSW, Australia) was utilised for the NHS esters of NPX and ACE using the same method described above.

3. Nuclear Magnetic Resonance (NMR) Spectroscopy

^{1}H-NMR, 2D-COSY, ^{1}H-^{195}Pt-HMQC and 1D-^{195}Pt-NMR were carried out on a 400 MHz Bruker (Melbourne, VIC, Australia) Avance spectrometer at 298 K. All complexes were prepared to a concentration of 10 mM in 600 μL using D_2O. DMSO-d_6 was utilised for the NHS esters of NPX and ACE. ^{1}H-NMR was set to 10 ppm and 16 scans with a spectral width of 8250 Hz and 65,536 data points. 2D-COSY was acquired using a spectral width of 3443 Hz for both ^{1}H nucleus, F1 and F2 dimensions, with 256 and 2048 data points, respectively. ^{1}H-^{195}Pt-HMQC was carried out using a spectral width of 214,436 Hz and 256 data points for ^{195}Pt nucleus, F1 dimension, also a spectral width of 4808 Hz with 2048 data points for ^{1}H nucleus, F2 dimension. 1D-^{195}Pt was measured using a spectral width of 85,470 Hz and 674 data points. All resonance recorded, were presented as chemical shifts in parts per million (δ ppm) with *J*-coupling constants reported in Hz. For spin multiplicity: s (singlet); d (doublet); dd (doublet of doublets); t (triplet); q (quartet) and m (multiplet). All spectroscopic data gathered were generated and plotted using TopSpin 4.1.3 analysis software.

4. Ultraviolet-visible (UV) Spectroscopy

An Agilent (Melbourne, VIC, Australia) Technologies Cary 3500 UV-Vis Multicell Peltier spectrophotometer was utilised to perform the UV spectroscopy experiments. UV spectroscopic experiments were completed at room temperature in the range of 200 – 400 nm with a 1 cm quartz cuvette. All complexes were prepared in d.i.H_2O, while the NHS esters of NPX and ACE were prepared in CH_3CN. A stock solution of each complex or compound (1 mM) was prepared, and absorption spectra were recorded at a series of different concentrations by titrating 9 × 3 μL aliquots into a cuvette containing d.i.H_2O (3000 μL). Experiments were repeated in triplicate. All spectra were baseline corrected by the instrument – a baseline containing d.i.H_2O was acquired first, and automatically subtracted from each experiment. Average extinction coefficients (ε) were determined with standard deviation and errors based on the generated plot curves.

5. Circular Dichroism (CD) Spectroscopy

A Jasco (Easton, PA, United States) J-810 CD spectropolarimeter was used to measure the CD spectra of the complexes. The samples were prepared in d.i.H_2O in a 1 mm optical glass cuvette or 1 cm quartz cuvette. CD experiments were undertaken at room temperature in the wavelength range of 200 – 400 nm (30 accumulations) with a bandwidth of 1 nm, data pitch of 0.5 nm, a response time of 1 sec and a 100 nm.min^{-1} scan speed. The flowrate of nitrogen gas was 6 L.min^{-1}. The HT (photo-multiplier) level remained below 500 V for all experiments. A CD simulation tool (CDToolX) was used to generate the spectra.

6. Electrospray Ionisation Mass Spectrometry (ESI-MS)

High-resolution ESI-MS experiments were undertaken using a Waters (Sydney, NSW, Australia) SYNAPT G2-Si quadruple time-of-flight (QTOF) HDMS. A stock solution of each complex (1 mM) was prepared in d.i.H_2O. 5 μL of the stock solution was diluted with 995 μL of d.i.H_2O to create the sample solution. For the NHS esters of NPX and ACE, CH_3CN was used to prepare the stock solutions (1 mM). 5 μL of the stock solution for each NHS ester was also diluted with 995 μL of CH_3CN to create the sample solutions. The wire or capillary where the sample solutions were injected was washed with d.i.H_2O/CH_3CN (50:50) before every experiment to avoid cross-contamination.

7. Infrared Spectroscopy (IR)

A Nicolet™ iS™ 5 FTIR Spectrometer (Sydney, NSW, Australia) was utilised to measure the IR spectra of the complexes. IR experiments were undertaken at room temperature, within the wavelength range of 400 – 4000 cm^{-1}, and the number of scans was 16. The resolution was set to 4 cm^{-1}. The optical velocity was 0.4747 m/s. For every sample run, a

background spectrum was collected. OMNIC™ Series Software was used to process the spectra.

MEASUREMENTS

This section summarises all procedures undertaken to determine the physicochemical and biological properties of the studied complexes, **1**–**6**.

1. Lipophilicity Studies

Elution profiles were acquired on an Agilent (Melbourne, VIC, Australia) Technologies 1260 Infinity instrument equipped with a Phenomenex Onyx™ Monolithic C_{18}-reverse phase column (100 × 4.6 mm, 5 µm pore size) (Sydney, NSW, Australia). The mobile phase consisted of solvents, A (0.06% TFA in d.i.H_2O) and B (0.06% TFA in CH_3CN/d.i.H_2O (90:10)). Potassium iodide was used as an external dead volume marker to determine the dead time of the column. The retention time (T_R) of the complexes were measured at varying isocratic ratios ranging from 32 to 50% of solvent B at 1 mL.min^{-1}. An injection volume of 10 µL was utilised. Capacity factors were determined according to Equation (1):

$$k = (T_R - T_0)/T_0 \qquad (1)$$

where k is the capacity factor, T_R is the retention time of the analyte, and T_0 represents the dead time. A minimum of four different mobile compositions were used for each complex to calculate k. A linear plot was generated of log k against the concentration of CH_3CN in the mobile phase to determine the value of log k_w expressed by Equation (2):

$$\log k = S\varphi + \log k_w \qquad (2)$$

where S is the slope, φ is the concentration of the CH_3CN in the mobile phase, and log k_w represents the capacity factor of the complex in 100% d.i.H_2O. Extrapolation of this linear plot to the *y*-intercept indicates the log k_w value.

2. Reduction Studies

A sequence of ^{1}H-NMR experiments was carried out for 1 h at 37 °C, followed by 1D-^{195}Pt-NMR within the regions of −2800 and 400 ppm. An amount of 10 mM PBS (~7.4 pH) was transferred to a vial and reduced to dryness through rotary evaporation. AsA (~1 mg) was combined with the metal complex (~5 mg) and transferred to the vial containing the dried PBS. 600 μL of D_2O was then added to the vial to dissolve the complex, AsA and the PBS together. Each reaction was followed at 37 °C until the complete reduction of the complexes.

3. Cell Viability Assays

The cell lines tested were: HT29 colon, U87 glioblastoma, MCF-7 breast, A2780 ovarian, H460 lung, A431 skin, Du145 prostate, BE2-C neuroblastoma, SJ-G2 glioblastoma, MIA pancreas, ADDP ovarian variant, and the non-tumour derived MCF10A breast line. In addition to **1–6**, NPX and ACE were also tested for reference and comparison. All test agents were prepared in DMSO (30 mM stock solutions) and stored at −20 °C until use. All cell lines were cultured in a humidified atmosphere 5% carbon dioxide at 37 °C. The cancer cell lines were maintained in Dulbecco's modified Eagle's medium (DMEM) (Trace Biosciences, Sydney, NSW, Australia) supplemented with 10% foetal bovine serum, 10 mM sodium bicarbonate penicillin (100 IU mL^{-1}), streptomycin (100 μg mL^{-1}) and glutamine (4 mM). The non-cancer MCF10A cell line was cultured in DMEM:F12 (1:1) cell culture media, 5% heat inactivated horse serum, supplemented with penicillin (50 IU mL^{-1}), streptomycin (50 μg mL^{-1}), 20 mM 4-(2-hydroxyethyl)-1-piperazineethanesulfonic acid (HEPES), L-glutamine (2 mM), epidermal growth factor (20 ng mL^{-1}), hydrocortisone (500 ng mL^{-1}), cholera toxin (100 ng mL^{-1}) and insulin (10 μg mL^{-1}). Cytotoxicity was determined by plating cells in duplicate in 100 mL medium at a density of 2500 – 4000 cells per well in 96 well plates. On day 0 (24 h after plating) when the cells were in logarithmic growth, 100 μL of medium with or without the test agent was added to each well. After 72 h, the GI_{50} was evaluated using the MTT (3-[4,5-

dimethylthiazol-2-yl]-2,5-diphenyltetrazolium bromide) assay and absorbance was read at 540 nm. An eight-point dose response curve was produced from which the GI_{50} value was calculated, representing the drug concentration at which cell growth was inhibited by 50% based on the difference between the optical density values on day 0 and those at the end of drug exposure.

4. Reactive Oxygen Species (ROS) Potential

A total of 25,000 cells/mL of HT29 cells in DMEM were seeded in 96-well plates. Cells were washed with 1X kit buffer, and then stained with 25 μM 2′,7′-dichlorofluorescein diacetate (DCFH-DA) and incubated for 45 min. DCFH-DA was then removed, and cells were then re-washed with 1X kit buffer, after which phenol red free media was added. Cells were then treated with a GI_{50} drug concentration for each complex. The plates were directly scanned to measure fluorescence (relative fluorescence units (RFU)) at different time points using the Glo-Max®-Multimode microplate reader (Promega Corporation, Alexandra, VIC, Australia) at an excitation/emission of 485/535 nm. To generate the positive control (20 μM *tert*-butyl hydroperoxide (TBHP)), cells were washed with 1X kit buffer, and stained with DCFDA (25 μM) for 45 min; this was removed and TBHP was added in phenol red free media, and the resulting solution was scanned as described above.

5. Mitochondrial Membrane Potential (MtMP)

In 96-well plates, 25,000 cells/mL of HT29 colon cells in DMEM were seeded. Cells were treated with a GI_{50} drug concentration for each complex. At 24, 48 or 72 h, cells were washed with PBS and stained with 1 μM tetramethylrhodamine, ethyl ester (TMRE) and incubated for 30 min. TMRE was then removed, and cells were then re-washed with PBS (0.2% BSA), after which phenol red free media was added. The plates were directly scanned to measure fluorescence (expressed as RFU), using the Glo-Max®-Multimode

microplate reader (Promega Corporation, Alexandra, VIC, Australia) at an excitation/emission of 549/575 nm. For the positive control, 20 μM carbonyl cyanide 4-(trifluoromethoxy) phenylhydrazone (FCCP)) was added to the cells and incubated for 10 min. The cells were washed with PBS (0.2% BSA) and stained with TMRE (1 μM) for 30 min; this was removed and phenol red free media was added, and the final solution was scanned as mentioned above.

6. Cyclooxygenase-2 (COX-2) Inhibition

The standards, samples and assay preparation, and completion were achieved as instructed by the manufacturer. The assay necessitated three steps, being the standards preparation, the COX reaction, and the enzyme immunoassay. The standards were prepared in 2-fold serial dilution of the prostaglandin $F_{2\alpha}$ enzyme-linked immunosorbent assay (ELISA) standard starting at 500 pg/mL to 3.9 pg/mL in ELISA buffer (1X). The COX reaction dilution preparation for the background samples included the addition of background COX-2 (20 μL), which was inactivated in boiling water for 3 min. The background values were generated by adding 10 μL of the inactivated COX-2, 160 μL of the reaction buffer (1X), 10 μL of heme and 10 μL of DMSO (inhibitor vehicle). The COX reaction dilution preparation for the COX-2 100% initial activity samples included the addition 160 μL of the reaction buffer (1X), 10 μL of heme, 10 μL of COX-2 and 10 μL of DMSO (inhibitor vehicle). The COX reaction dilution preparation for the COX-2 inhibitor samples included the addition of 160 μL of the reaction buffer (1X), 10 μL of heme, 10 μL of COX-2 and 10 μL of the sample being investigated for COX-2 inhibition (**1**–**6**, NPX, ACE, the precursor platinum(II) scaffolds (**PHEN*SS***, **5ME*SS*** and **56ME*SS***), and cisplatin). All reactions were incubated at 37 °C for 10 min. The reaction was then initiated by the addition of 10 μL of arachidonic acid and potassium hydroxide solution (1:1), and then incubated at 37 °C for 30 sec prior to the addition of 30 μL of tin(II) chloride to halt enzyme catalysis. The tubes were then vortexed and incubated for 5 min at room

temperature. The background samples were diluted with ELISA buffer (1X) 1:100 times and the COX-2 100% initial activity and COX-2 inhibitor samples were diluted 1:2000 and 1:4000 times. For the enzyme immunoassay, 100 µL of the ELISA buffer (1X) was added to non-specific binding wells (NSBs), 50 µL of the ELISA buffer (1X) was added to maximum binding wells (B_0) and 50 µL of each prostaglandin $F_{2\alpha}$ standard was added to standard wells in duplicates. 50 µL of background sample and 50 µL of COX-2 100% initial activity samples were independently added to the wells in duplicates. Inhibitor samples (50 µL) being investigated were added to the wells in triplicates. Prostaglandin $F_{2\alpha}$ acetyl-cholinesterase (AChE) tracer (50 µL) was added to all wells but the total activity well and blank wells. Prostaglandin $F_{2\alpha}$ ELISA antiserum (50 µL) was added to each well but the total activity well, NSBs and blank wells. The plate was then covered with a plastic seal and incubated overnight at room temperature on an orbital shaker. The wells were then washed five times using kit wash buffer (1X). Ellman's reagent (200 µL) was then added to each well, as well as 5 µL of the AChE tracer into the total activity well. The plate was then covered with the plastic seal, placed on an orbital shaker in the dark to develop for 2 hours at room temperature. The absorbance was then read at 405 nm using the SpectraMax M2 series multi-mode microplate reader (Molecular Devices, San Jose, CA, USA).

CHARACTERISATION DATA

HPLC Chromatograms of NHS Esters

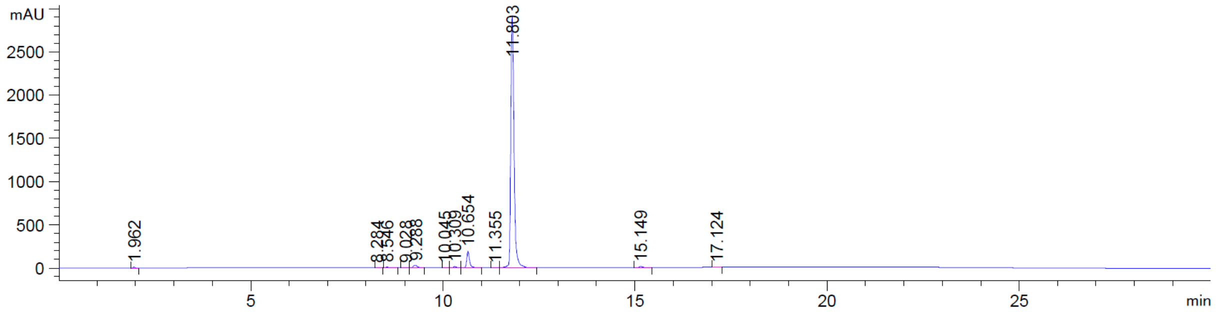

Figure S1: HPLC chromatogram of NHS-NPX with product peak trace at 11.8 min.

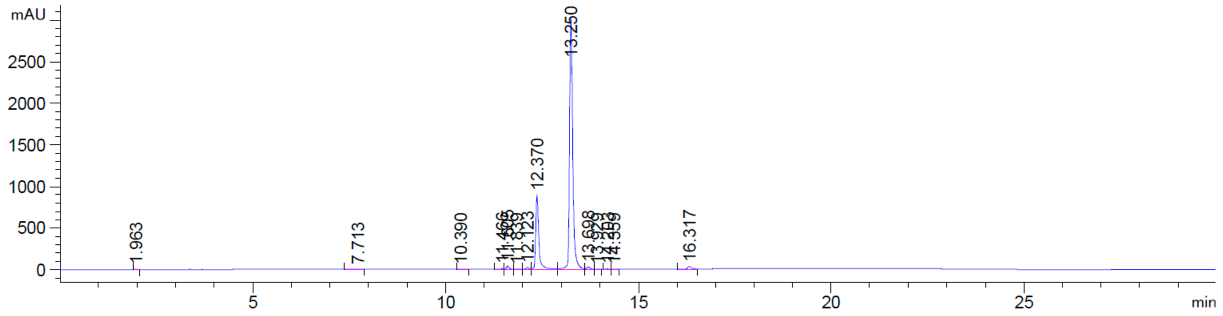

Figure S2: HPLC chromatogram of NHS-ACE with product peak trace at 13.3 min.

^{1}H-NMR Spectra of NHS Esters

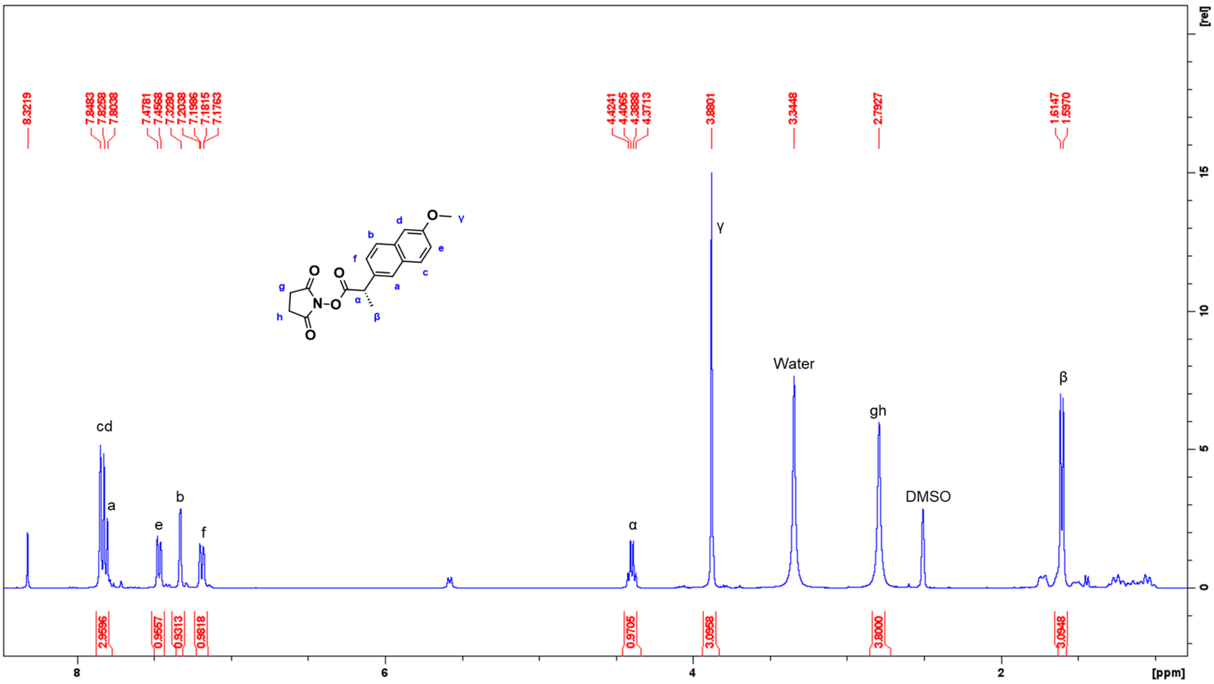

Figure S3: ^{1}H-NMR spectrum of NHS-NPX in DMSO-d$_6$.

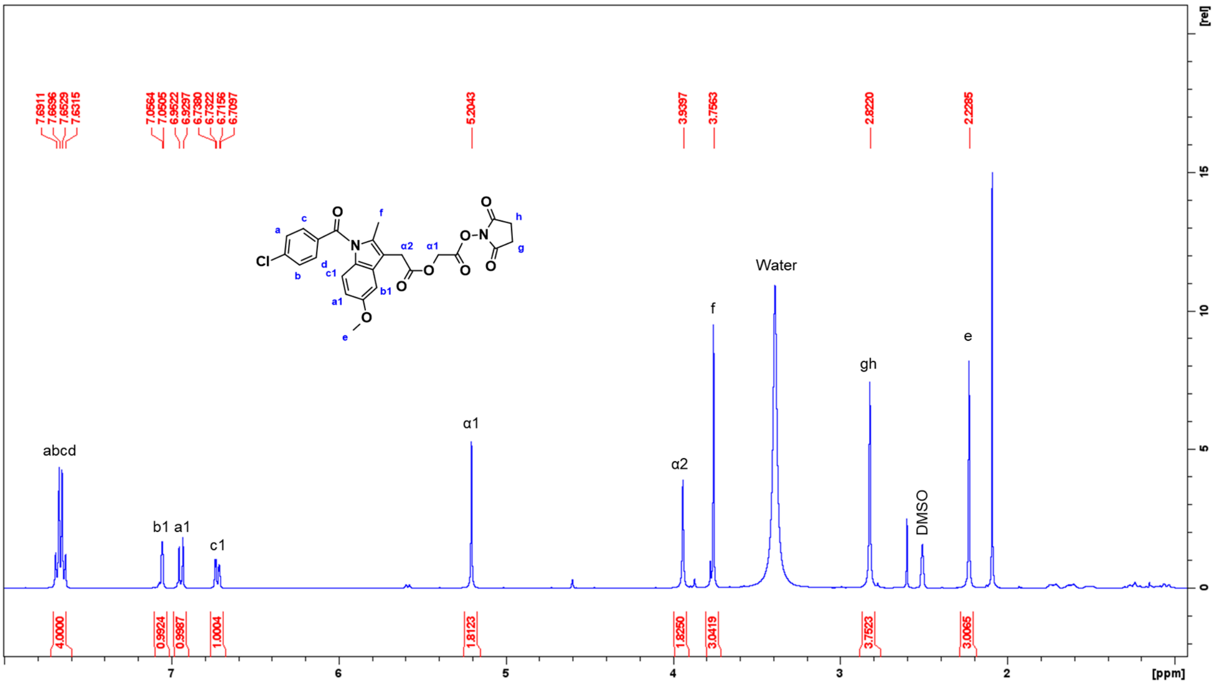

Figure S4: ^{1}H-NMR spectrum of NHS-ACE in DMSO-d$_6$.

ESI-MS Spectra of NHS Esters

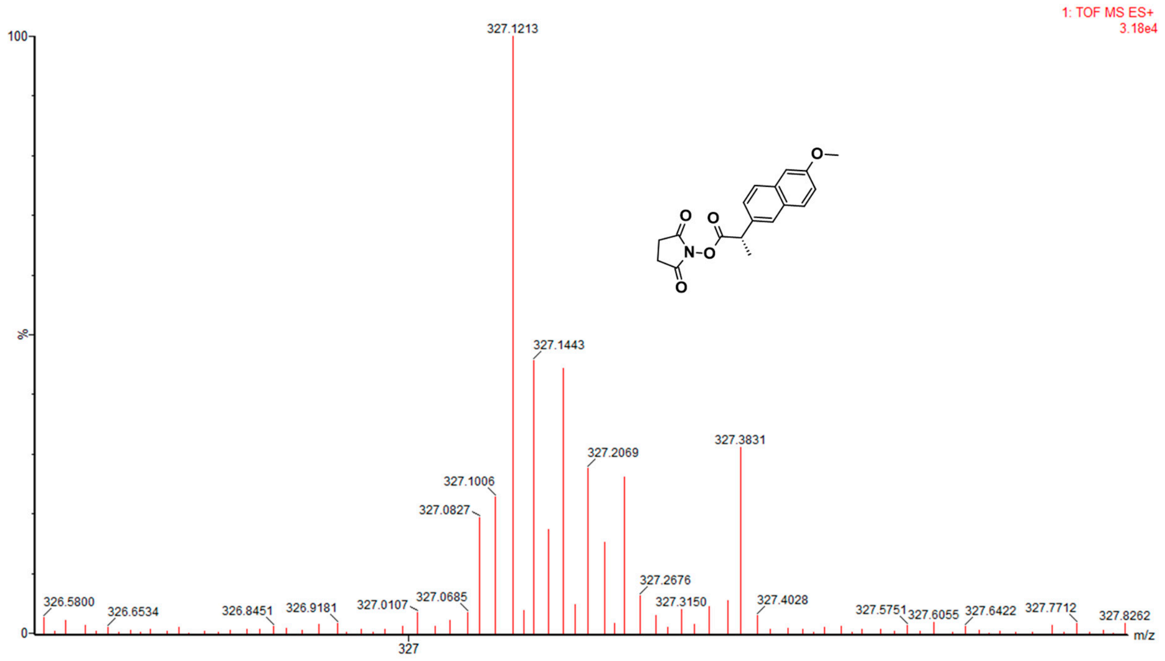

Figure S5: ESI-MS spectrum of NHS-NPX in CH_3CN.

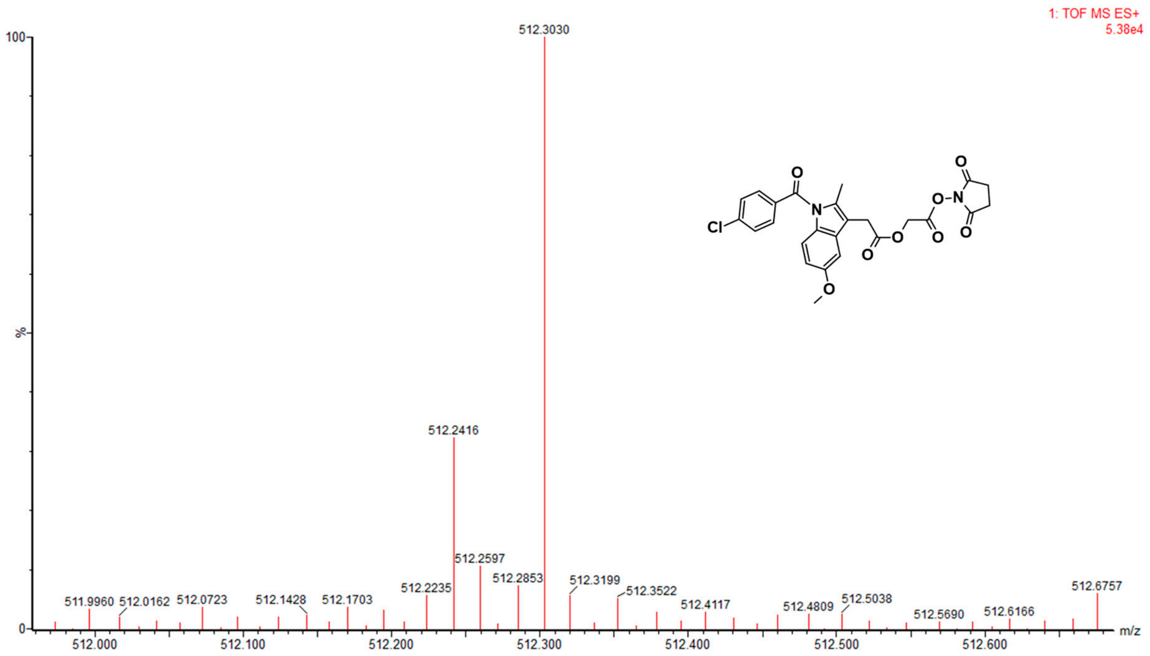

Figure S6: ESI-MS spectrum of NHS-ACE in CH_3CN.

HPLC Chromatograms of 1–6

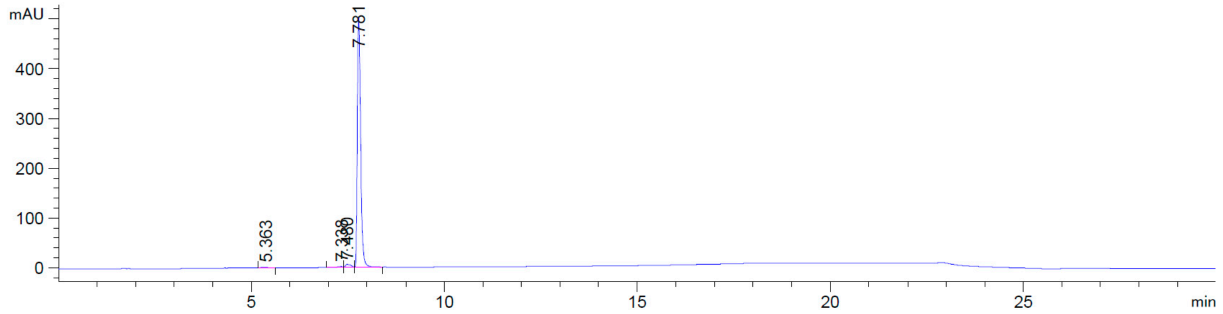

Figure S7: HPLC chromatogram of **1**.

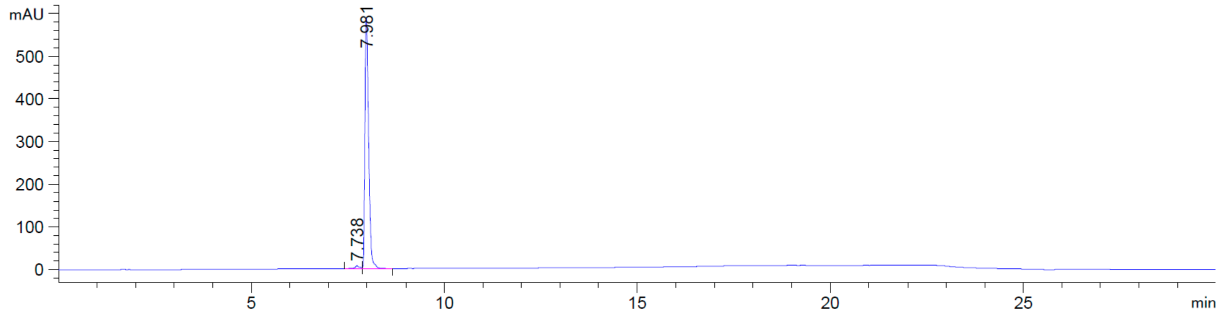

Figure S8: HPLC chromatogram of **2**.

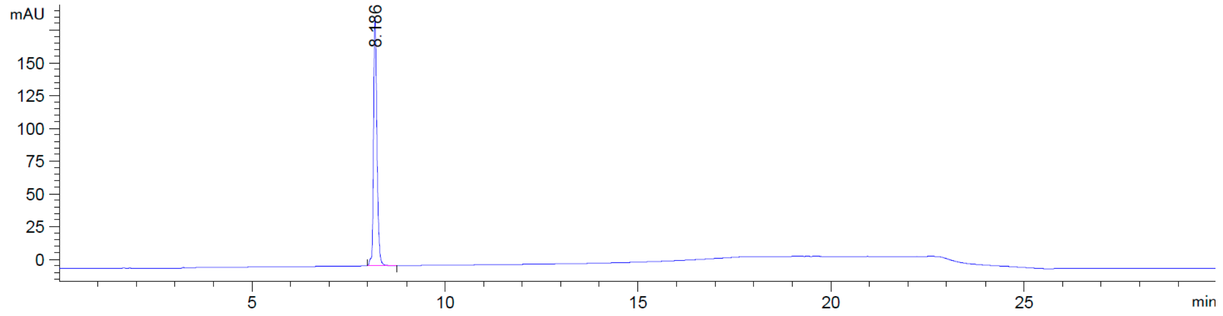

Figure S9: HPLC chromatogram of **3**.

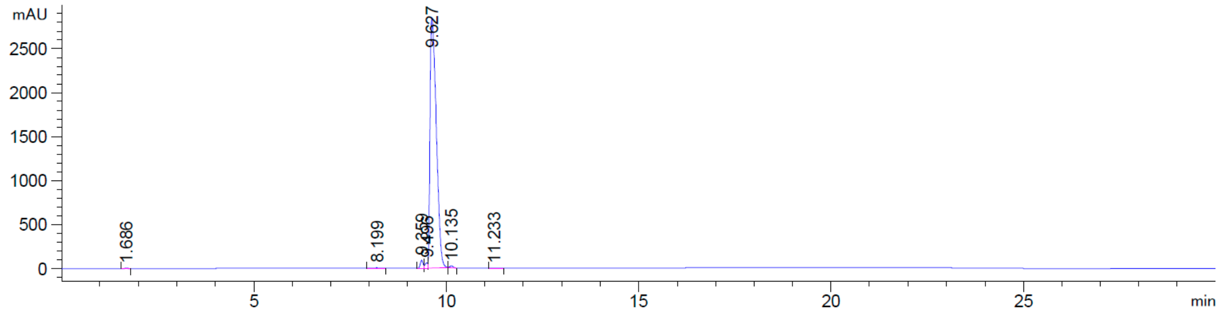

Figure S10: HPLC chromatogram of **4**.

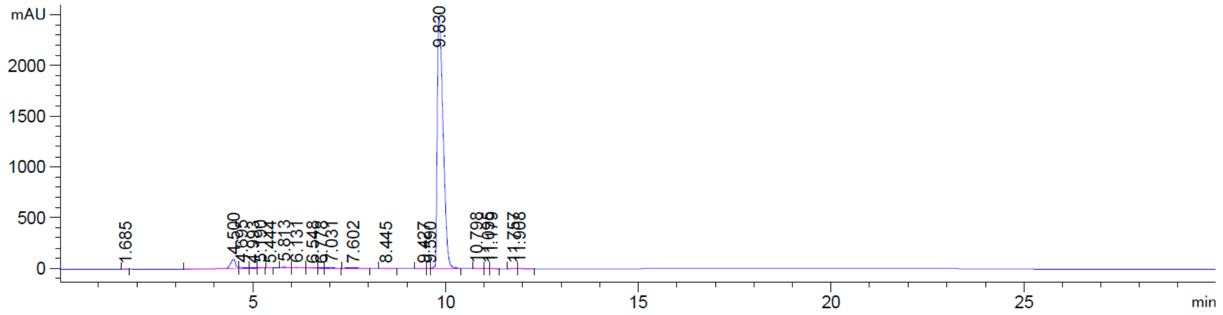

Figure S11: HPLC chromatogram of **5**.

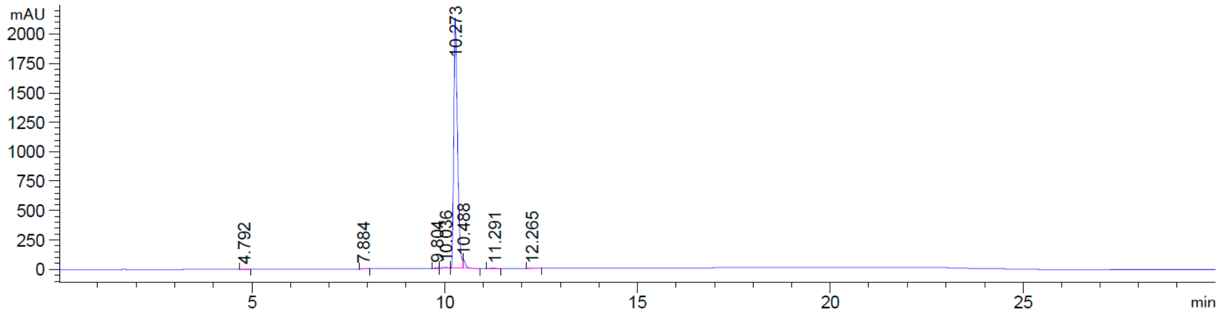

Figure S12: HPLC chromatogram of **6**.

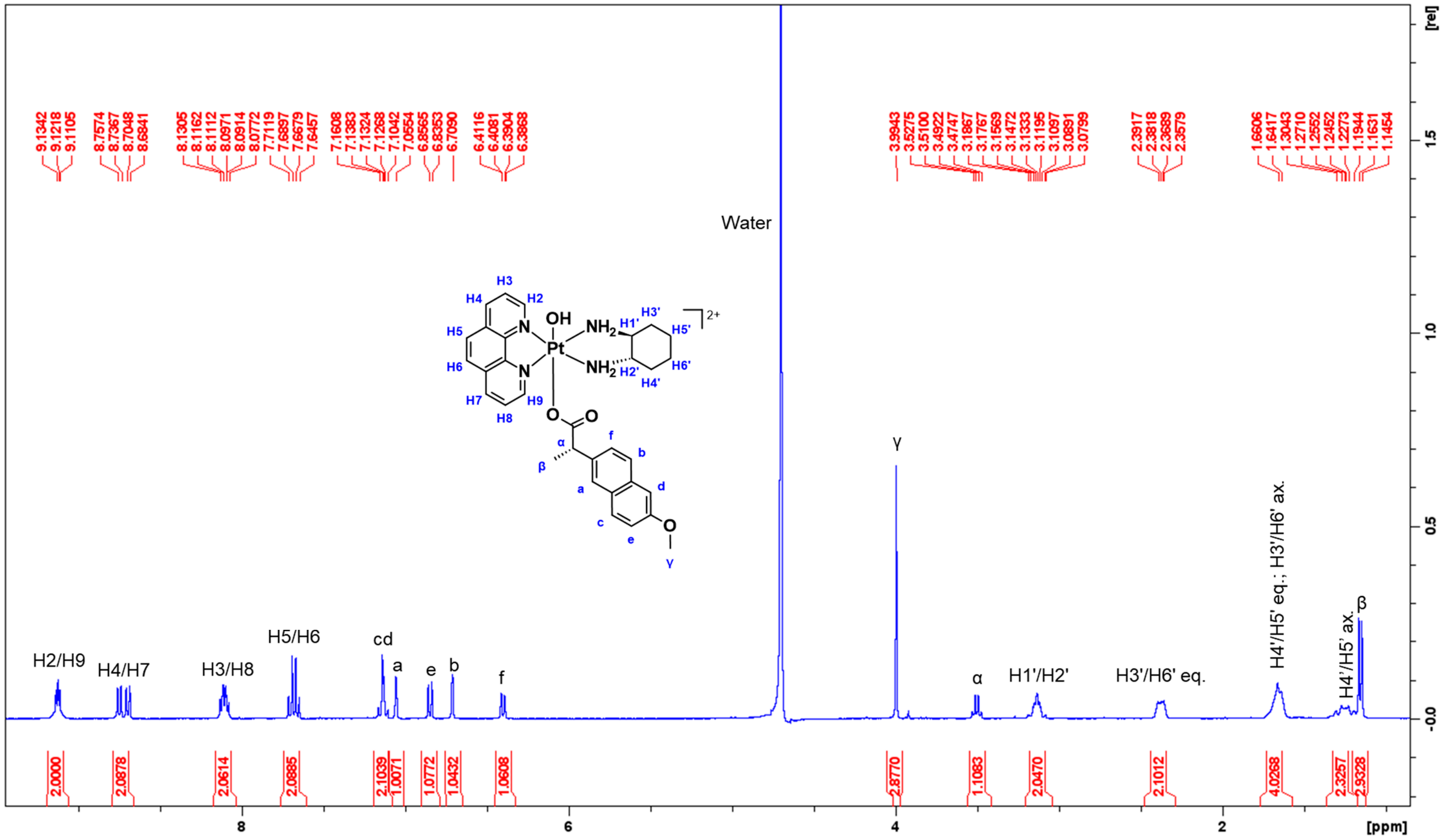

Figure S13: ^{1}H-NMR spectrum of **1** in D_2O.

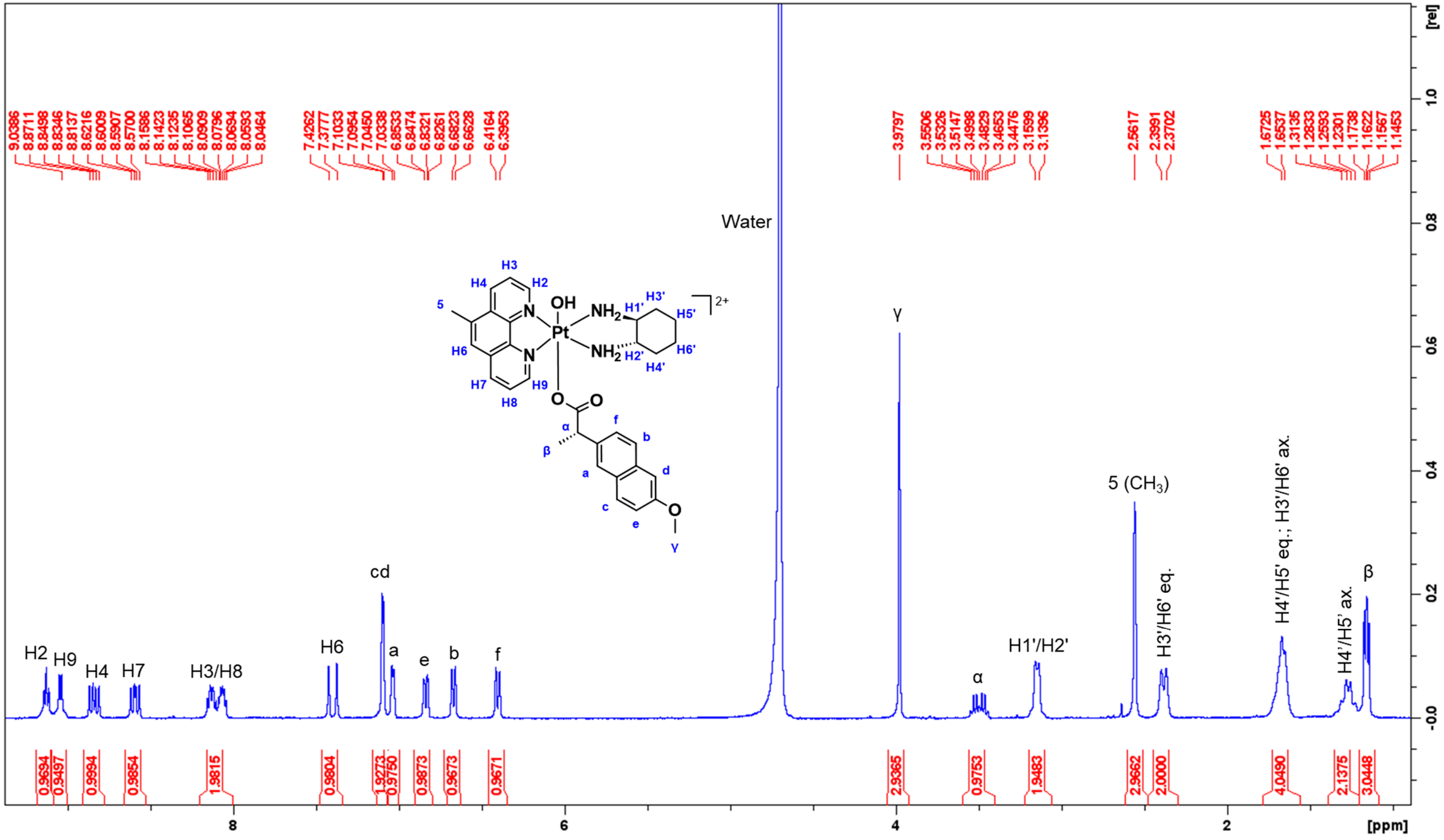

Figure S14: ^{1}H-NMR spectrum of **2** in D_2O.

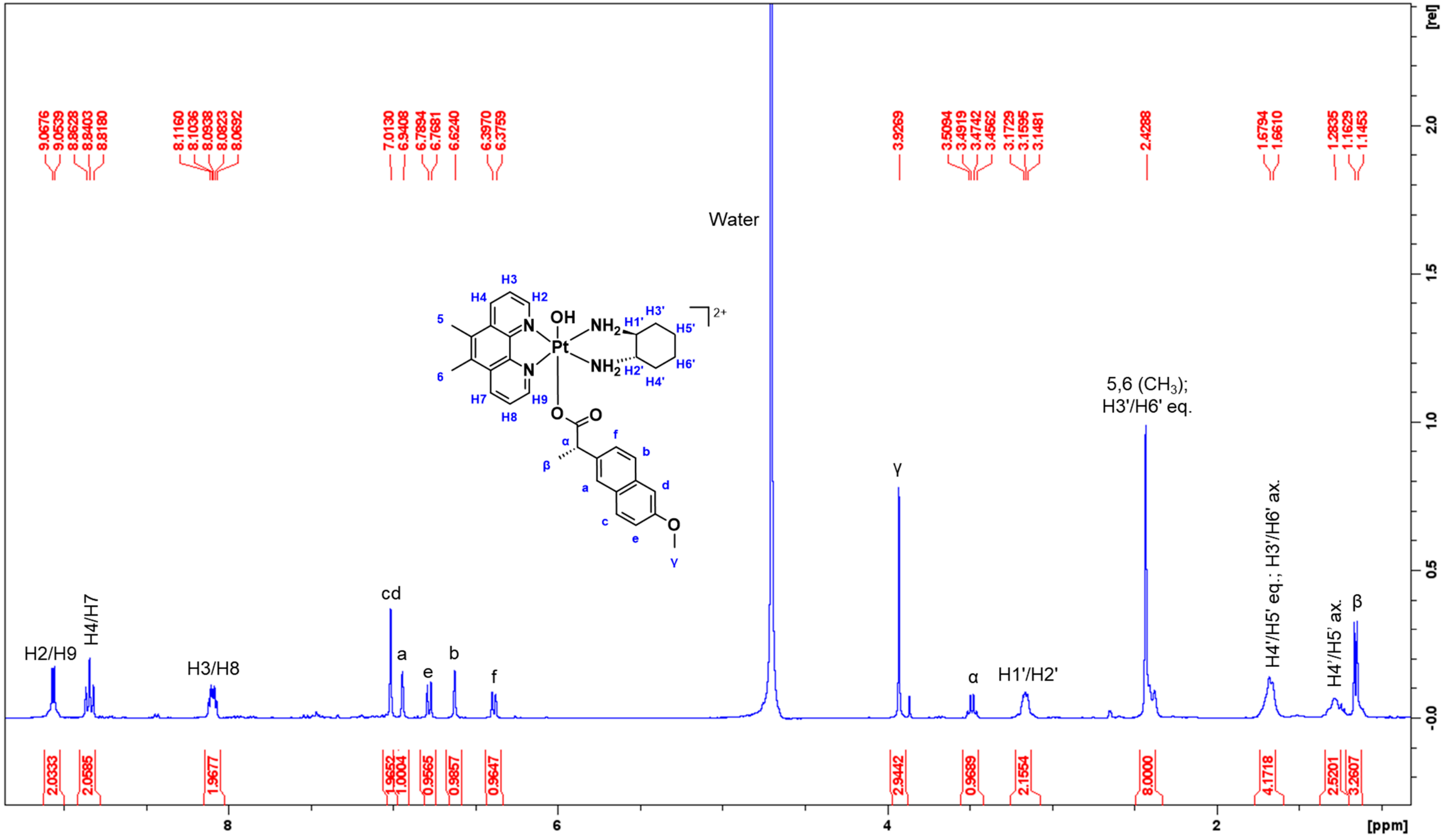

Figure S15: ^{1}H-NMR spectrum of **3** in D_2O.

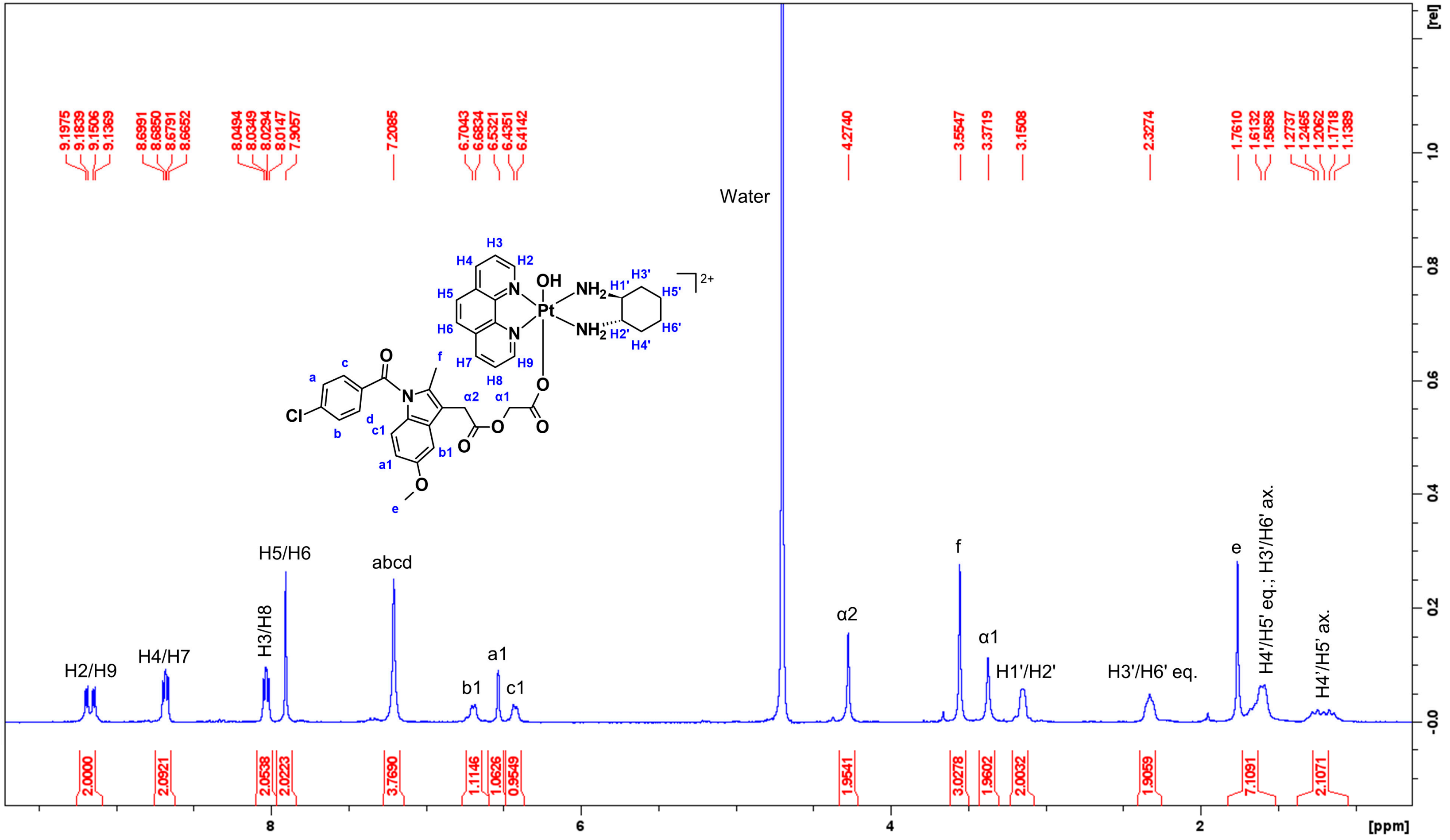

Figure S16: ^{1}H-NMR spectrum of **4** in D_2O.

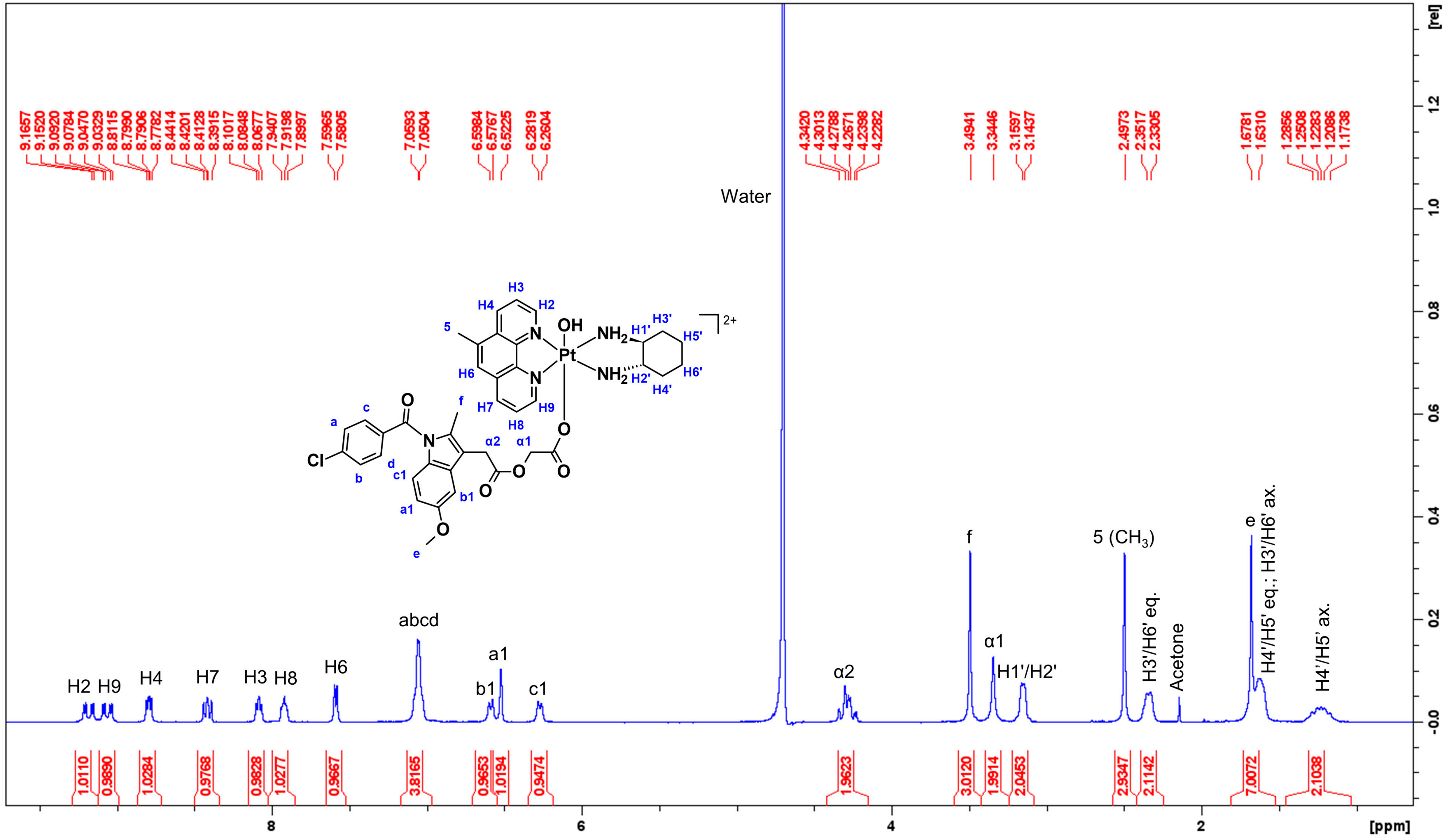

Figure S17: ^{1}H-NMR spectrum of **5** in D_2O.

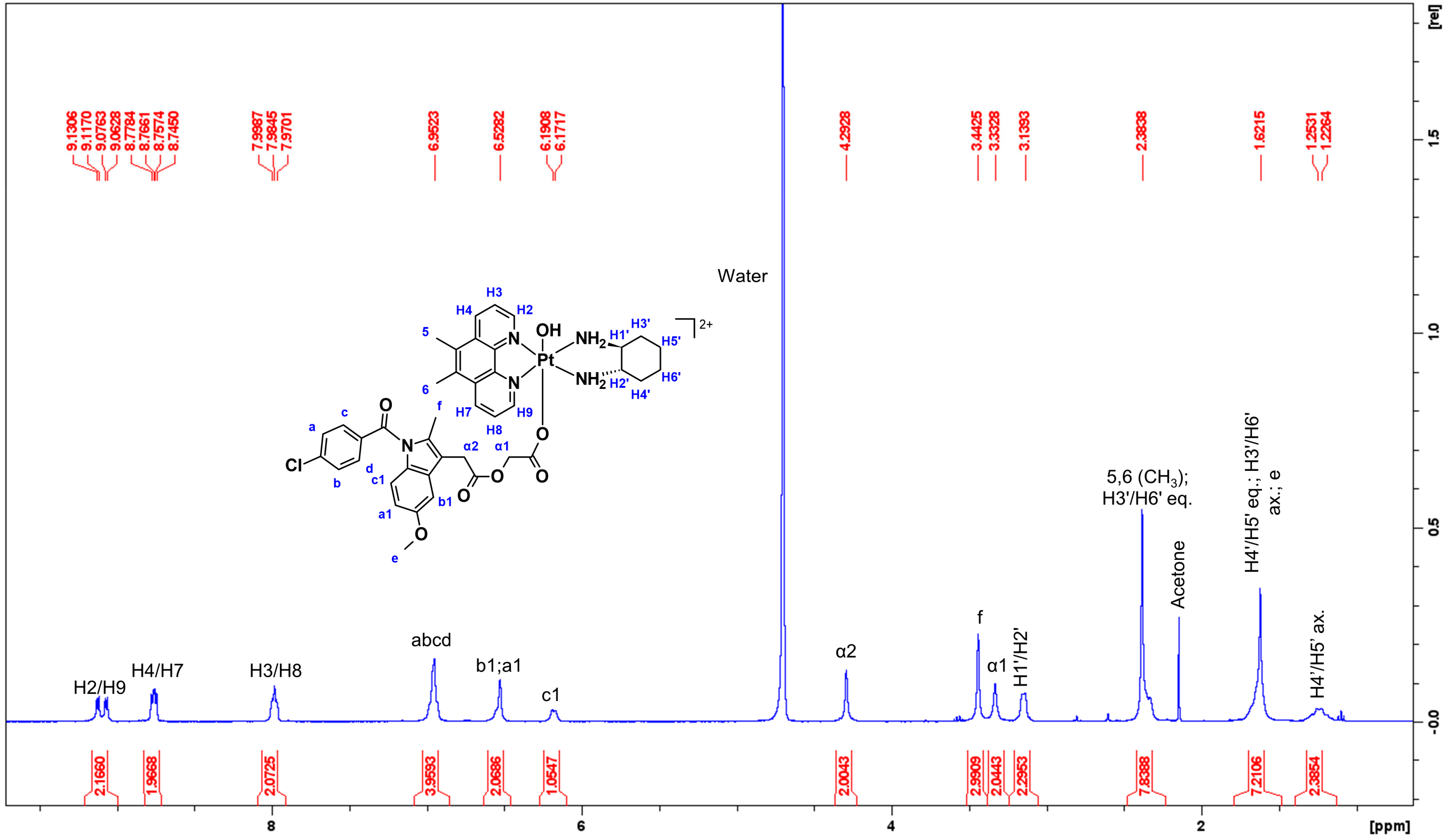

Figure S18: ^{1}H-NMR spectrum of **6** in D_2O.

2D-COSY Spectra of 1–6

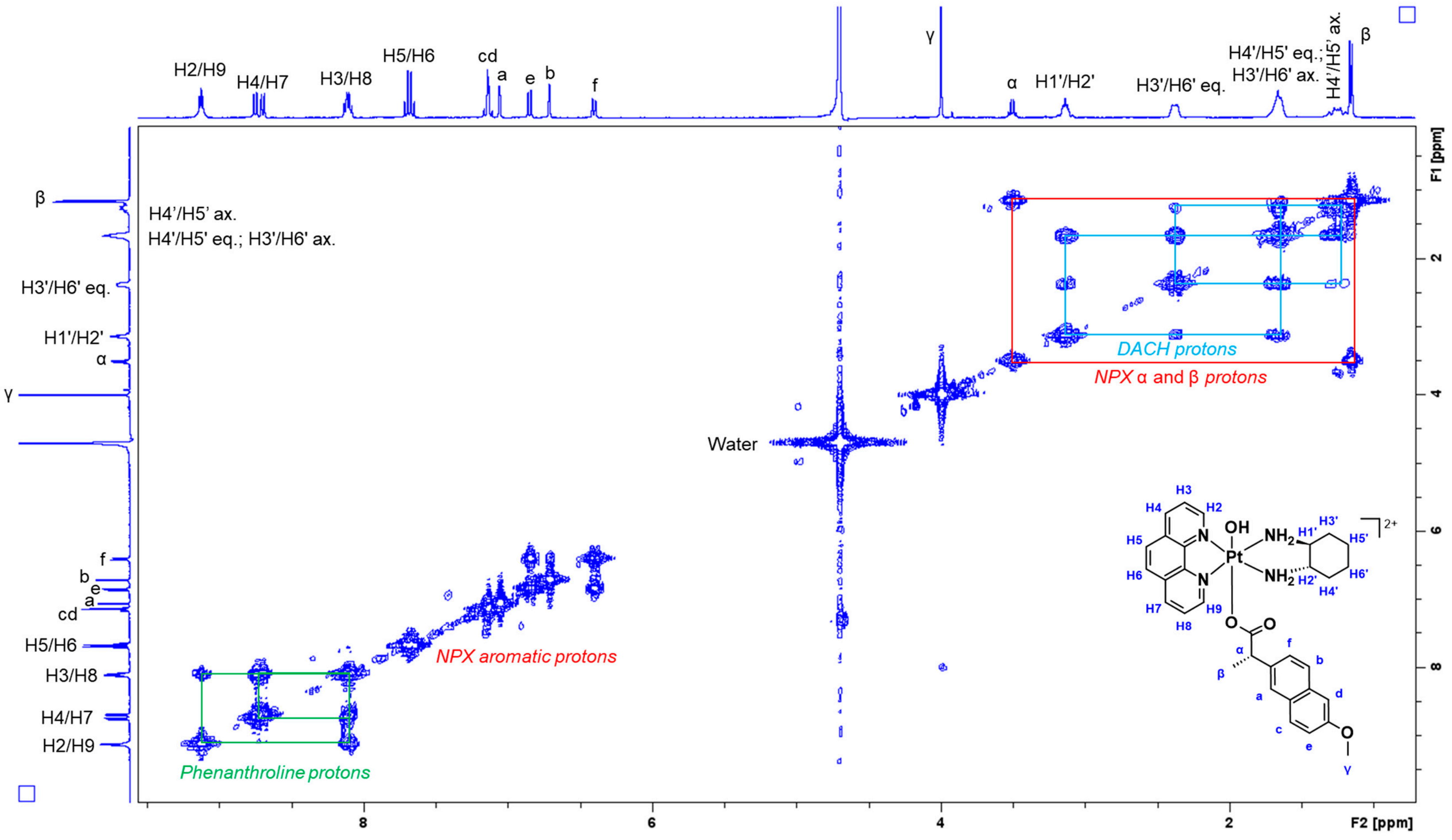

Figure S19: 2D-COSY spectrum of **1** in D_2O.

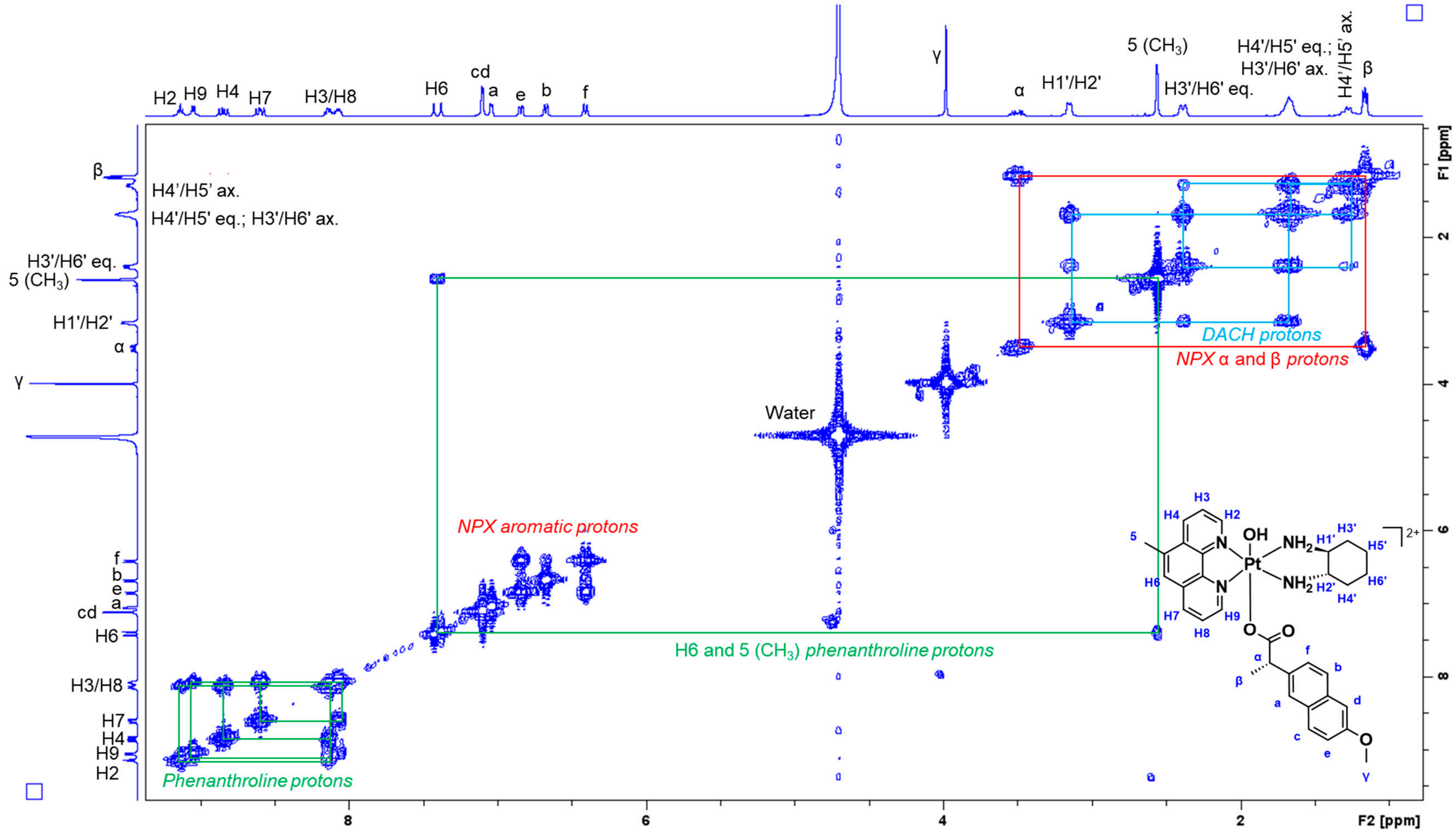

Figure S20: 2D-COSY spectrum of **2** in D_2O.

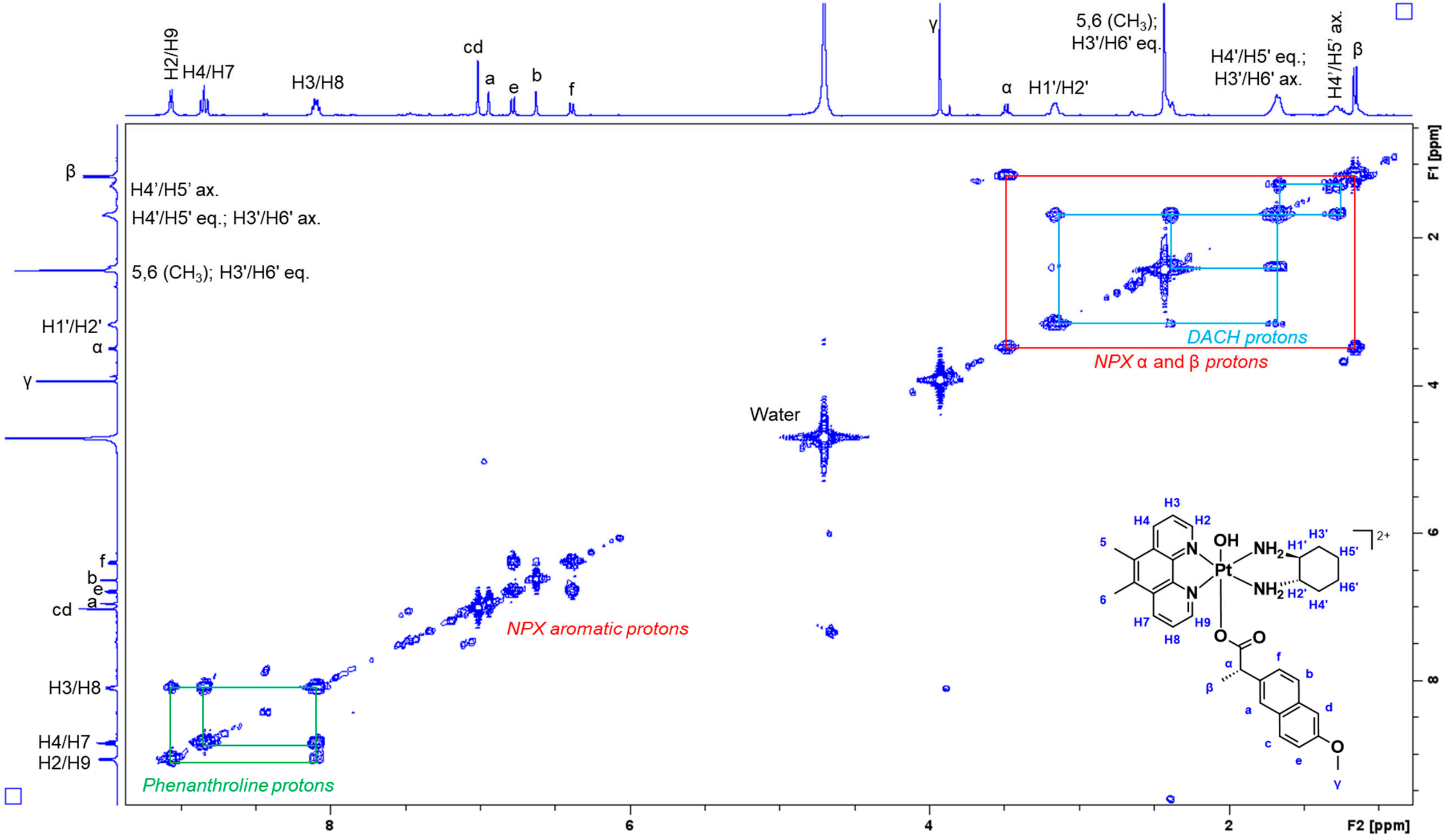

Figure S21: 2D-COSY spectrum of **3** in D_2O.

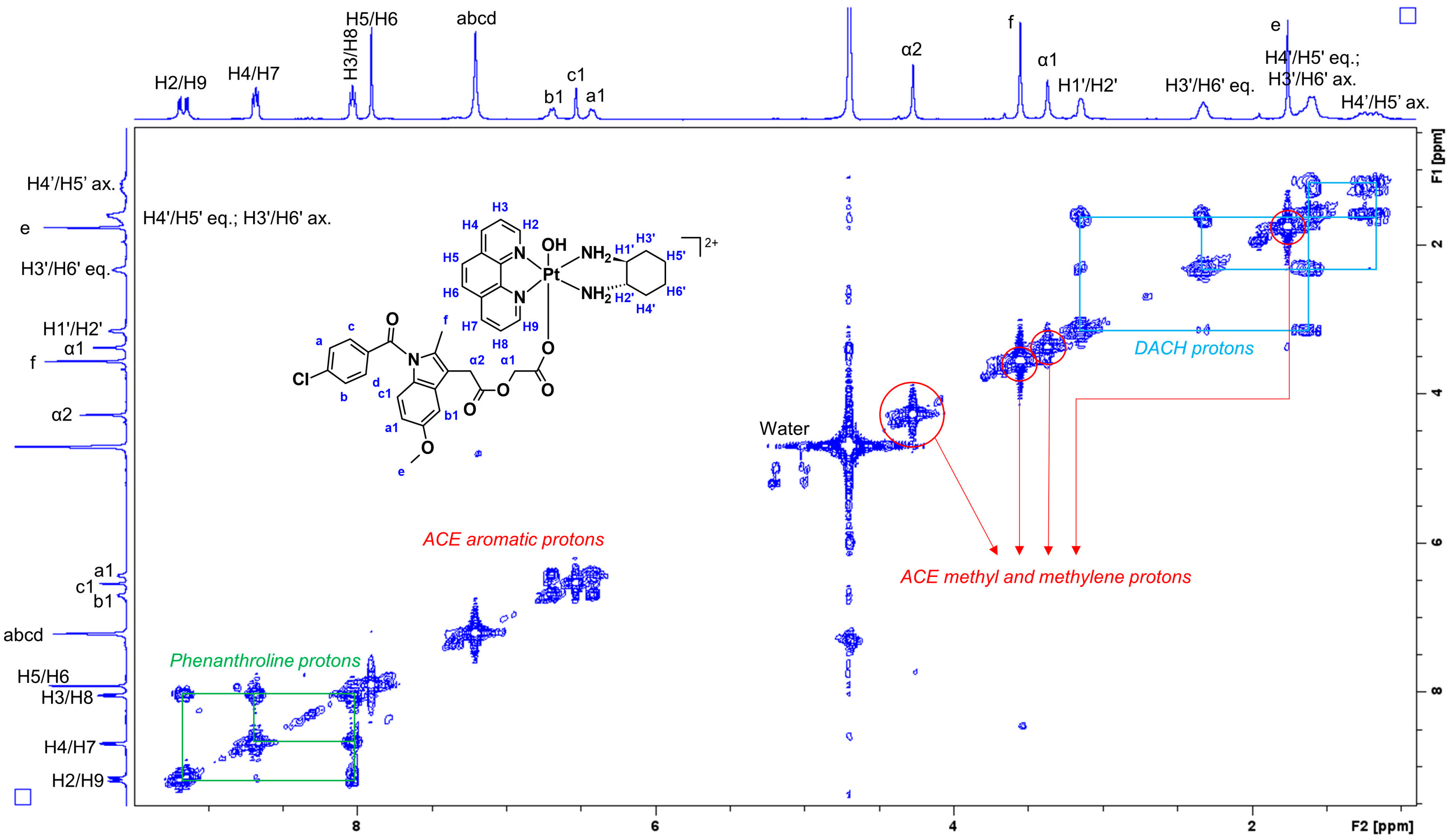

Figure S22: 2D-COSY spectrum of **4** in D_2O.

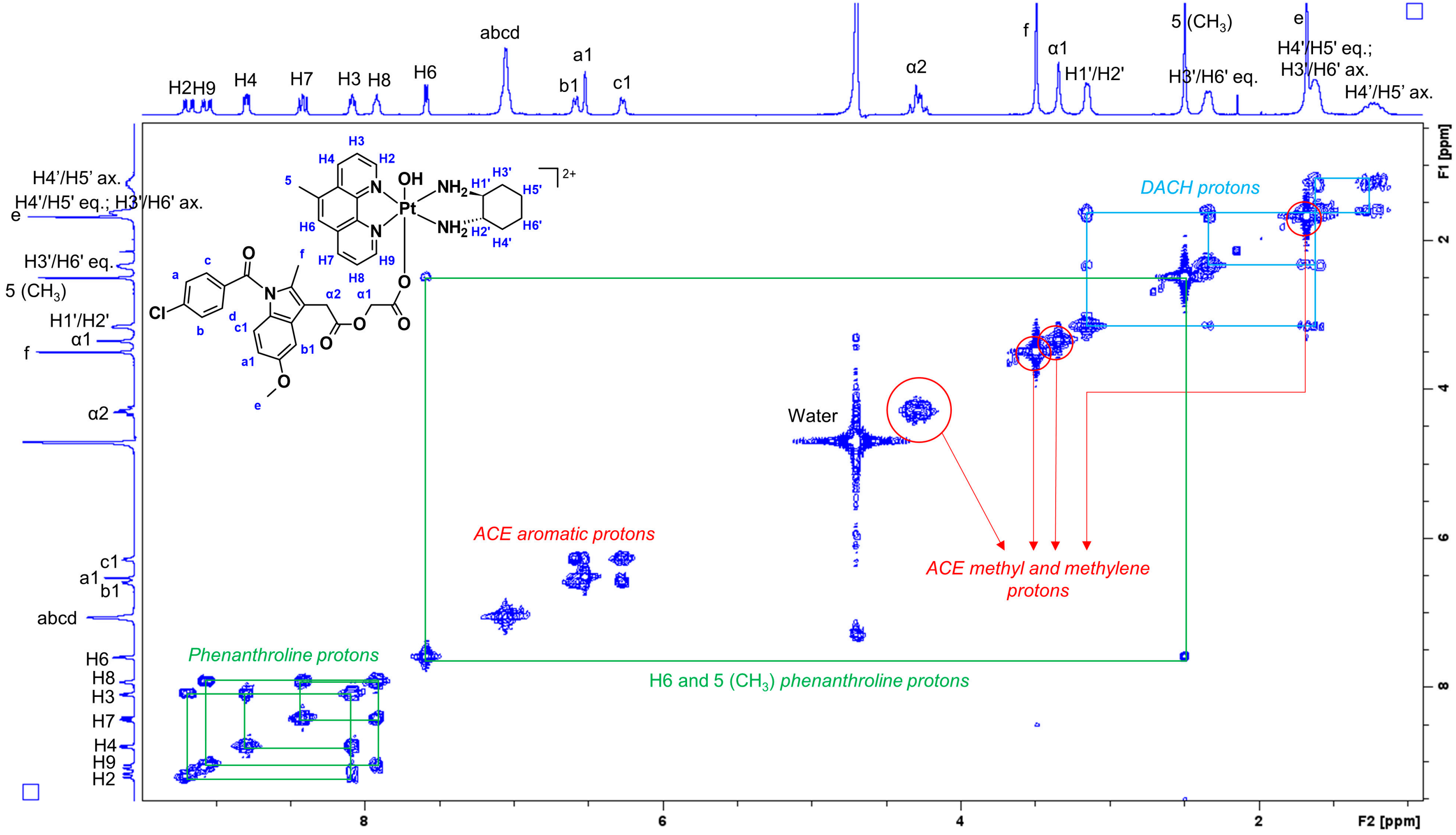

Figure S23: 2D-COSY spectrum of of **5** in D_2O.

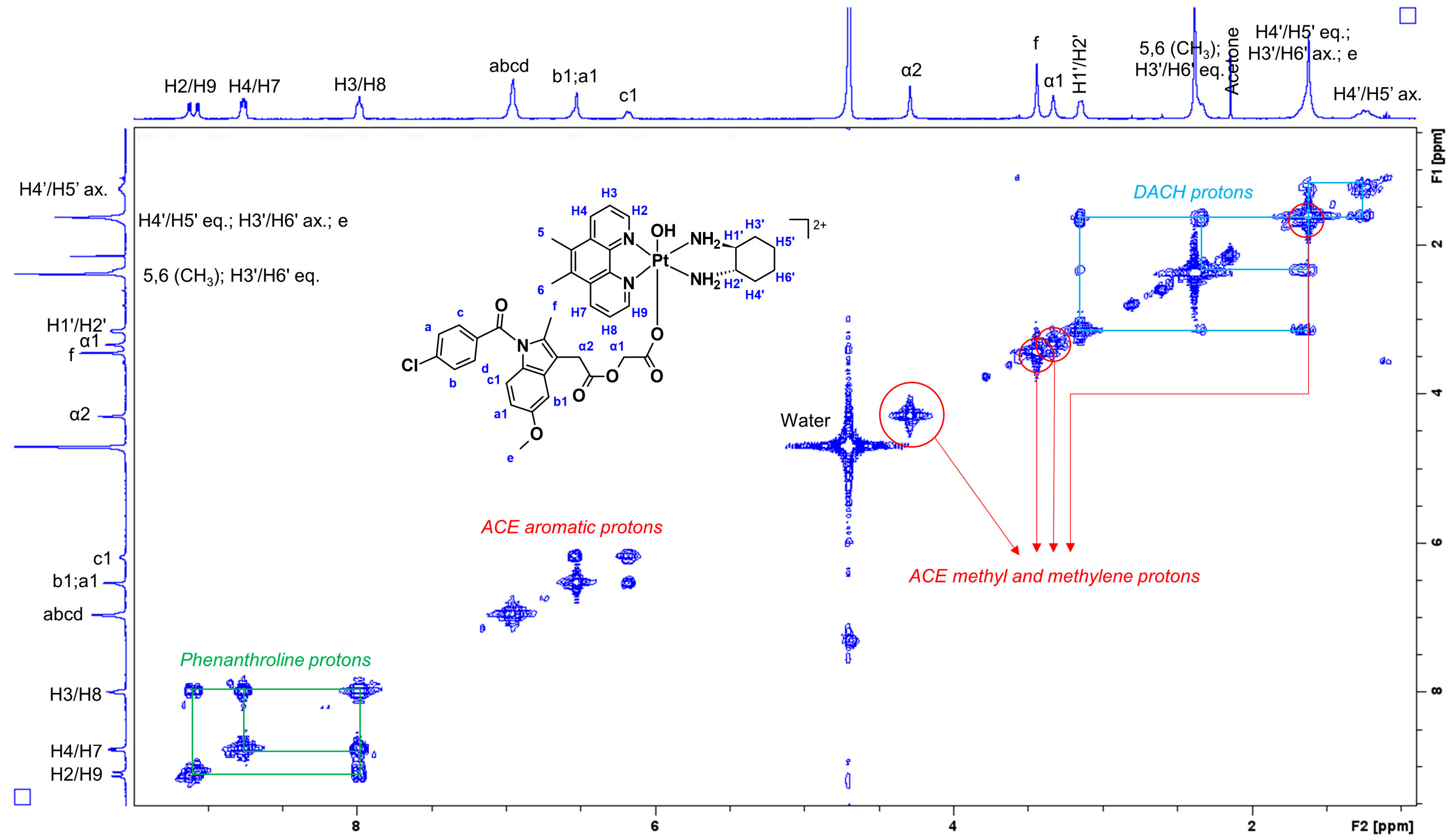

Figure S24: 2D-COSY spectrum of of **6** in D_2O.

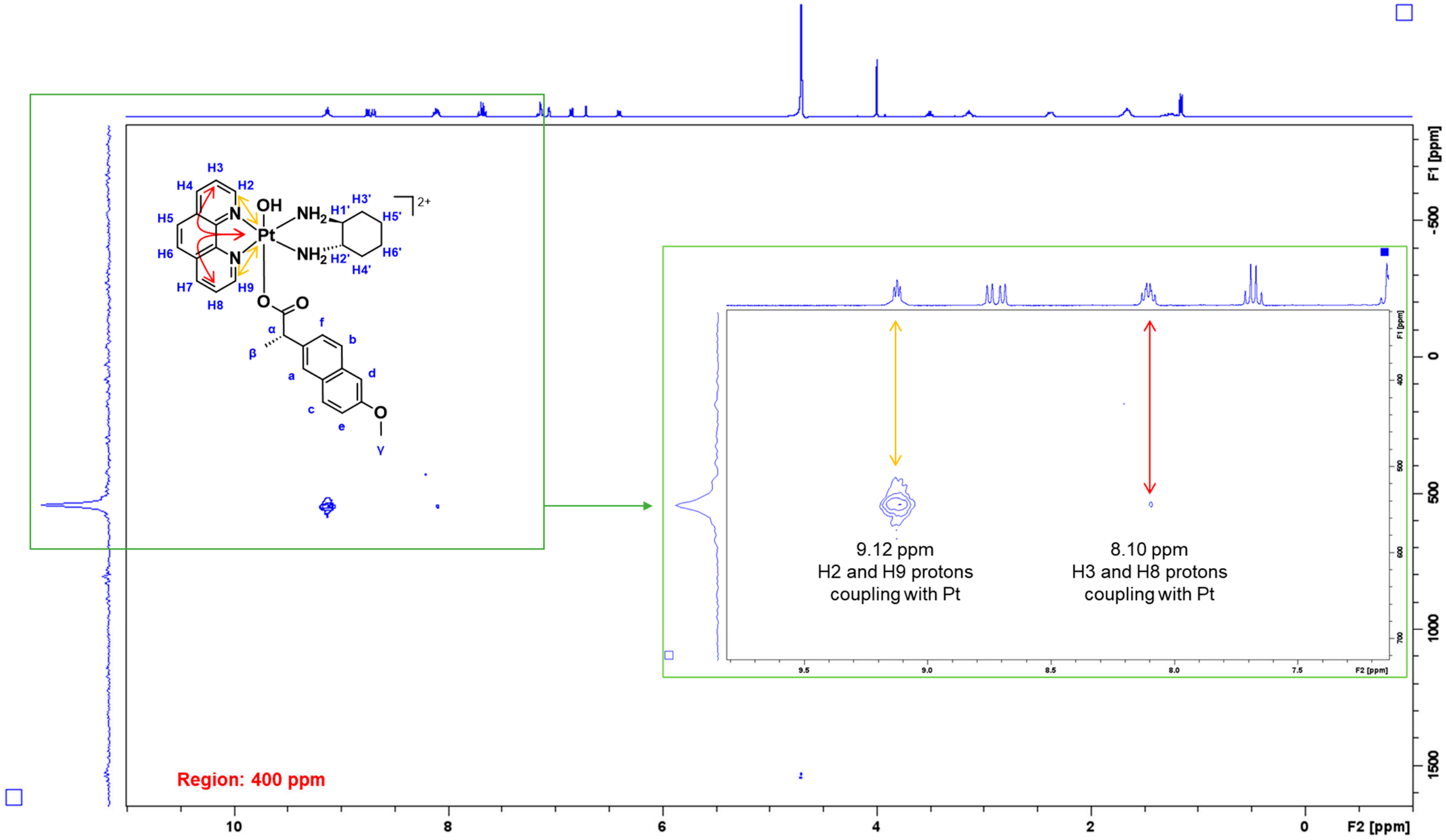

Figure S25: ^{1}H-^{195}Pt-HMQC spectrum of **1** in D_2O at 400 ppm.

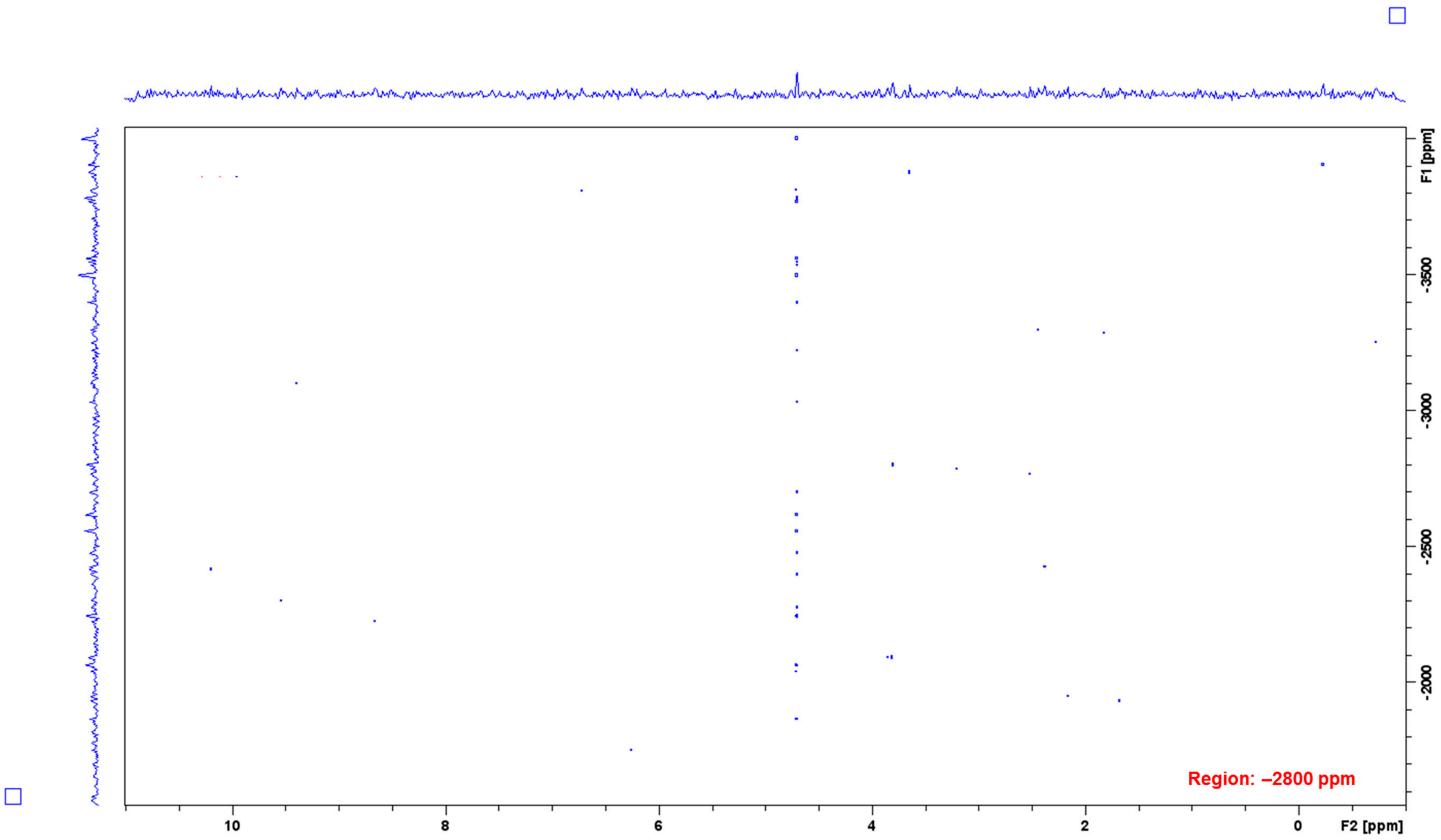

Figure S26: ^{1}H-^{195}Pt-HMQC spectrum of **1** in D_2O at –2800 ppm.

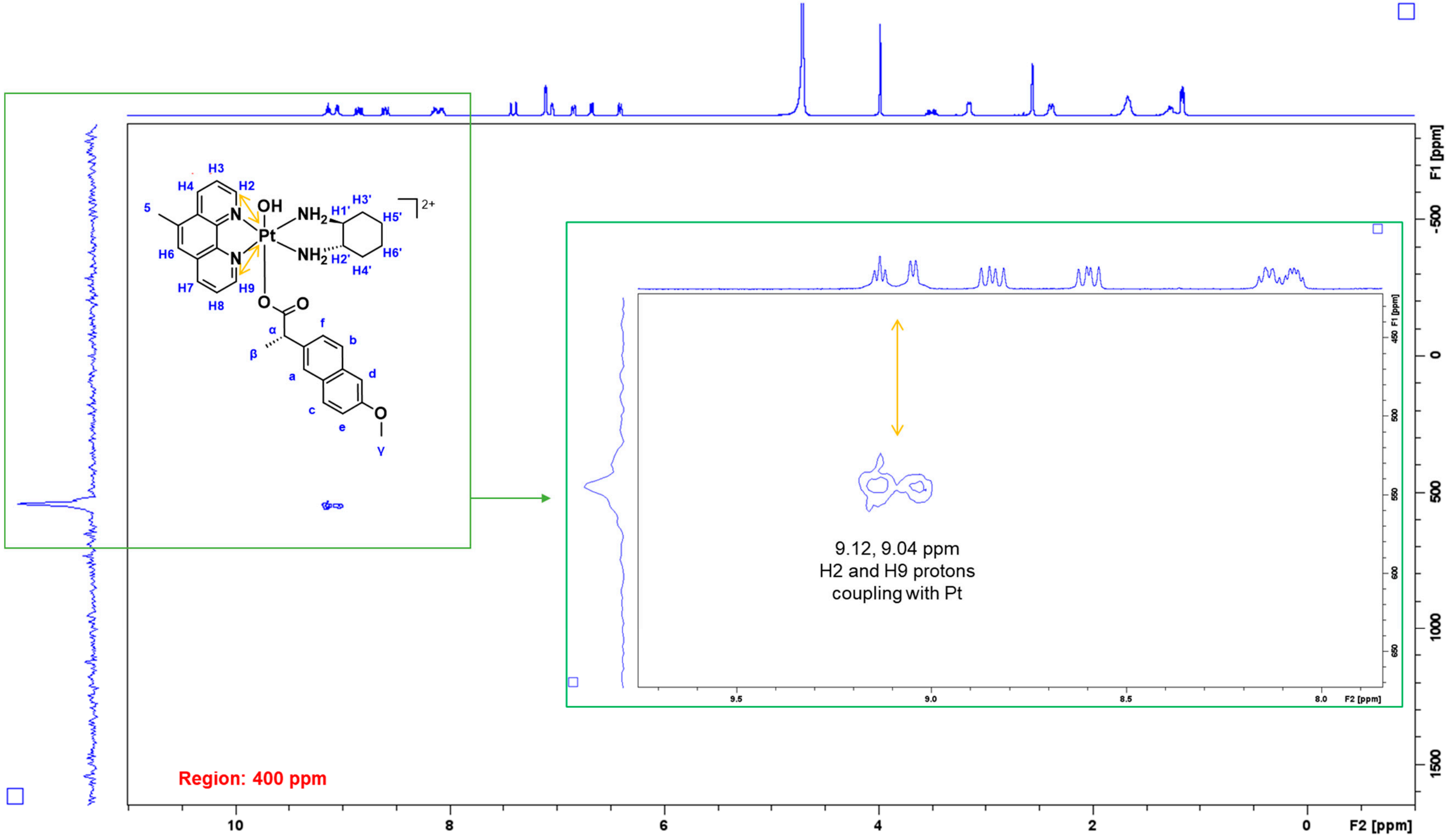

Figure S27: ^{1}H-^{195}Pt-HMQC spectrum of **2** in D_2O at 400 ppm.

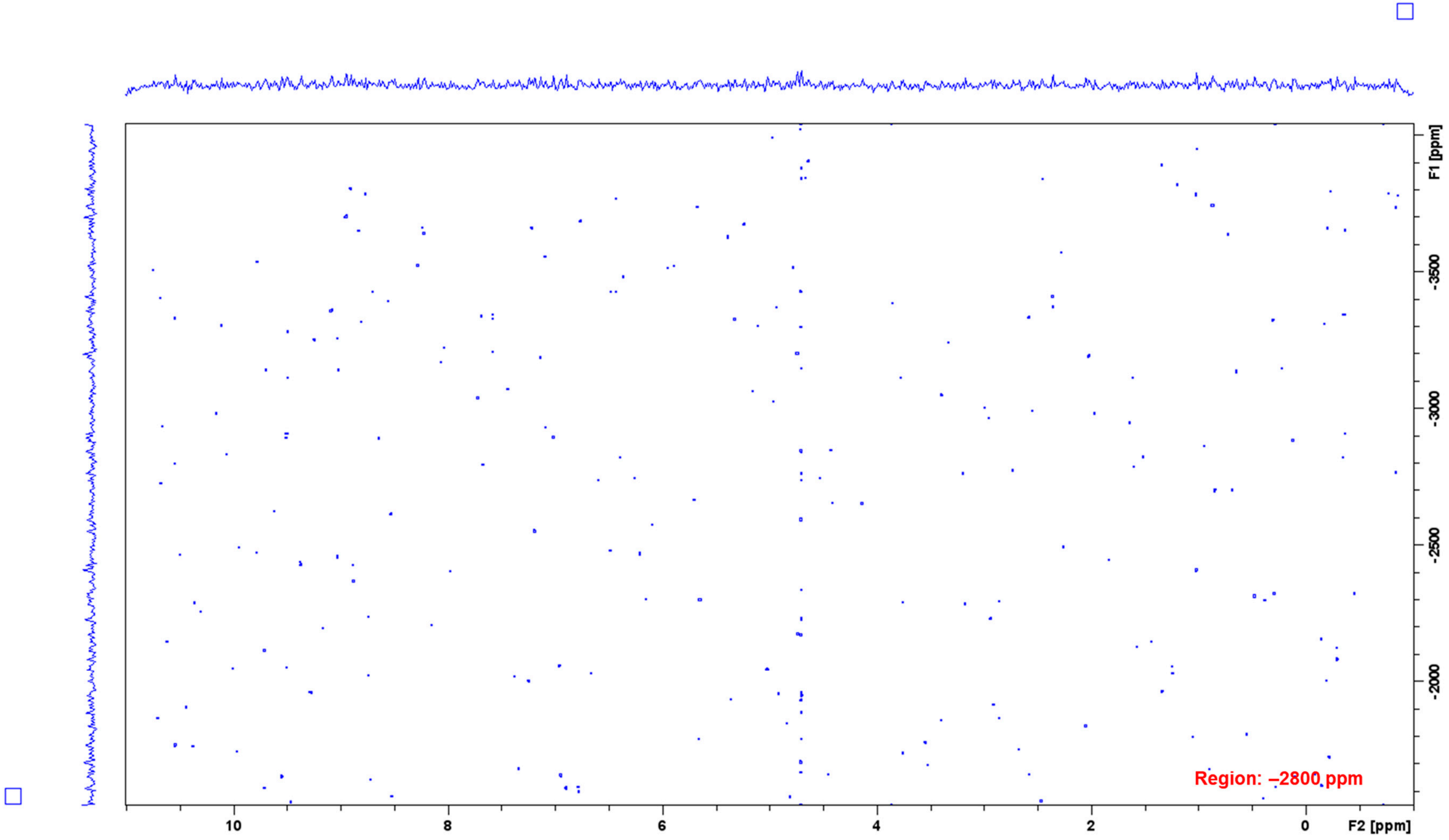

Figure S28: ^{1}H-^{195}Pt-HMQC spectrum of **2** in D_2O at –2800 ppm.

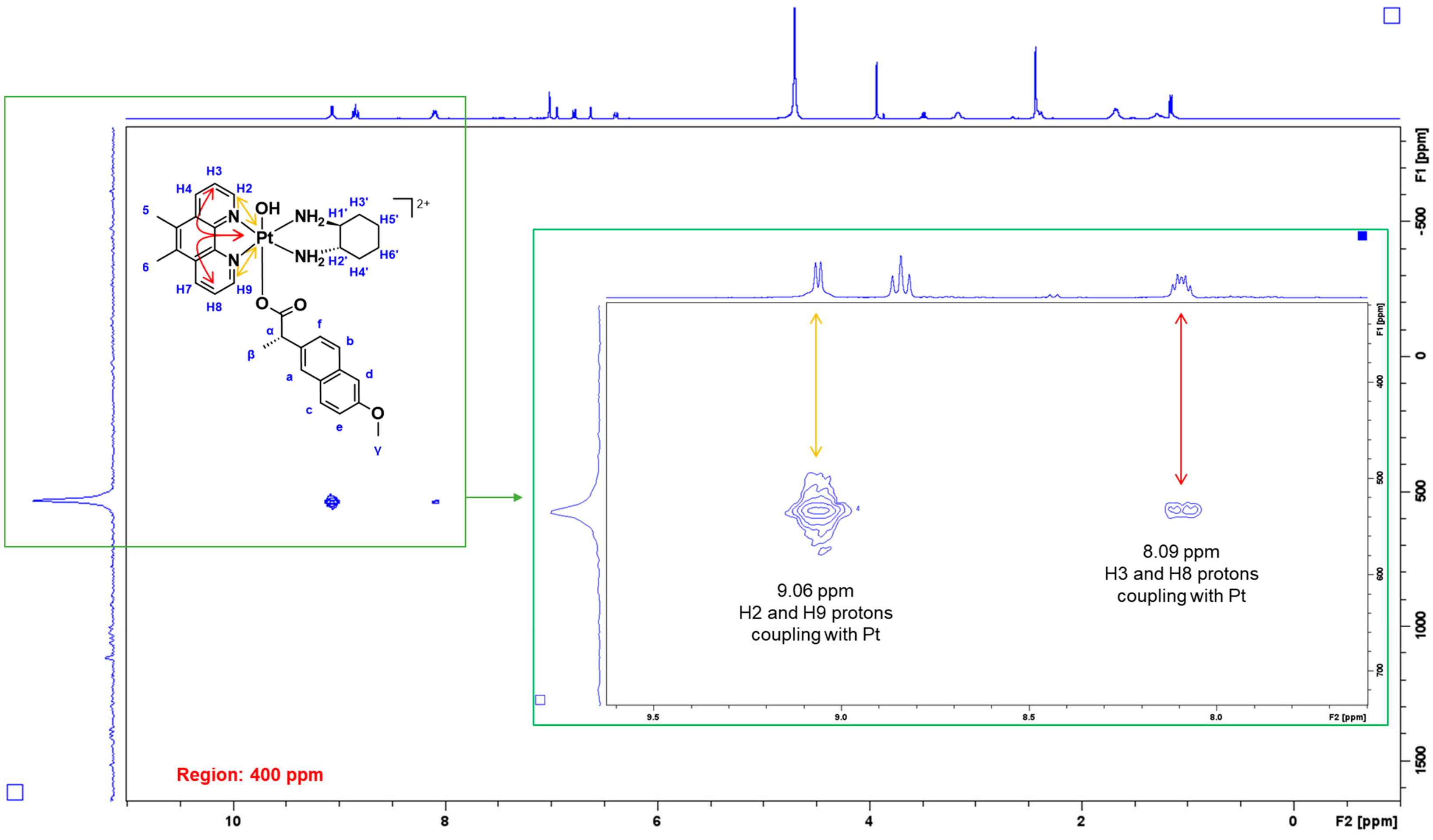

Figure S29: ^{1}H-^{195}Pt-HMQC spectrum of **3** in D_2O at 400 ppm.

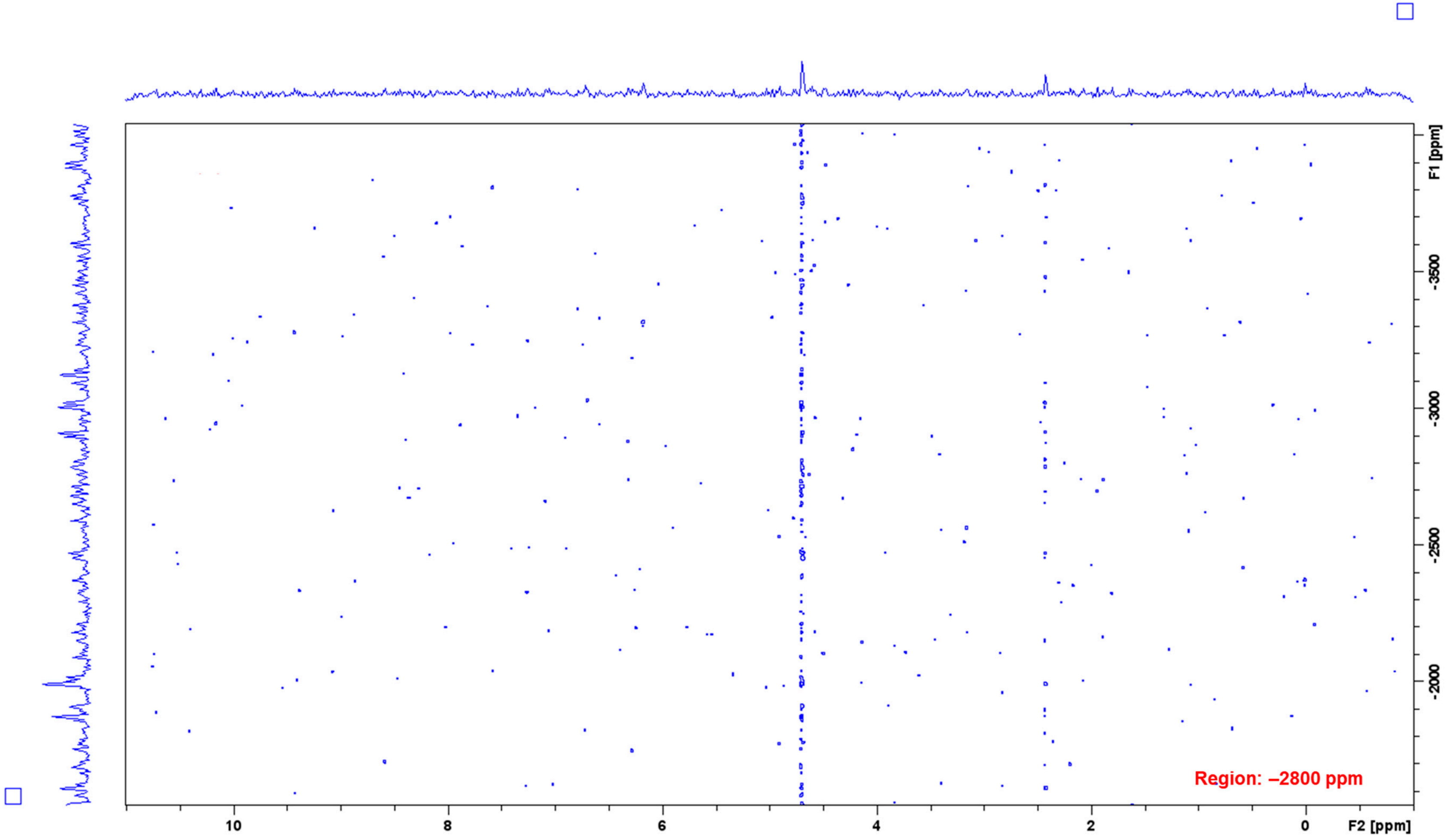

Figure S30: ^{1}H-^{195}Pt-HMQC spectrum of **3** in D_2O at –2800 ppm.

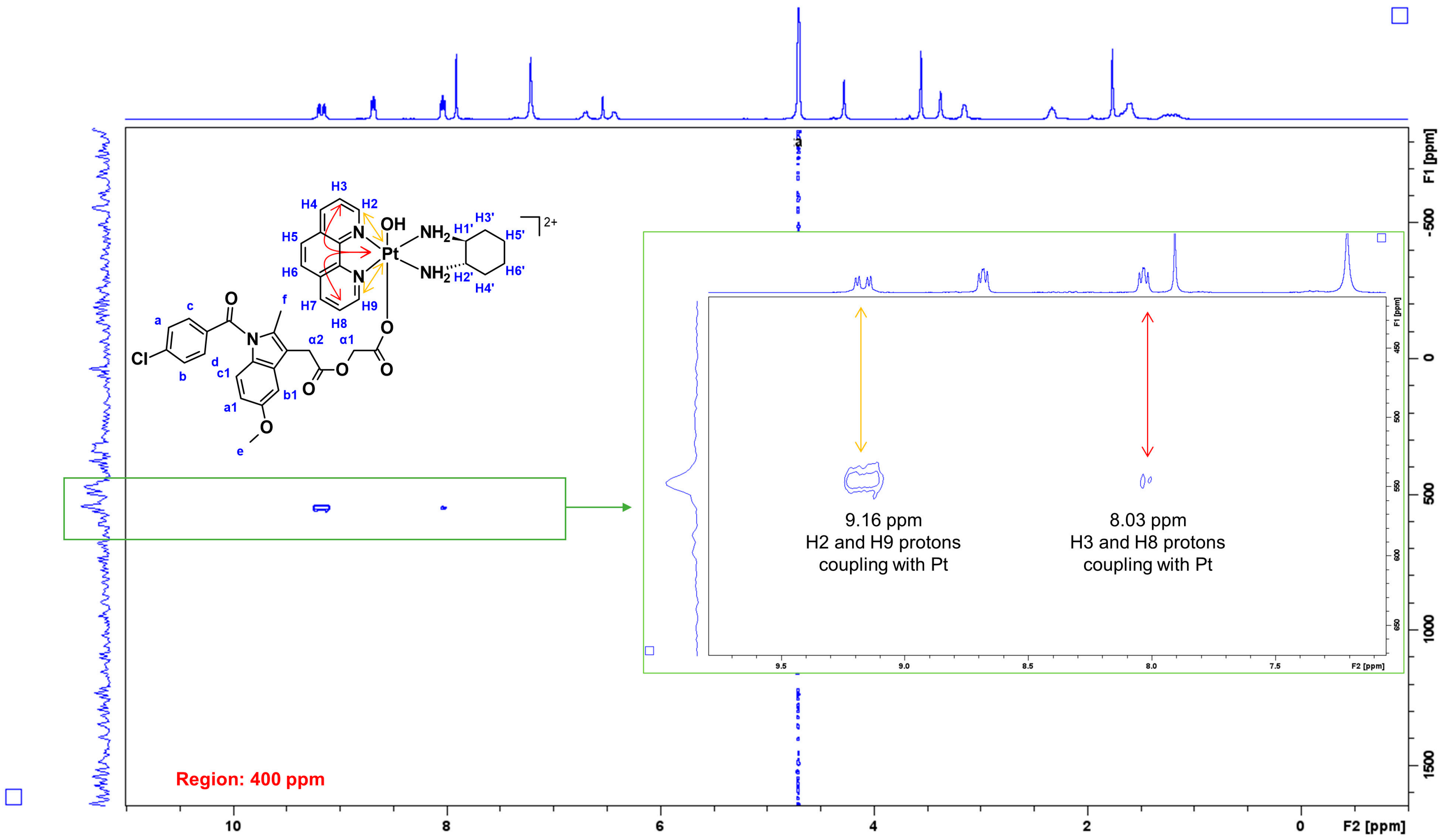

Figure S31: ^{1}H-^{195}Pt-HMQC spectrum of **4** in D_2O at 400 ppm.

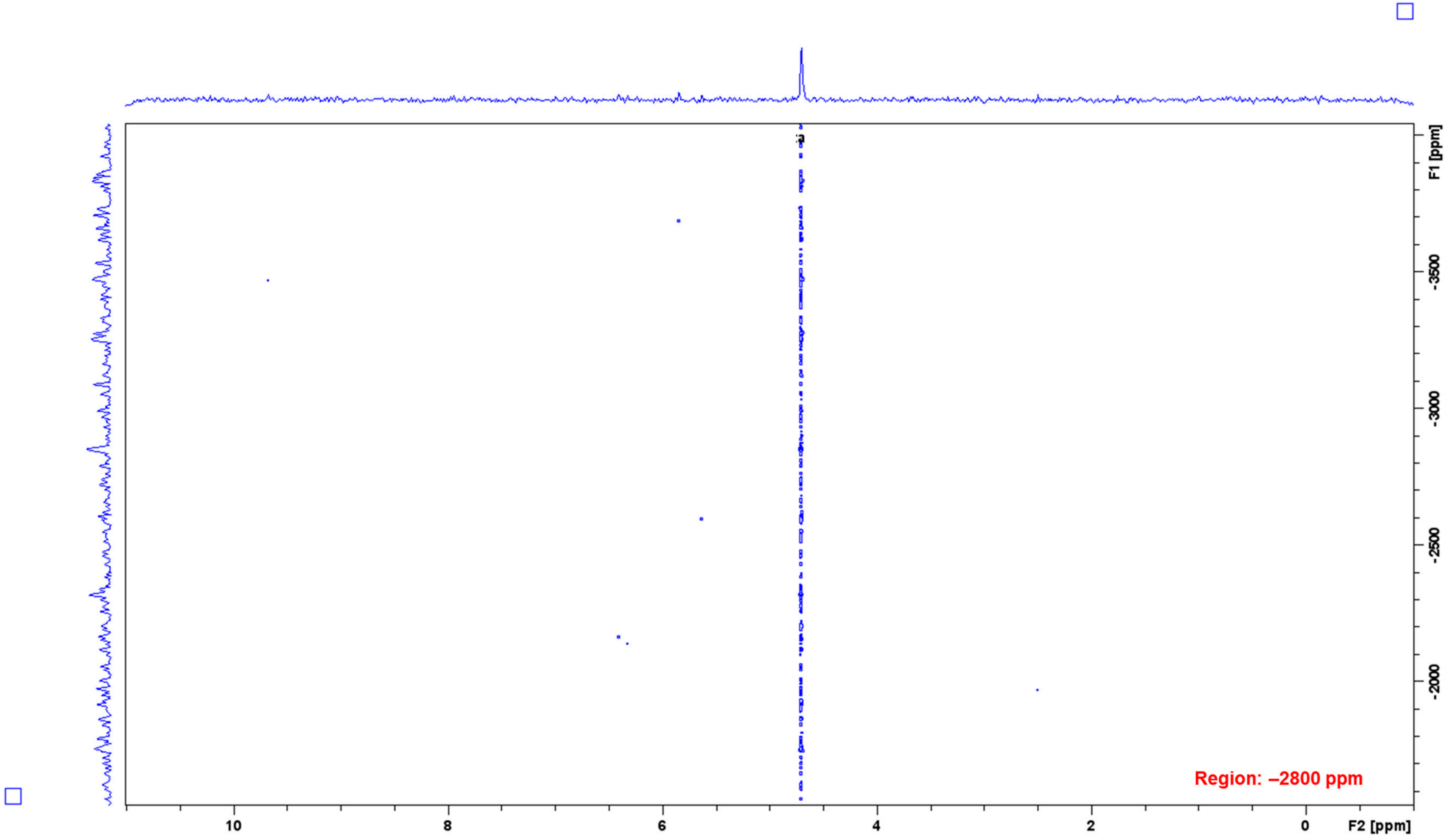

Figure S32: ^{1}H-^{195}Pt-HMQC spectrum of **4** in D_2O at –2800 ppm.

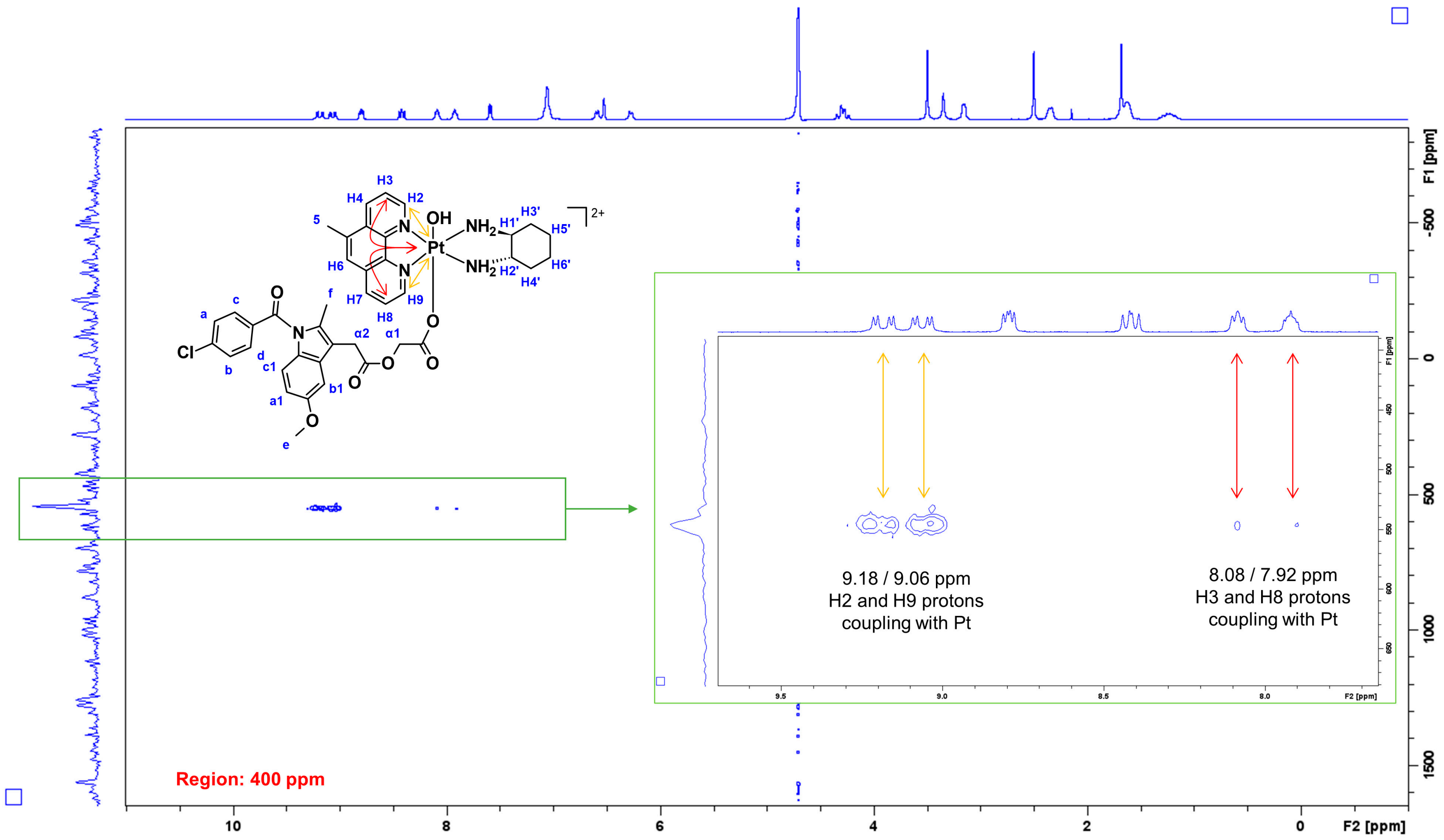

Figure S33: ^{1}H-^{195}Pt-HMQC spectrum of **5** in D_2O at 400 ppm.

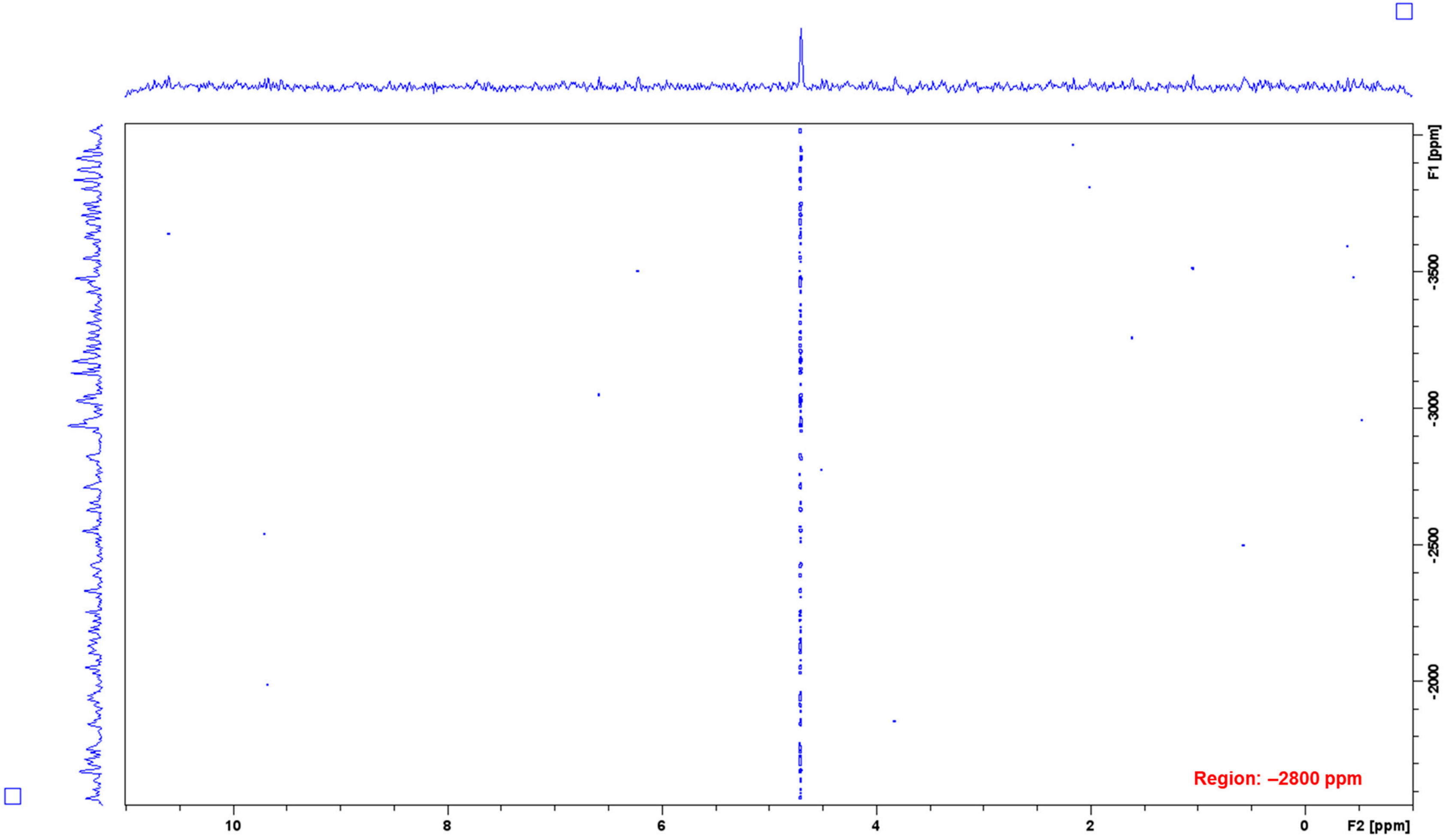

Figure S34: ^{1}H-^{195}Pt-HMQC spectrum of **5** in D_2O at –2800 ppm.

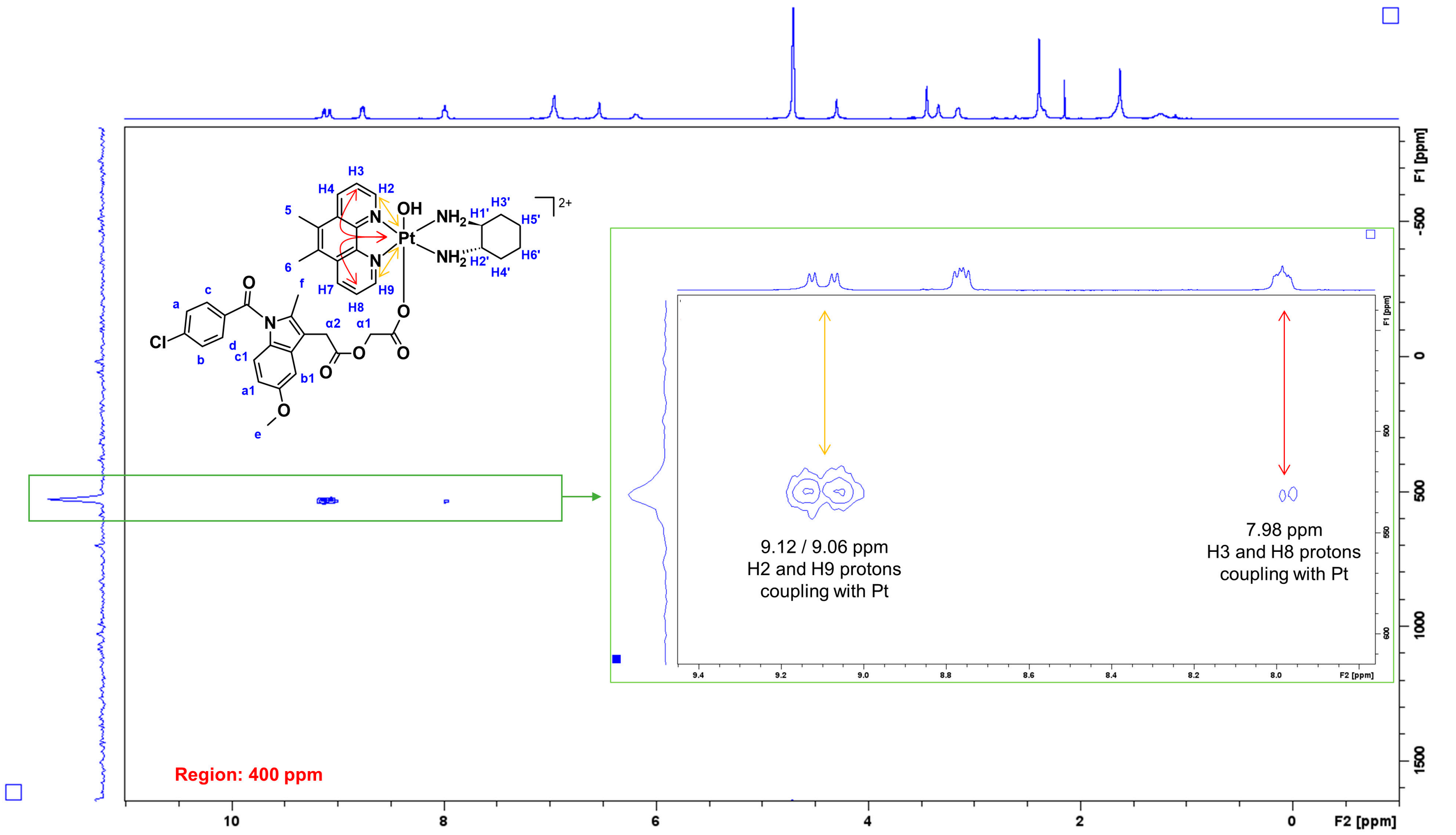

Figure S35: ^{1}H-^{195}Pt-HMQC spectrum of **6** in D_2O at 400 ppm.

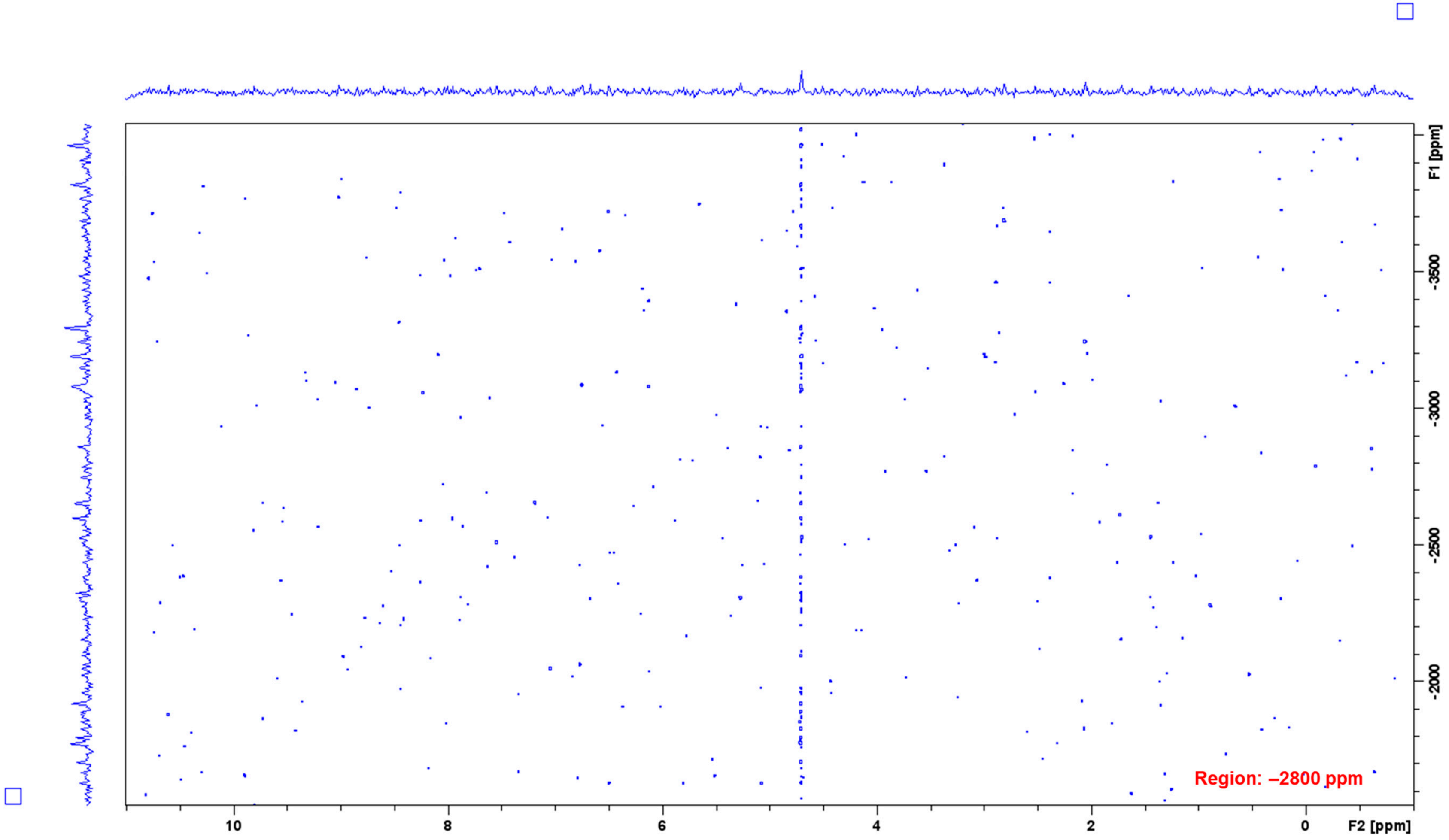

Figure S36: ^{1}H-^{195}Pt-HMQC spectrum of **6** in D_2O at –2800 ppm.

UV Spectra of 1–6

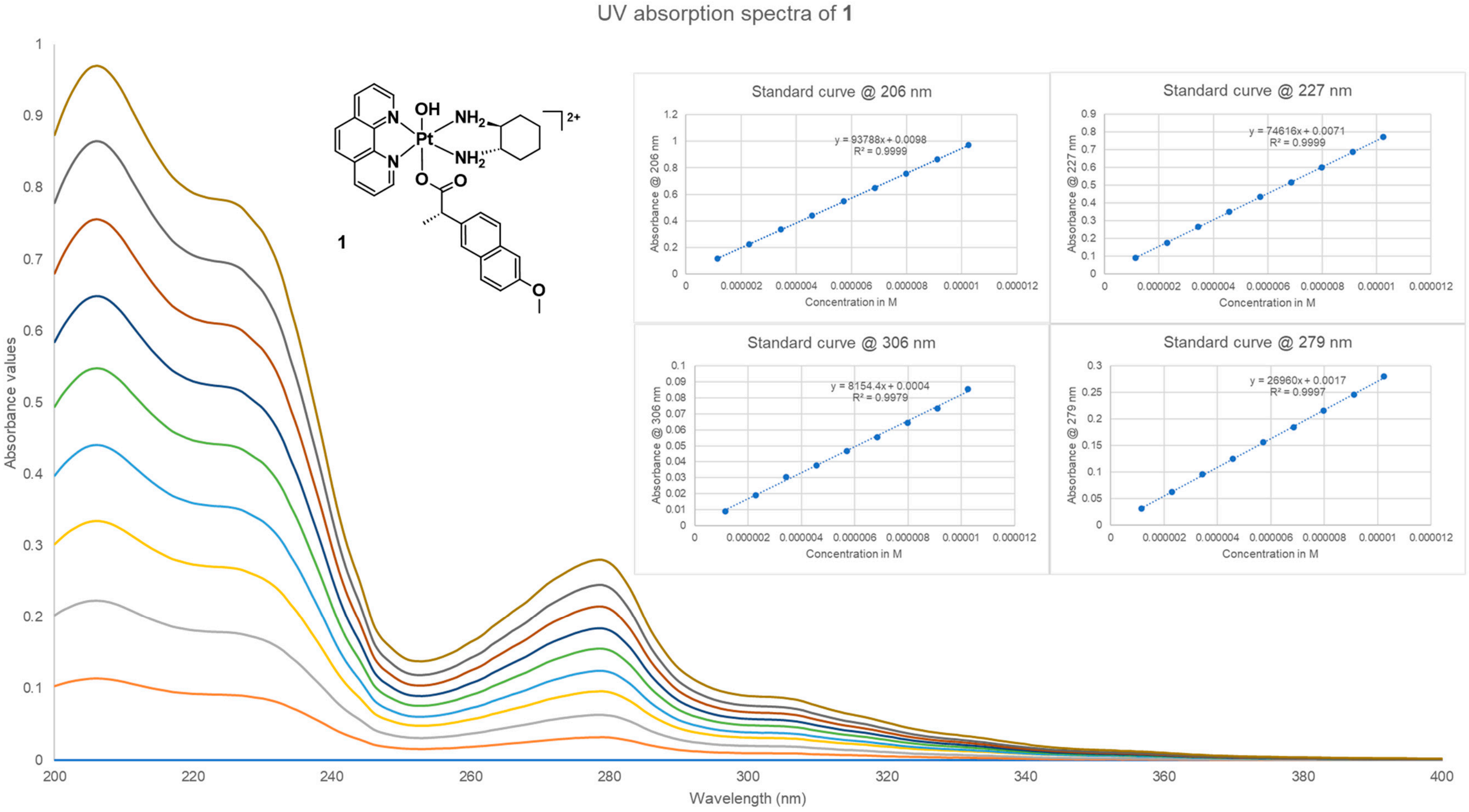

Figure S37: UV spectra of **1** and plot curves at 206, 227, 279 and 306 nm.

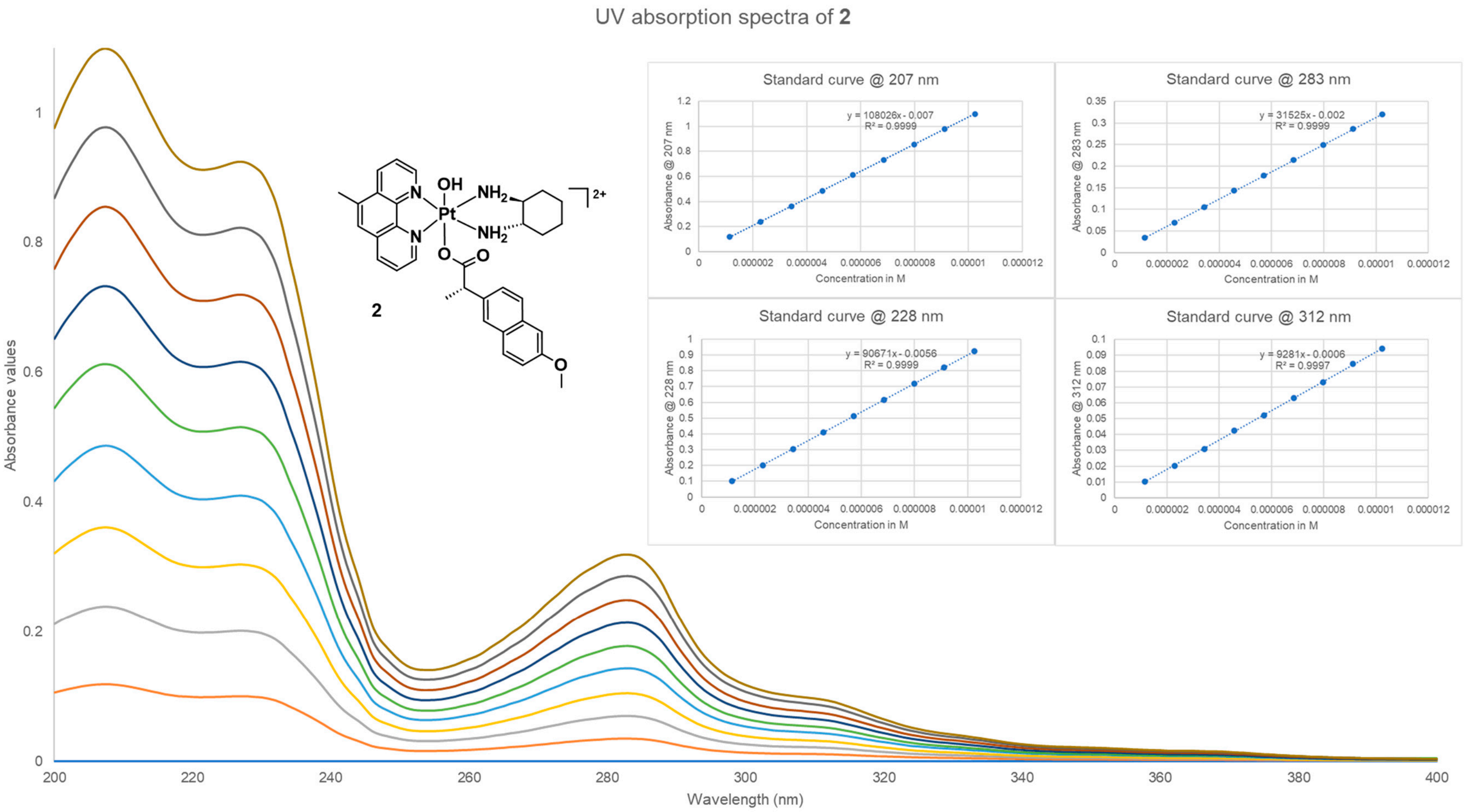

Figure S38: UV spectra of **2** and plot curves at 207, 228, 283 and 312 nm.

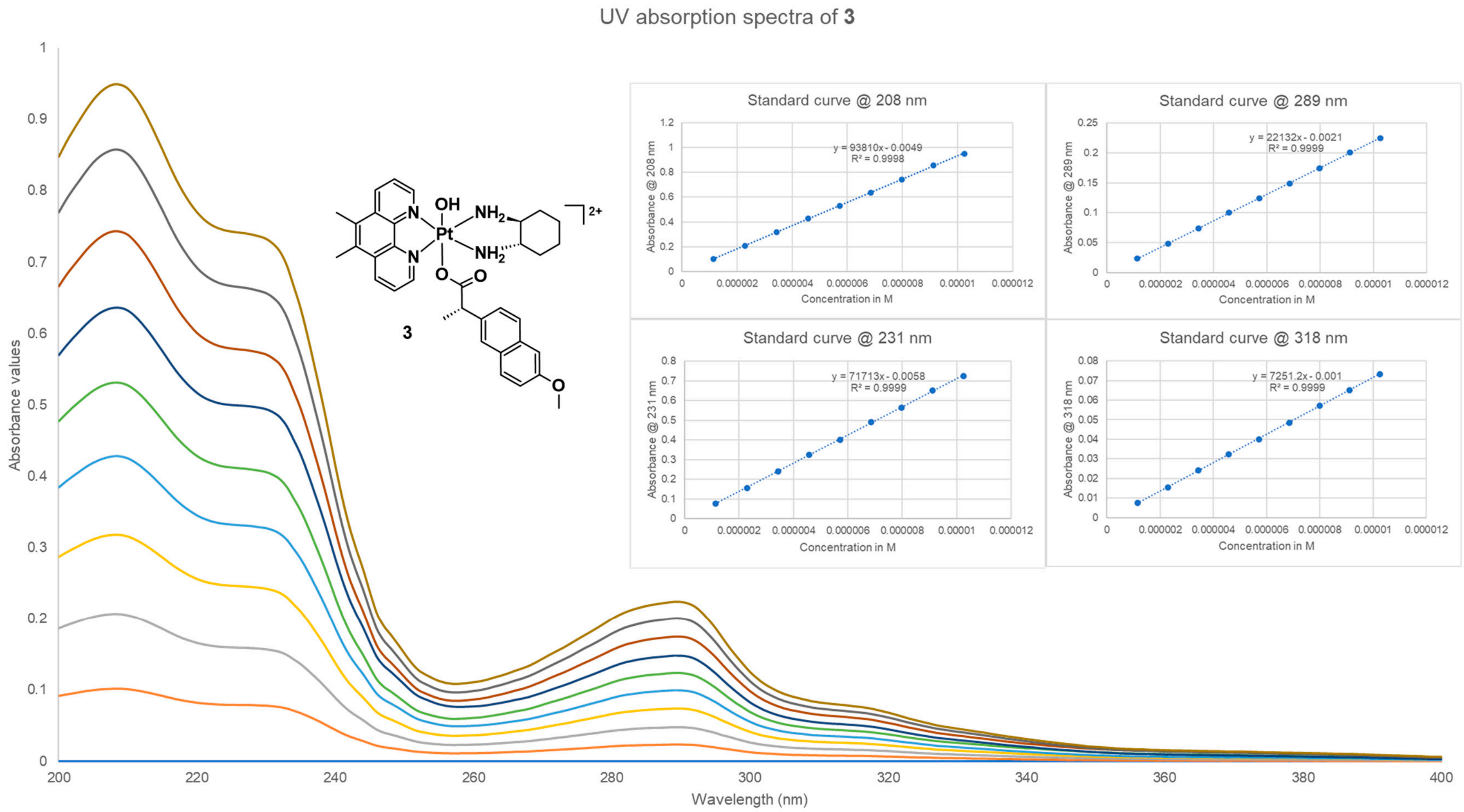

Figure S39: UV spectra of **3** and plot curves at 208, 231, 289 and 318 nm.

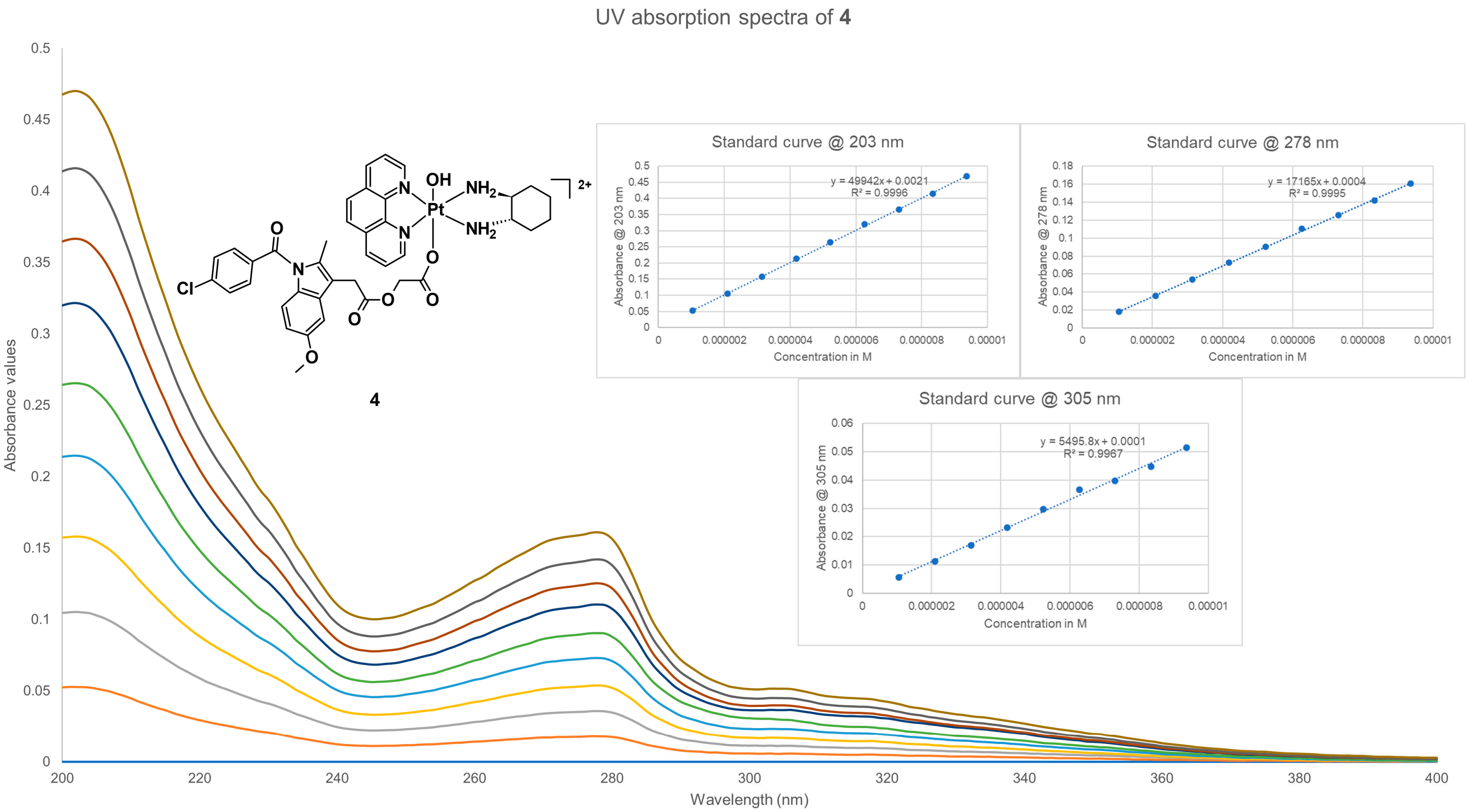

Figure S40: UV spectra of **4** and plot curves at 203, 278 and 305 nm.

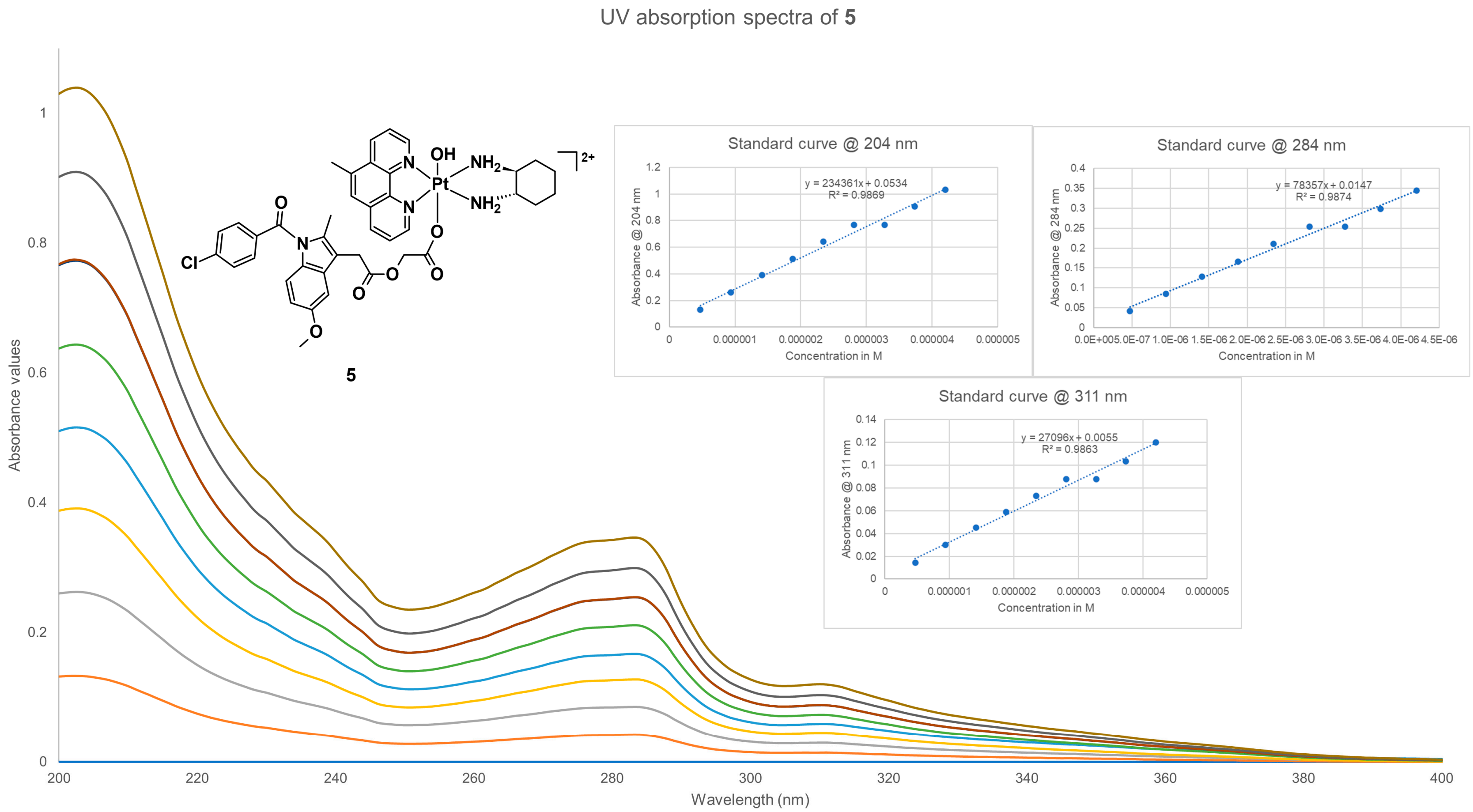

Figure S41: UV spectra of **5** and plot curves at 204, 284 and 311 nm.

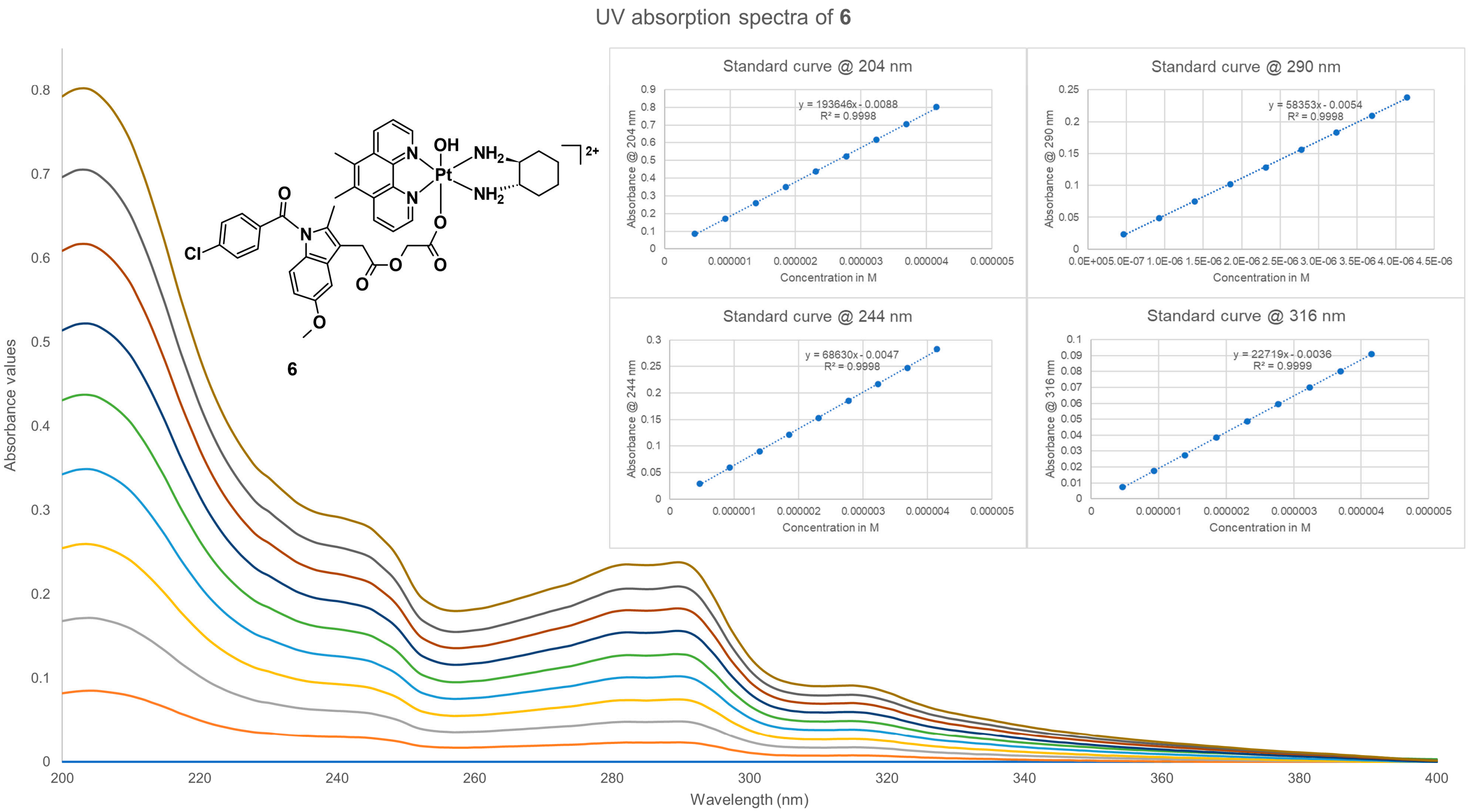

Figure S42: UV spectra of **6** and plot curves at 204, 244, 290 and 316 nm.

CD Spectra of 1–6

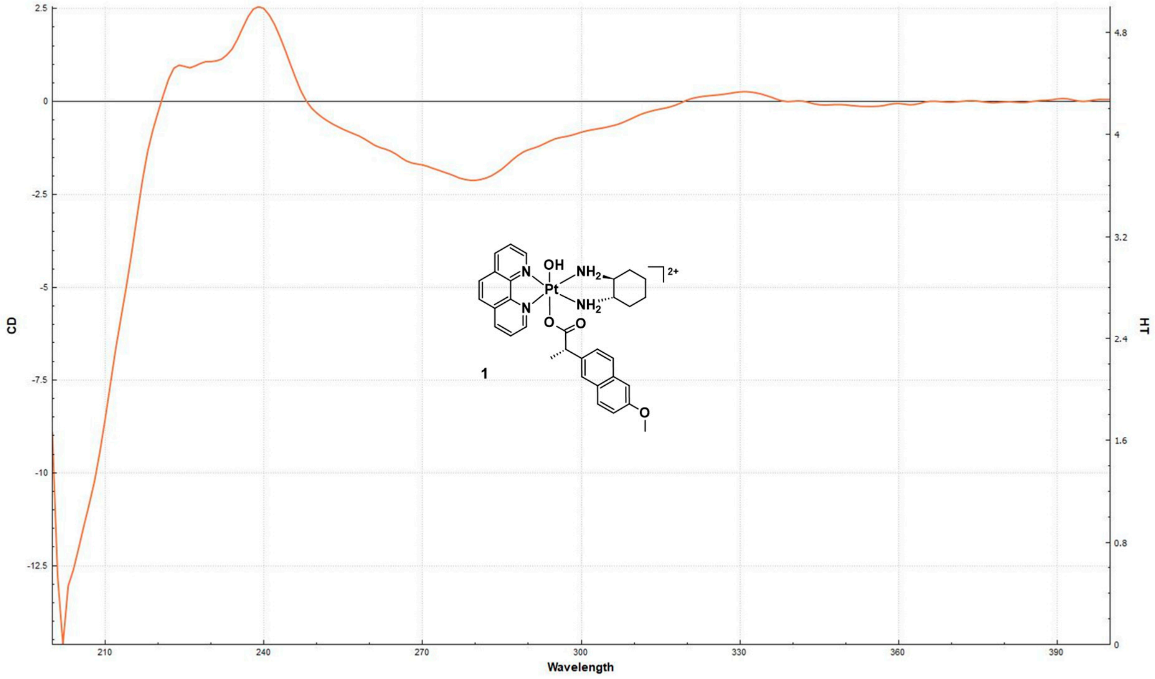

Figure S43: CD spectrum of **1**.

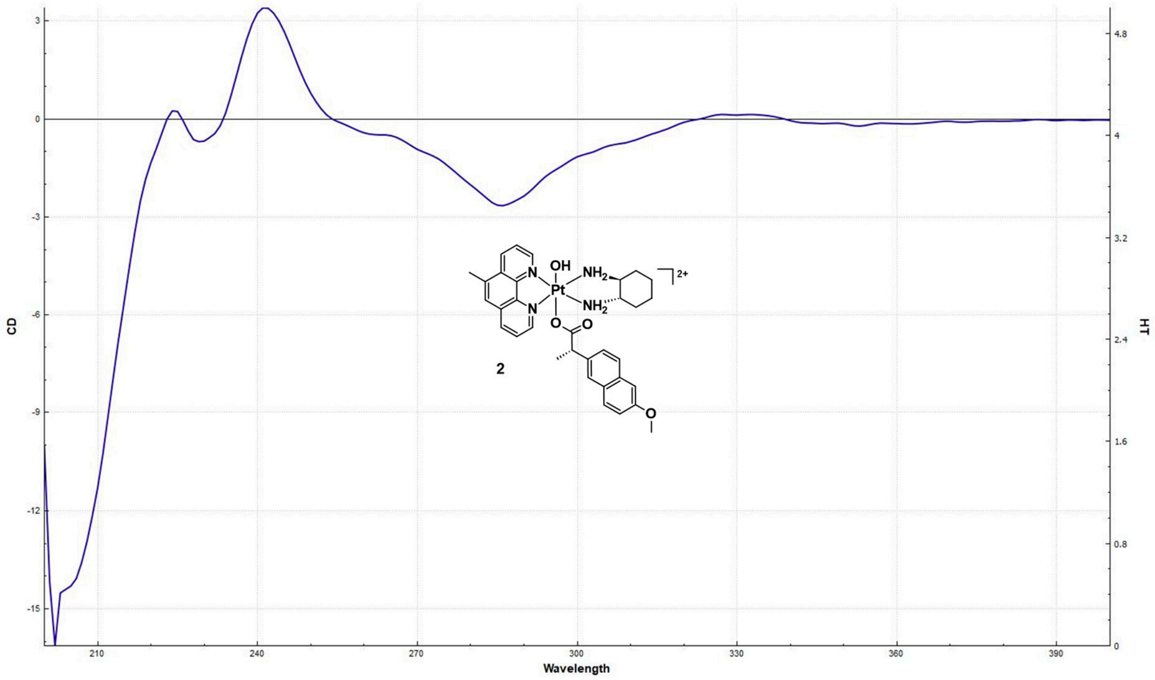

Figure S44: CD spectrum of **2**.

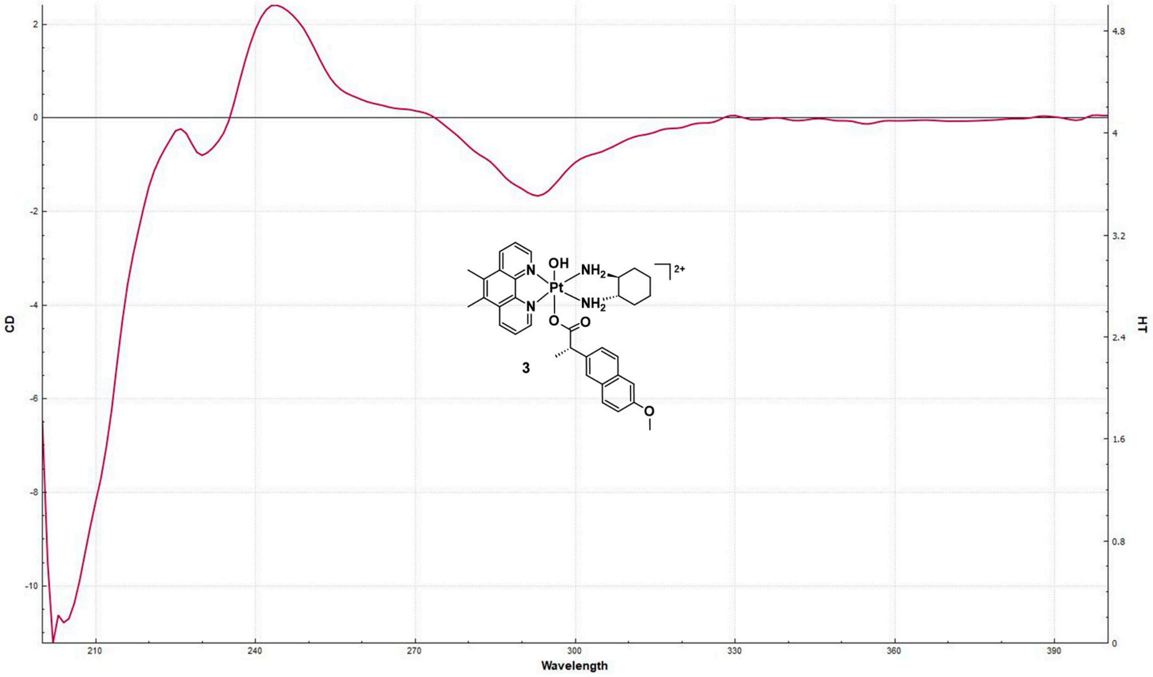

Figure S45: CD spectrum of **3**.

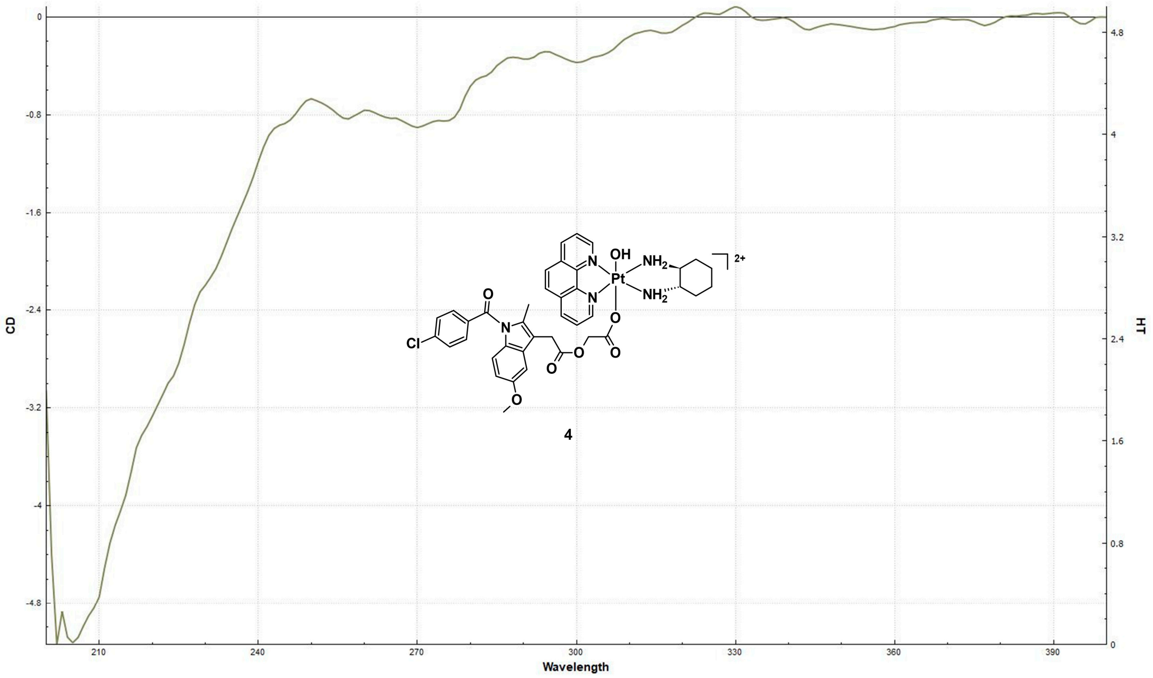

Figure S46: CD spectrum of **4**.

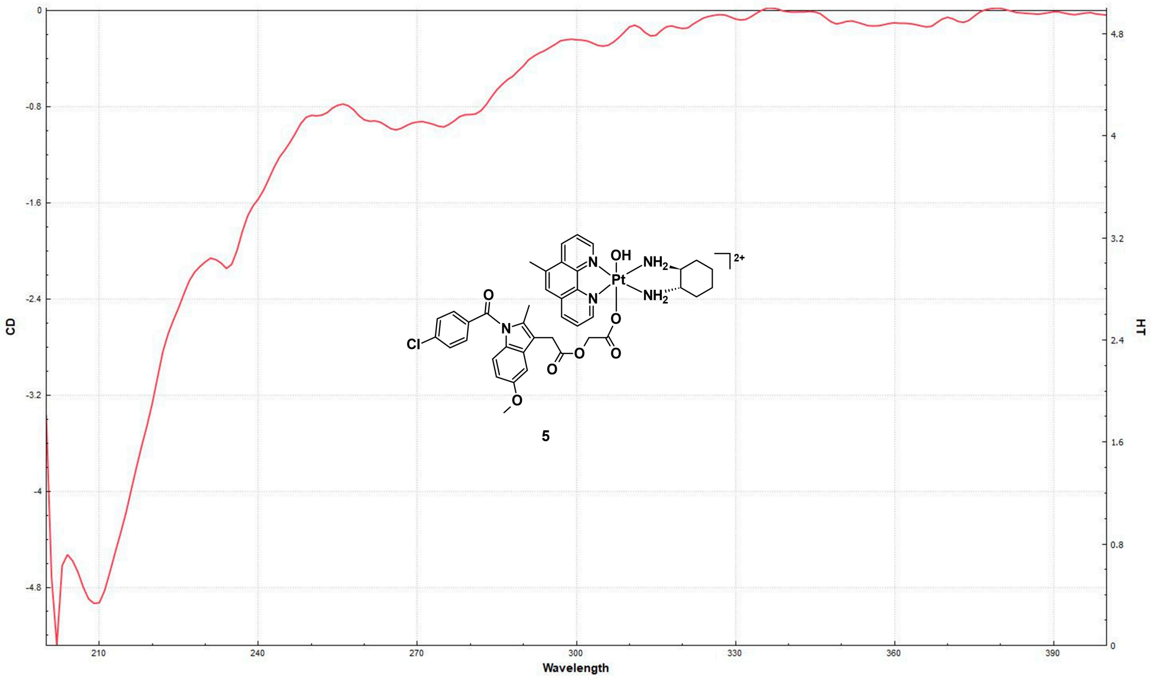

Figure S47: CD spectrum of **5**.

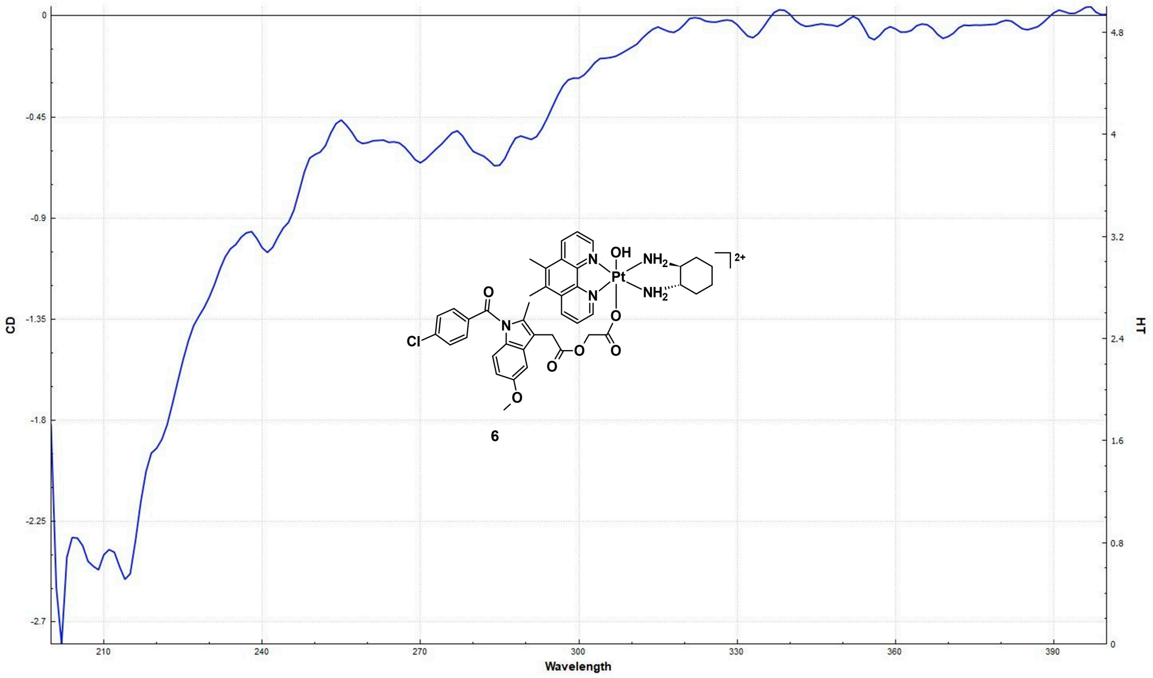

Figure S48: CD spectrum of **6**.

ESI-MS Spectra of 1–6

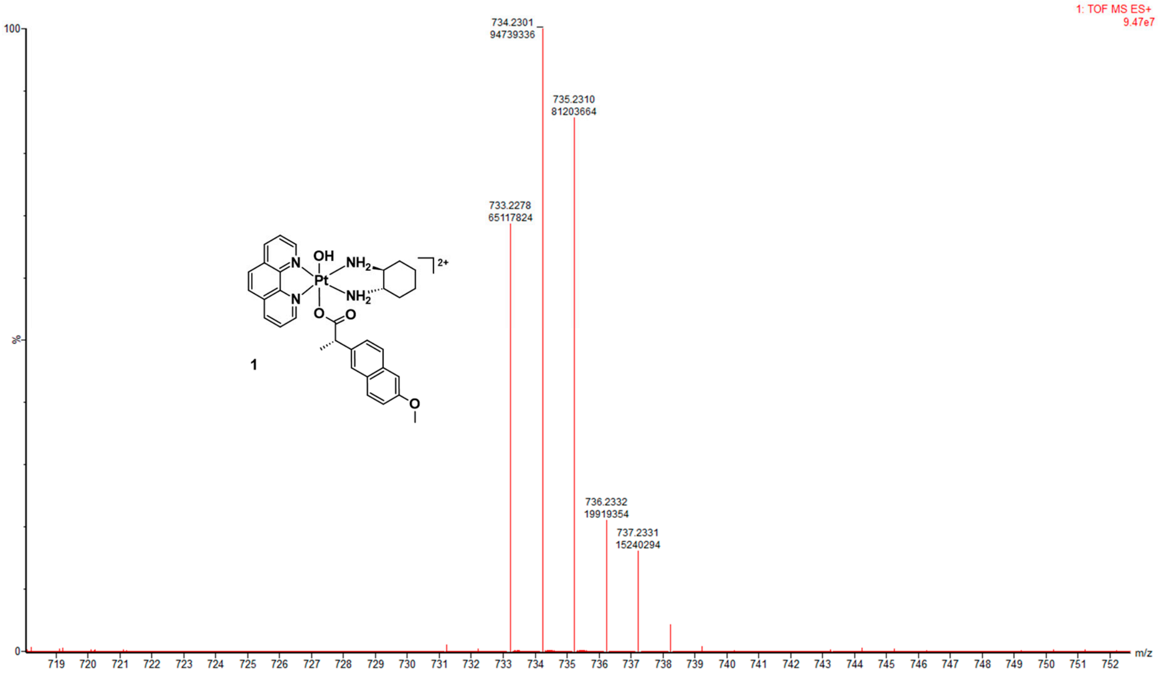

Figure S49: ESI-MS spectrum of **1**.

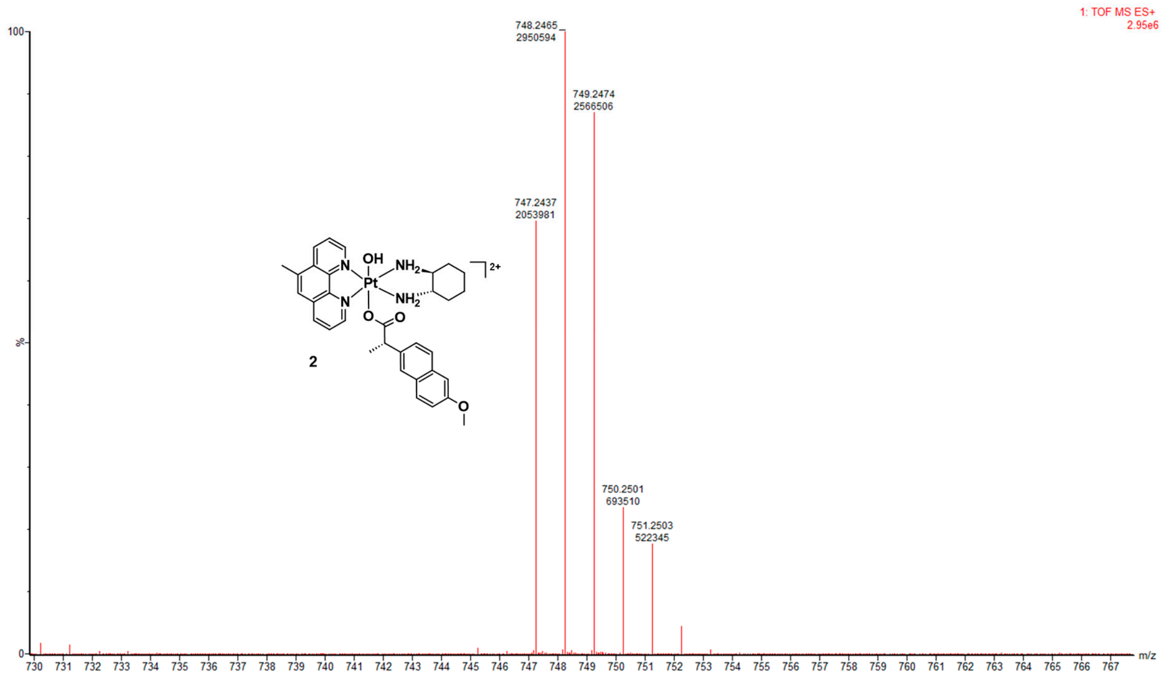

Figure S50: ESI-MS spectrum of **2**.

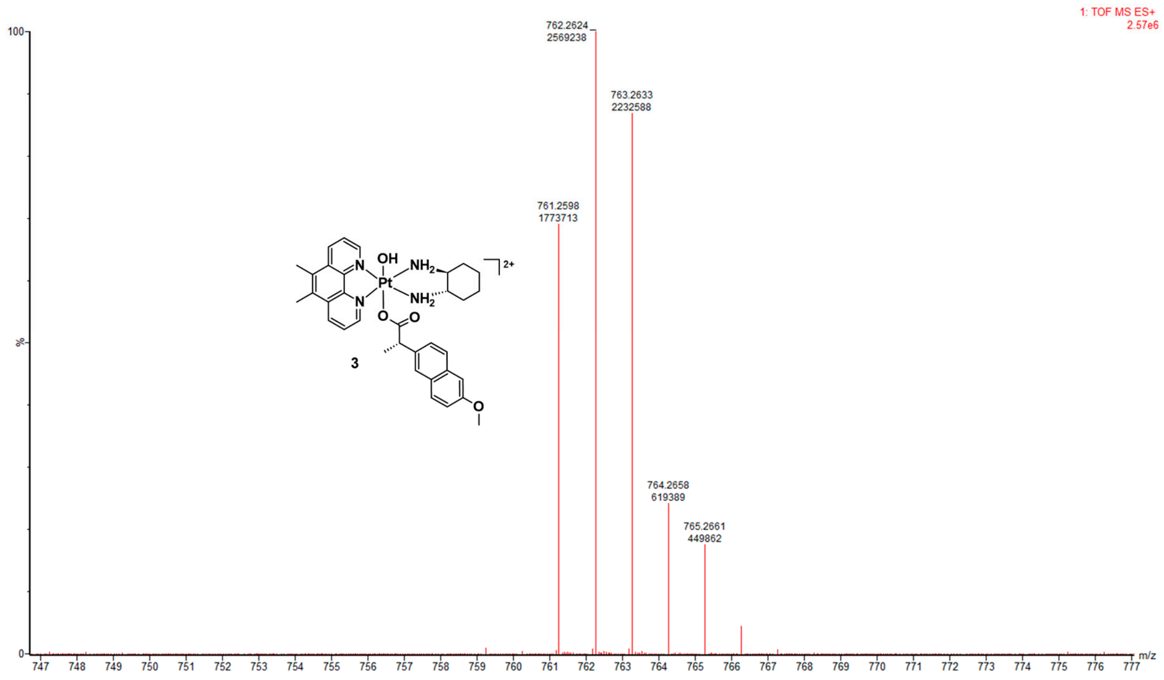

Figure S51: ESI-MS spectrum of **3**.

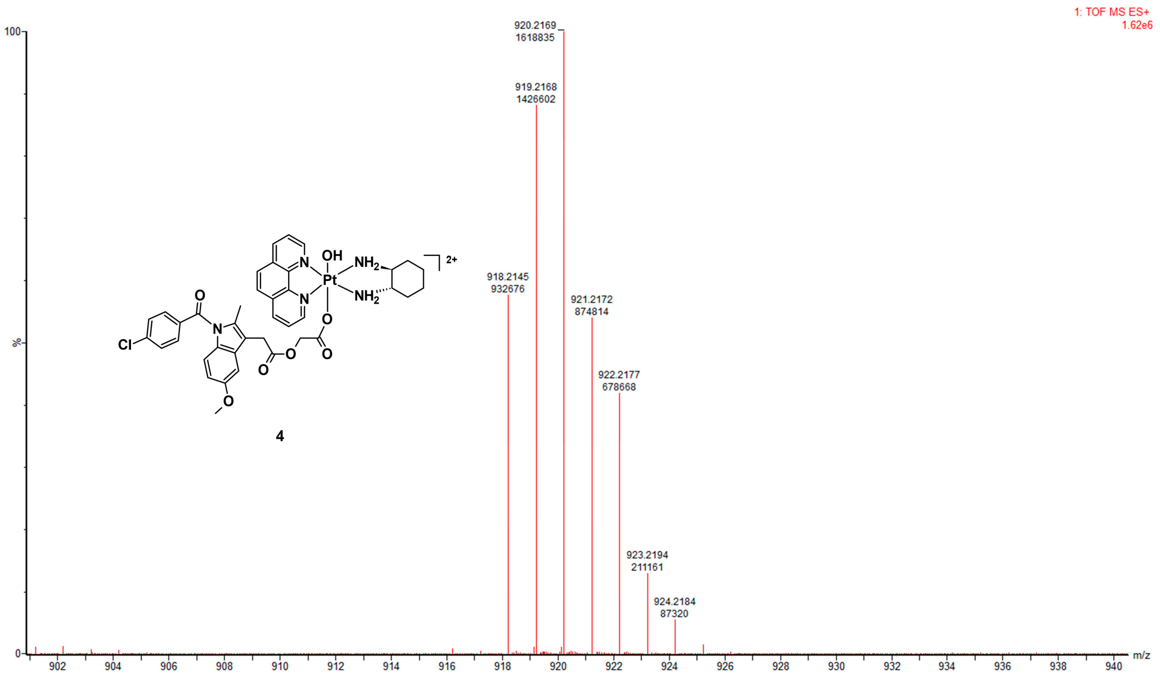

Figure S52: ESI-MS spectrum of **4**.

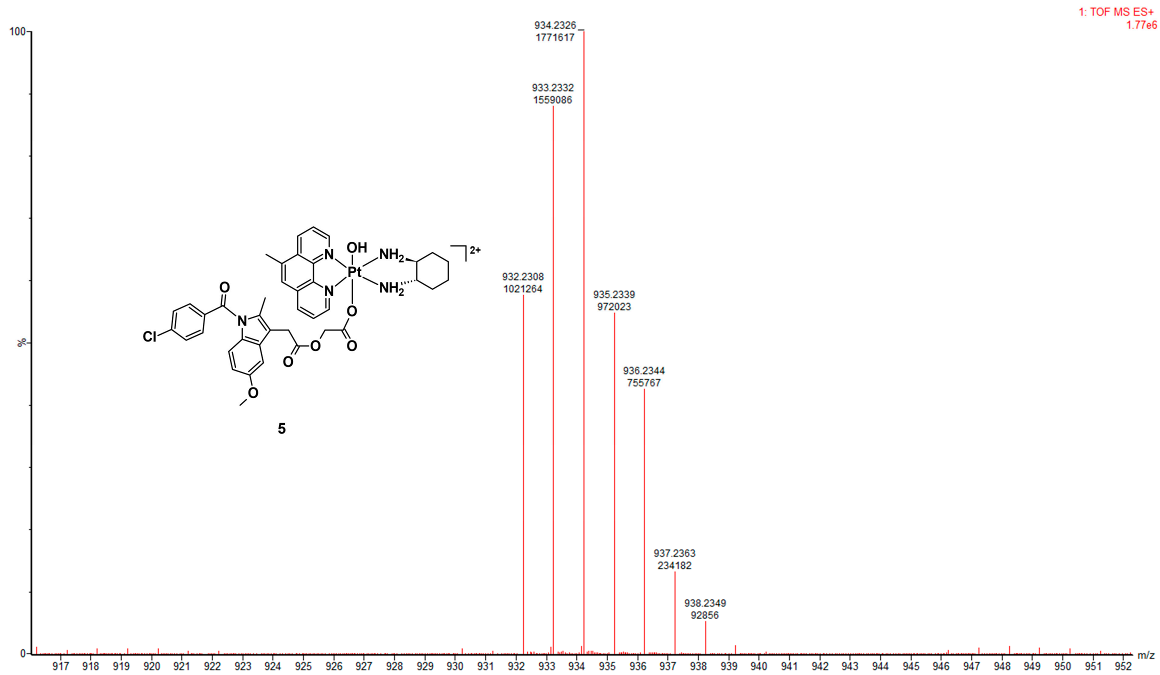

Figure S53: ESI-MS spectrum of **5**.

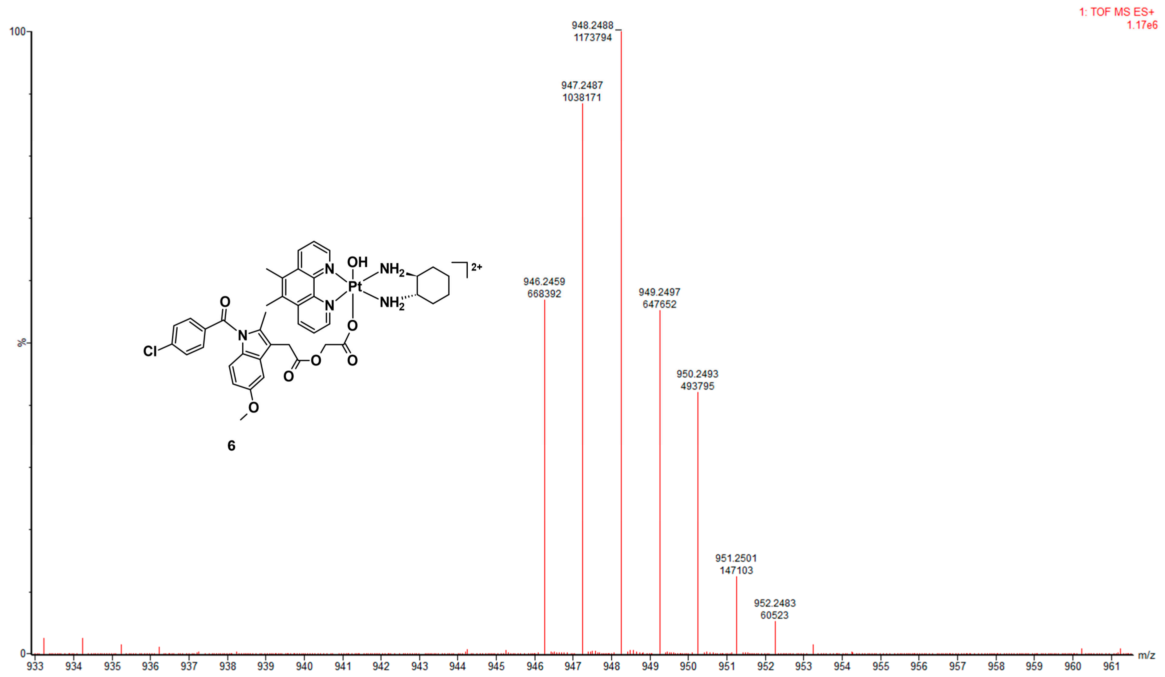

Figure S54: ESI-MS spectrum of **6**.

IR Measurements

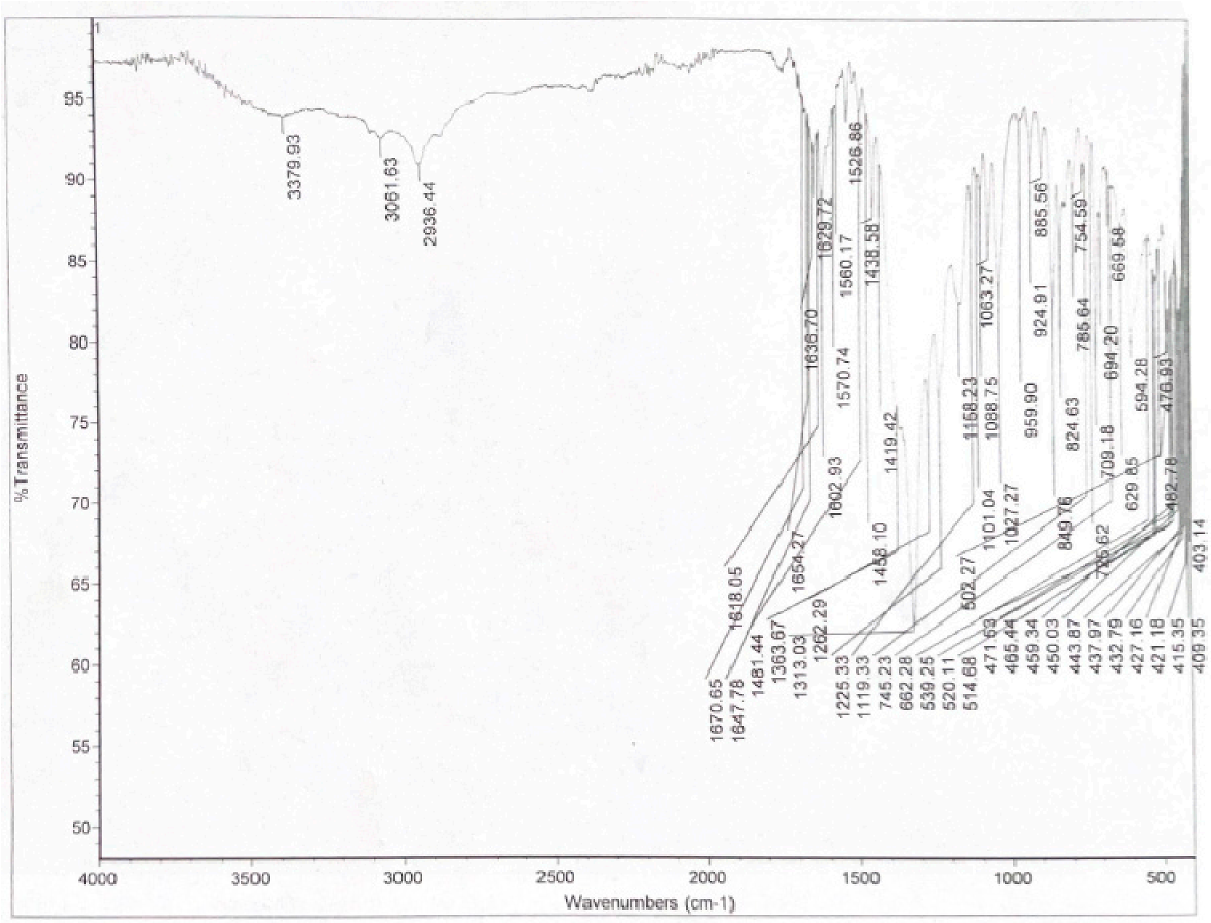

Figure S55: IR spectrum of **1**.

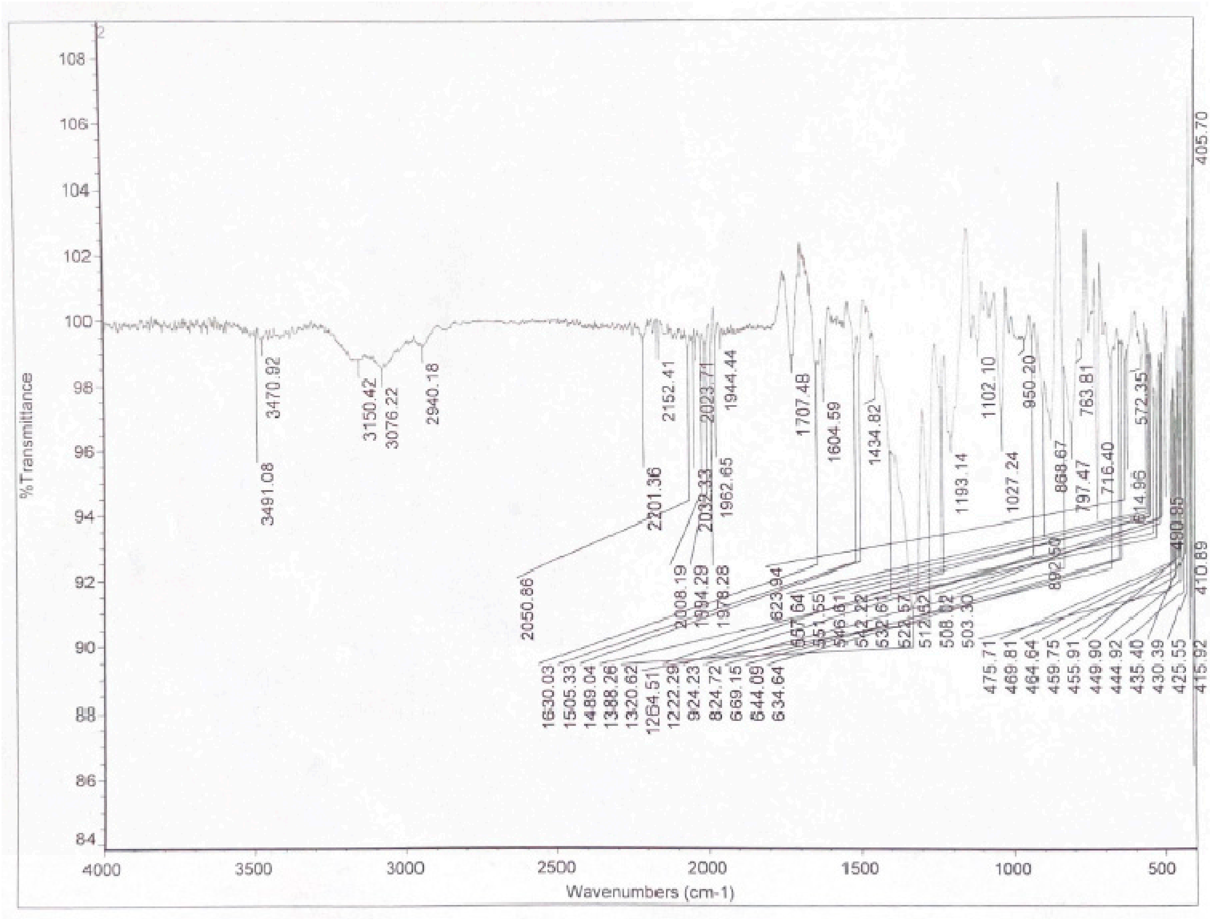

Figure S56: IR spectrum of **2**.

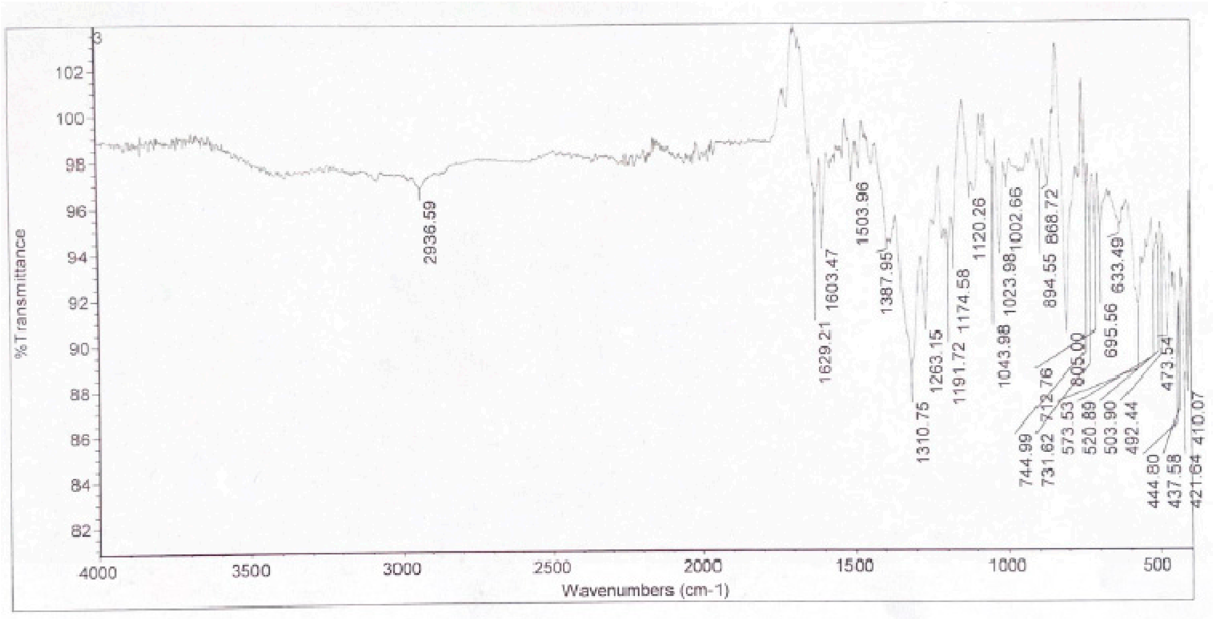

Figure S57: IR spectrum of **3**.

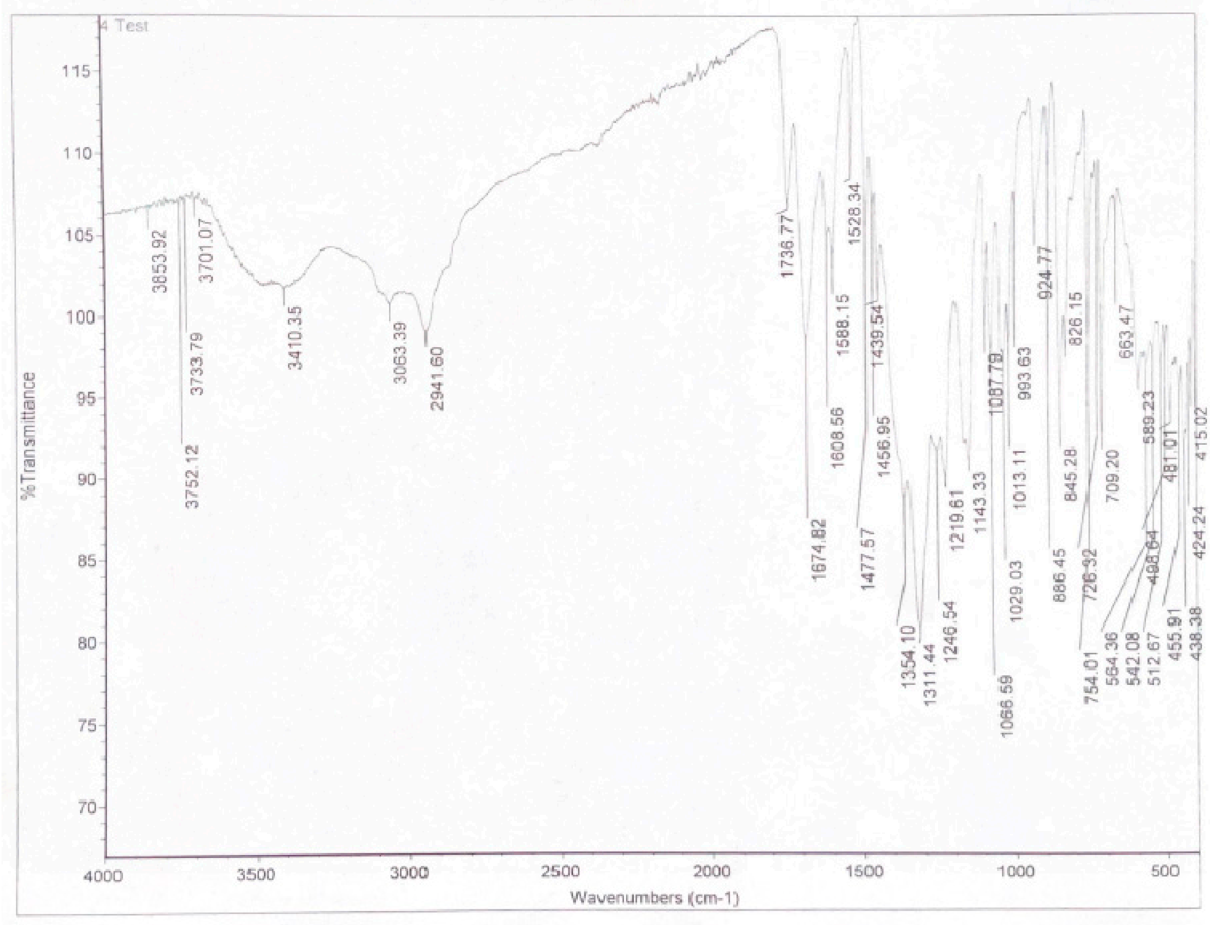

Figure S58: IR spectrum of **4**.

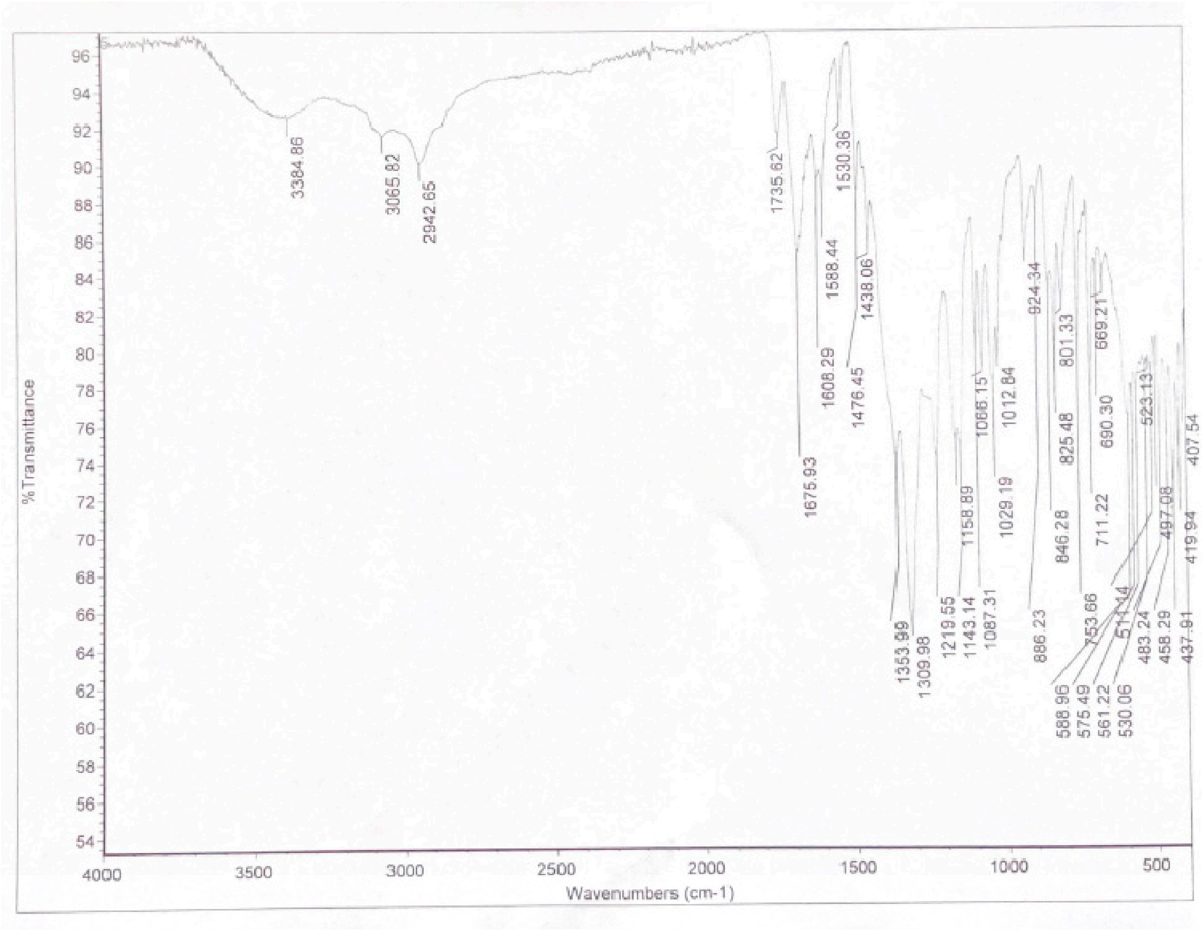

Figure S59: IR spectrum of **5**.

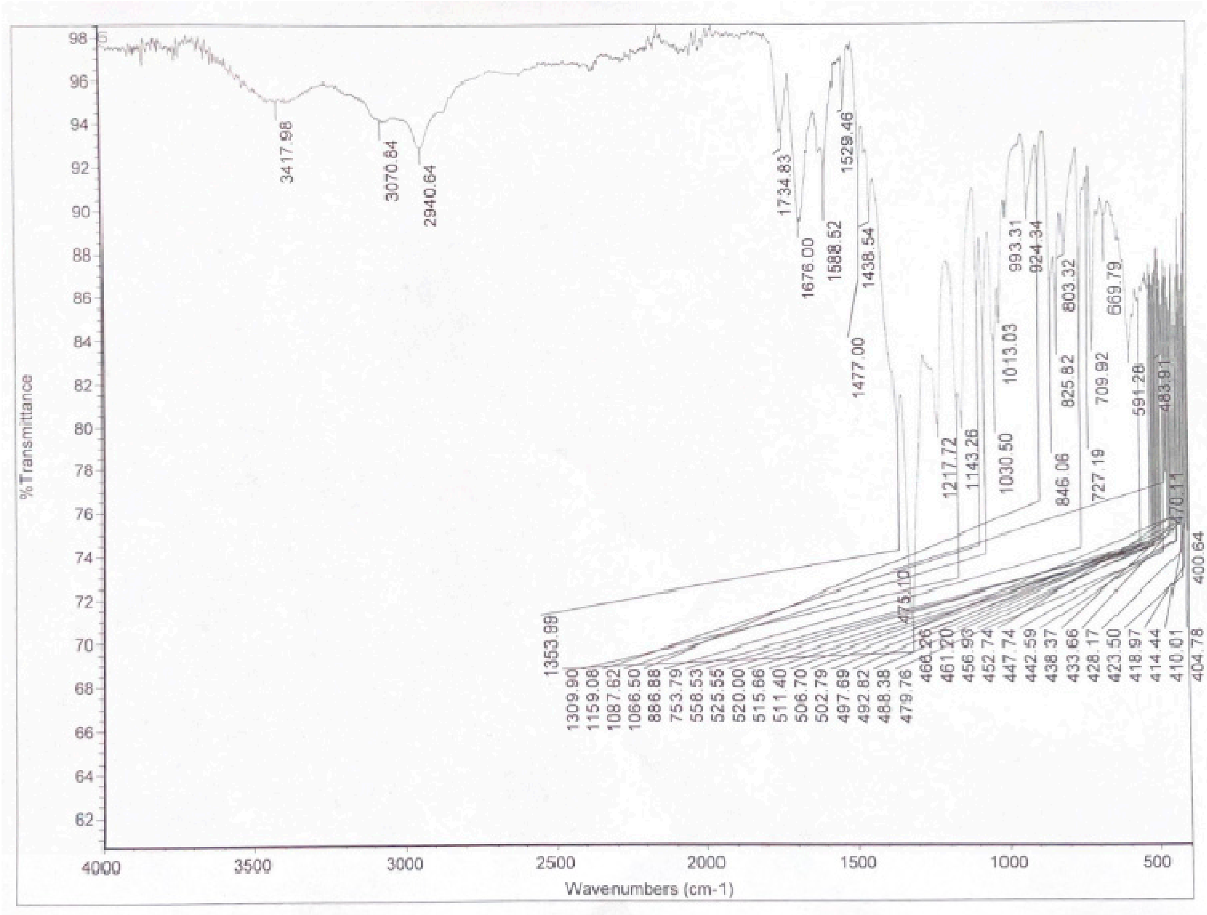

Figure S60: IR spectrum of **6**.

Lipophilicity Measurements

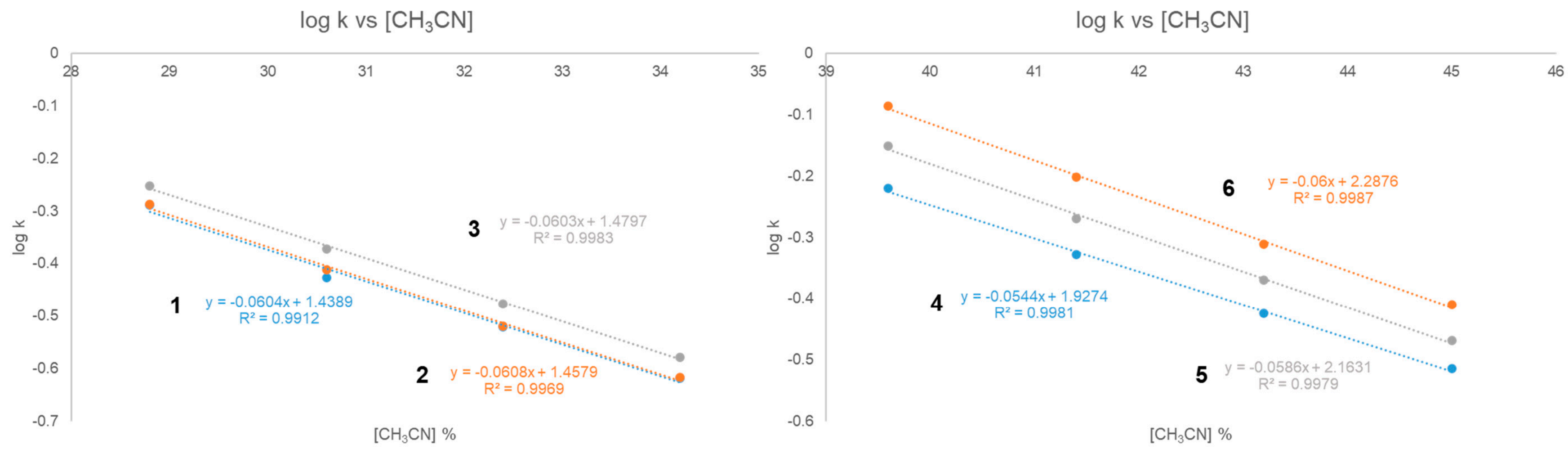

Figure S61: Generated plots of log k versus concentration of the organic solvent, CH_3CN to determine the chromatographic lipophilicity index, log k_w of **1–6**.

Reduction Studies of 1–6 in PBS and Ascorbic Acid

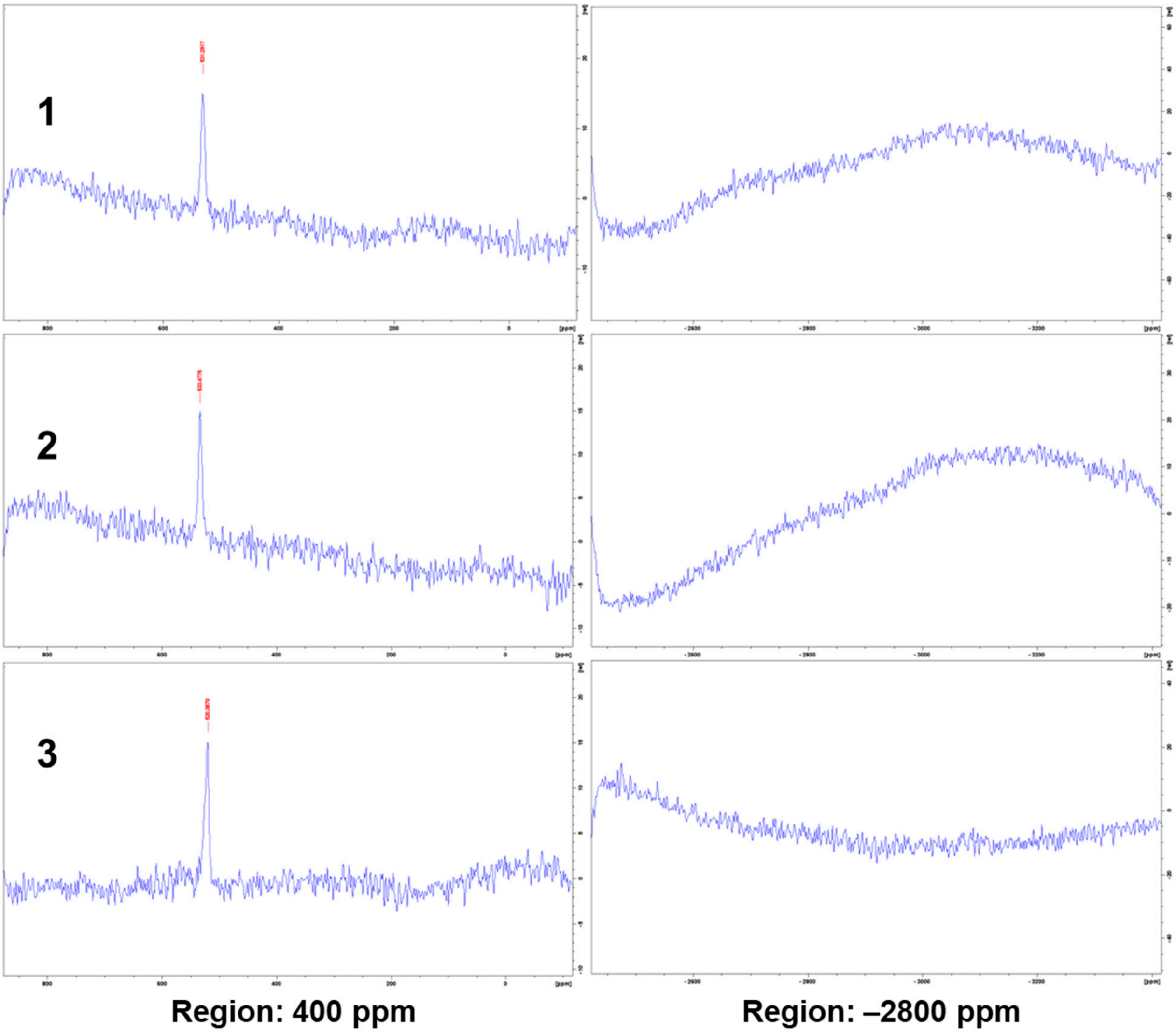

Figure S62: Preliminary 1D-^{195}Pt-NMR spectra of **1**, **2**, and **3** in 10 mM PBS (~7.4 pH) within the regions of 400 and **–**2800 ppm and at 310.15 K.

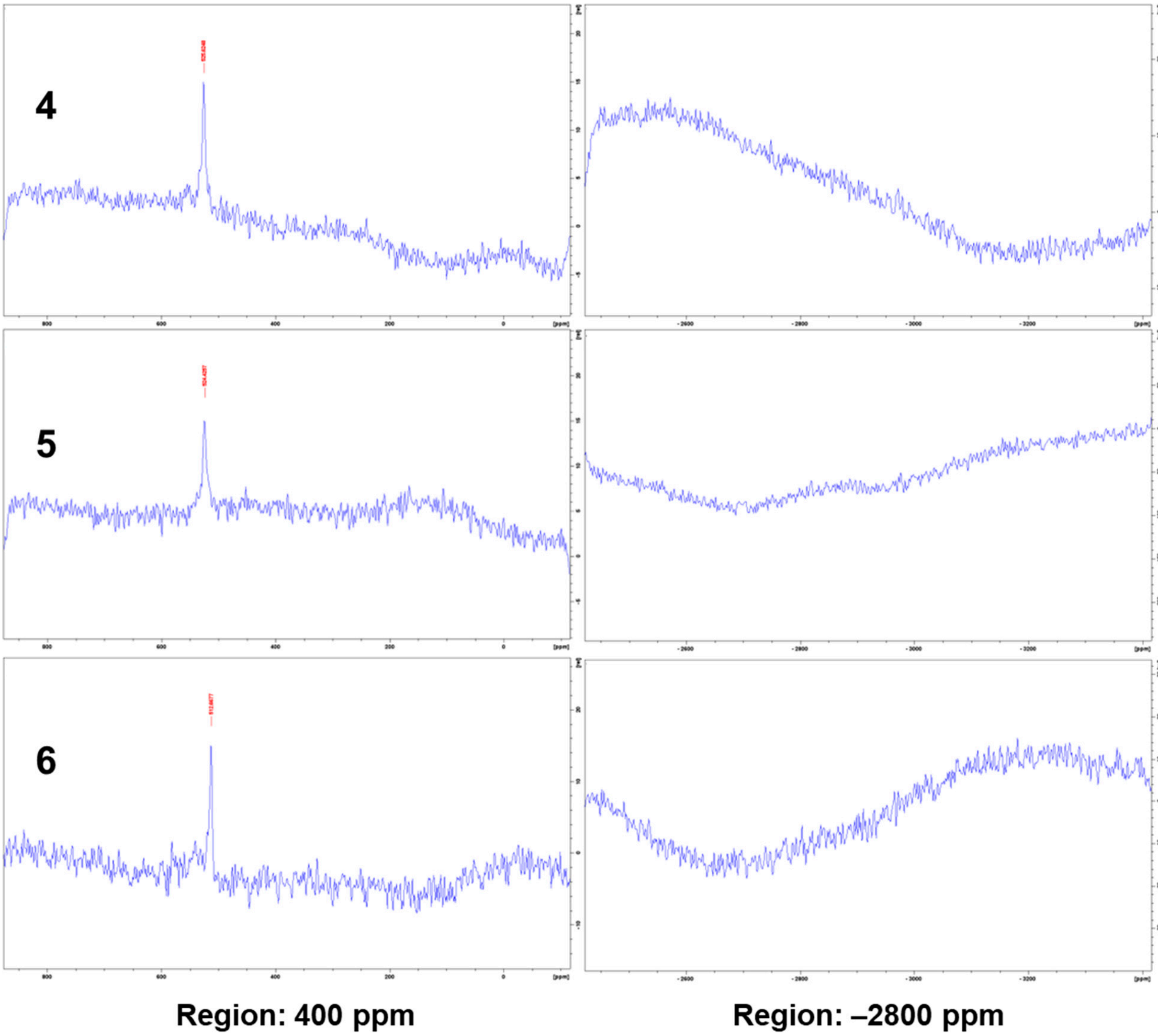

Figure S63: Preliminary 1D-^{195}Pt-NMR spectra of **4**, **5** and **6** in 10 mM PBS (~7.4 pH) within the regions of 400 and **–**2800 ppm and at 310.15 K.

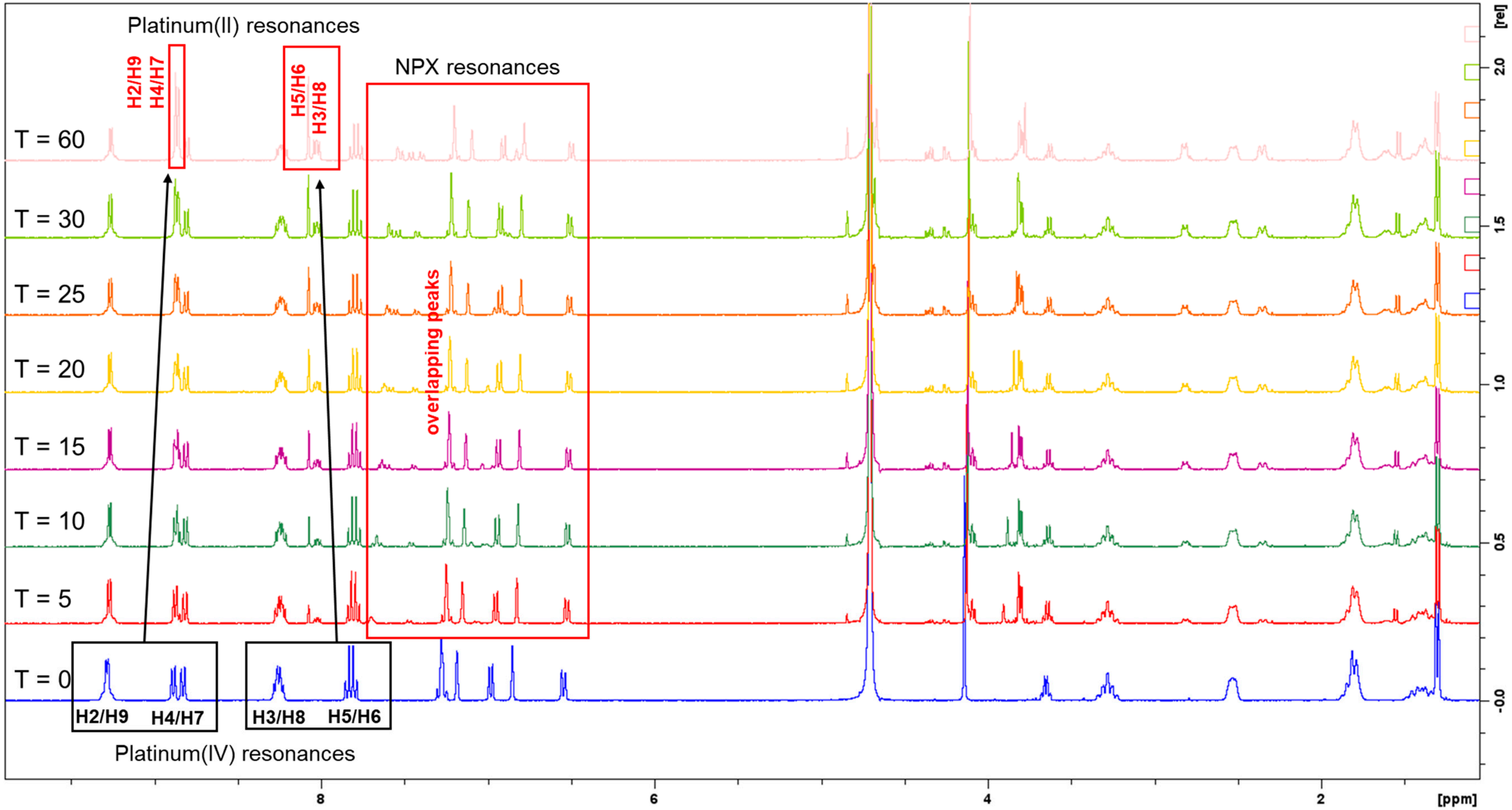

Figure S64: ^{1}H-NMR spectra of **1** with 10 mM PBS (~7.4 pH) and AsA in D_2O at 310.15 K, in different time intervals, highlighting the movement of resonances from the heterocyclic protons and the aromatic protons of the NPX ligand as indicated by the arrows and boxes. **T** represents time in min.

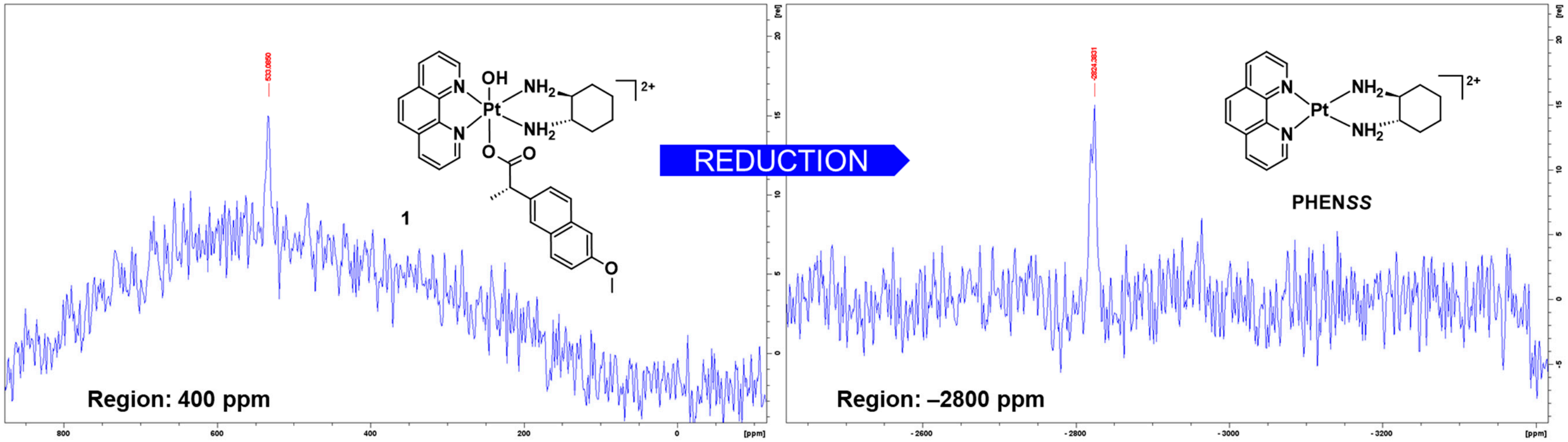

Figure S65: 1D-^{195}Pt-NMR spectra of **1** with 10 mM PBS (~7.4 pH) and AsA in D_2O, within the regions of 400 and **–**2800 ppm at 310.15 K, highlighting its partial reduction after 1 h from the final ^{1}H-NMR experiment. Inset: structures of complex **1** and its corresponding platinum(II) precursor, **PHEN*SS***.

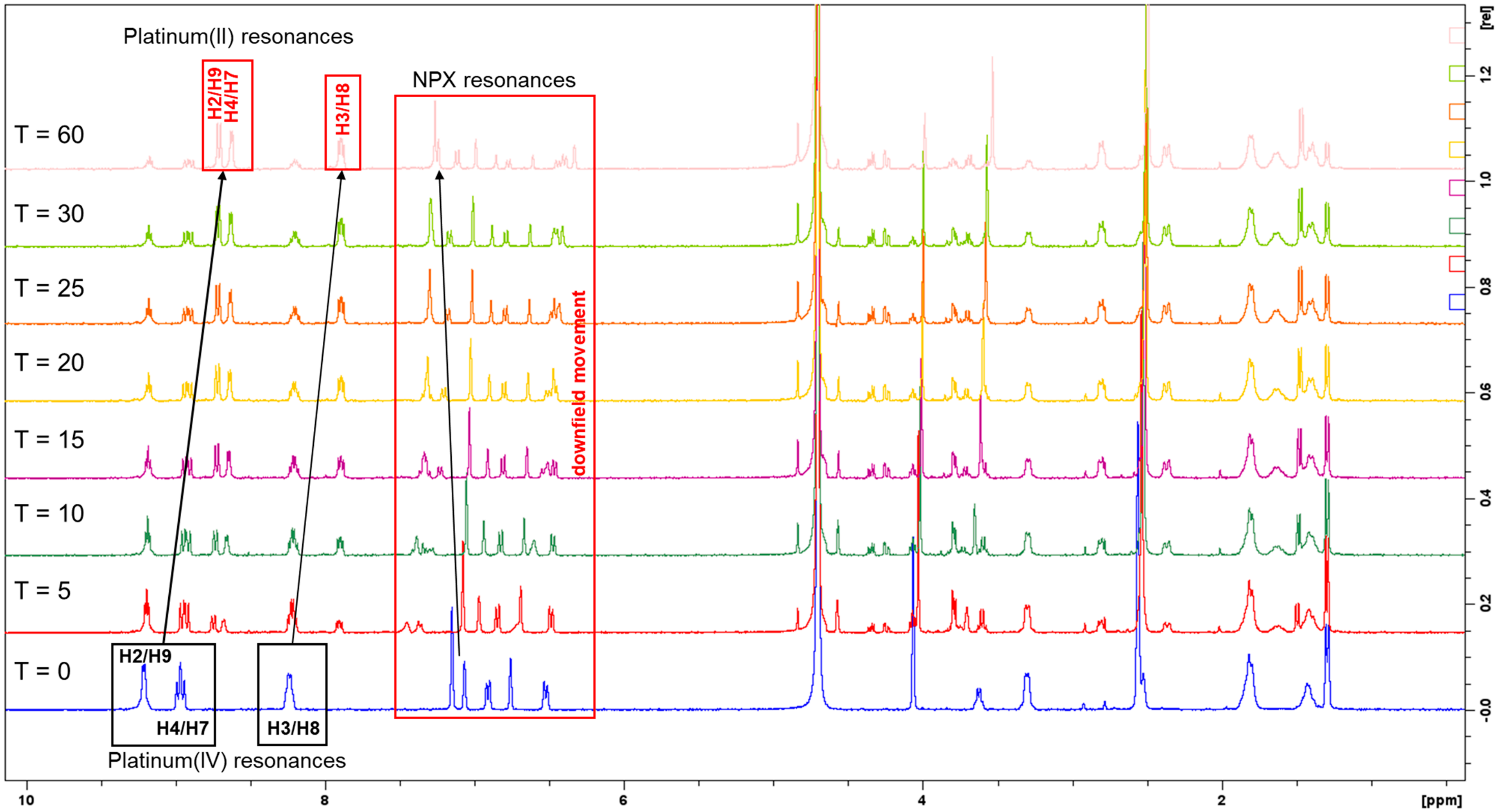

Figure S66: ^{1}H-NMR spectra of **2** with 10 mM PBS (~7.4 pH) and AsA in D_2O at 310.15 K, in different time intervals, highlighting the movement of resonances from the heterocyclic protons and the aromatic protons of the NPX ligand as indicated by the red arrows. **T** represents time in min.

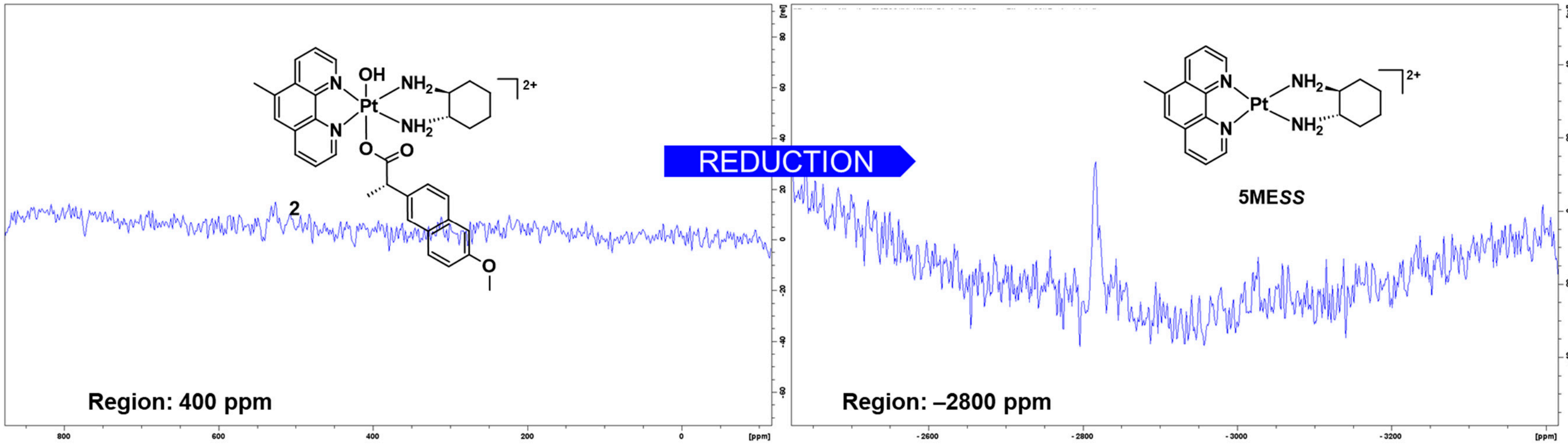

Figure S67: 1D-^{195}Pt-NMR spectra of **2** with 10 mM PBS (~7.4 pH) and AsA in D_2O, within the regions of 400 and **–**2800 ppm at 310.15 K, highlighting its complete reduction after 1 h from the final ^{1}H-NMR experiment. Inset: structures of complex **2** and its corresponding platinum(II) precursor, **5ME*SS***.

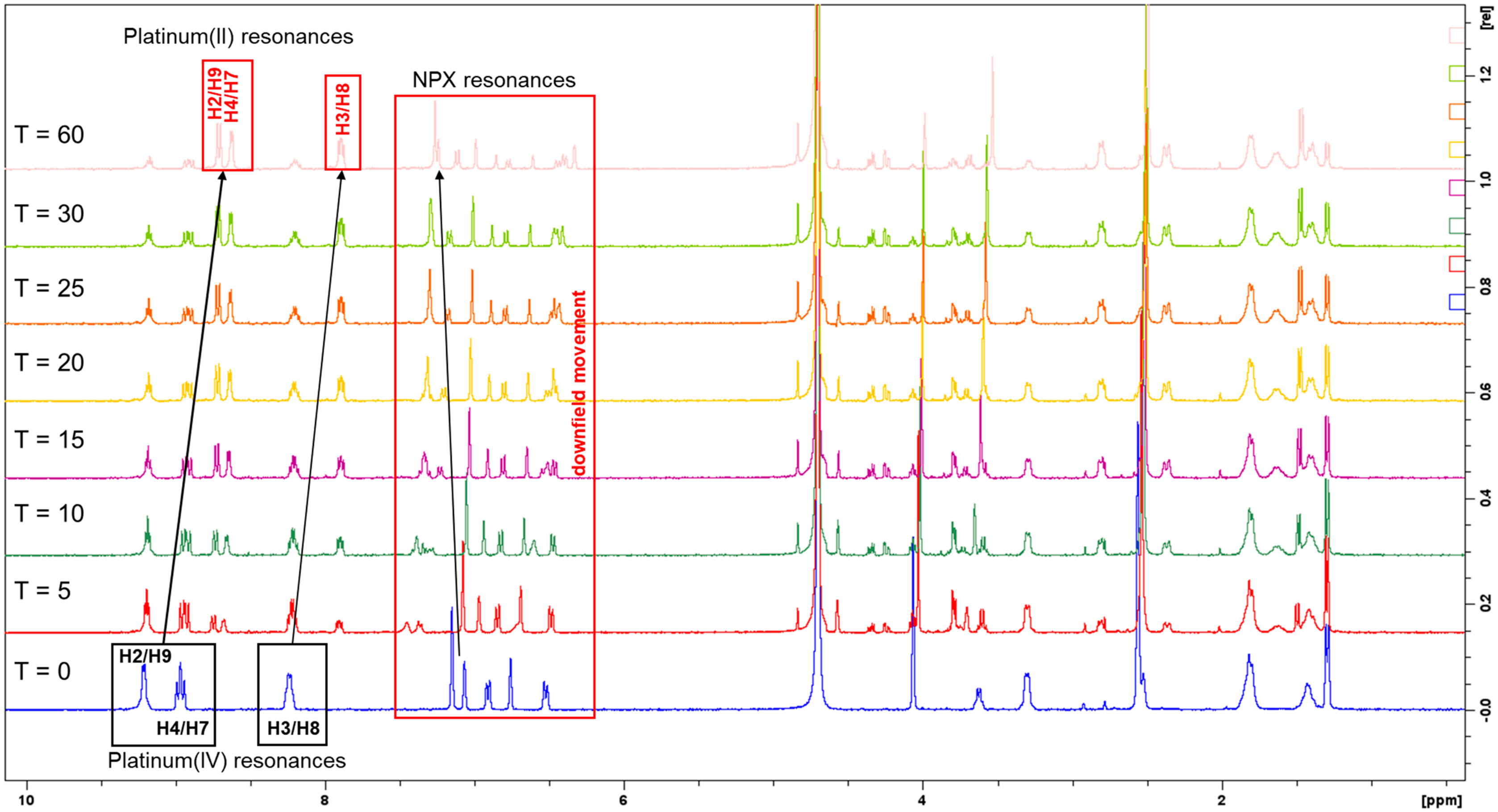

Figure S68: ^{1}H-NMR spectra of **3** with 10 mM PBS (~7.4 pH) and AsA in D_2O at 310.15 K, in different time intervals, highlighting the movement of resonances from the heterocyclic protons and the aromatic protons of the NPX ligand as indicated by the arrows and boxes. **T** represents time in min.

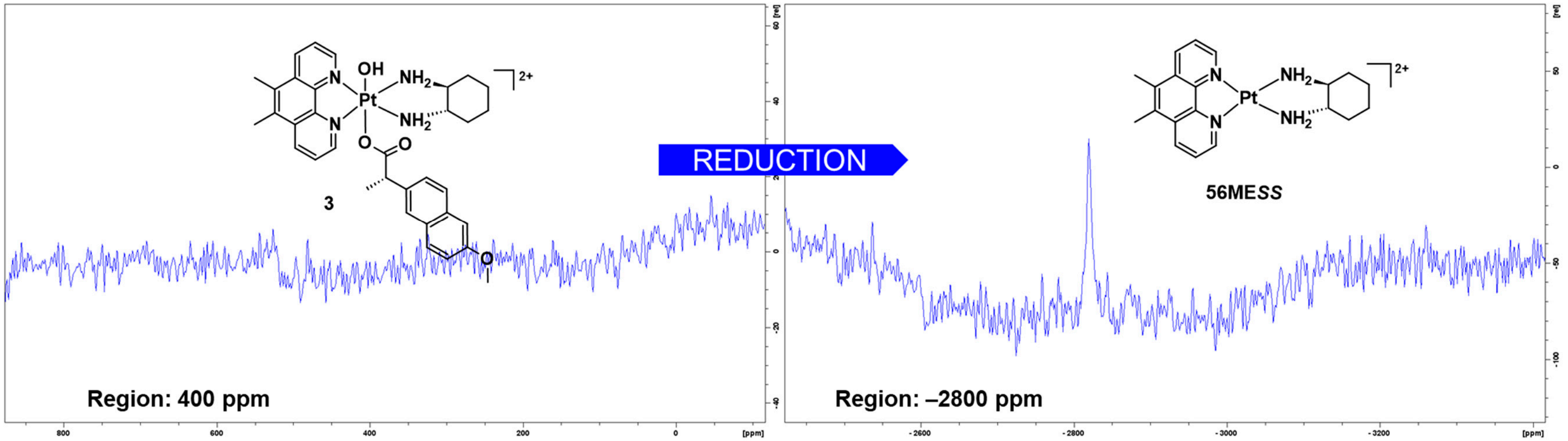

Figure S69: 1D-^{195}Pt-NMR spectra of **3** with 10 mM PBS (~7.4 pH) and AsA in D_2O, within the regions of 400 and **–**2800 ppm at 310.15 K, highlighting its partial reduction after 1 h from the final ^{1}H-NMR experiment. Inset: structures of complex **3** and its corresponding platinum(II) precursor, **56ME*SS***.

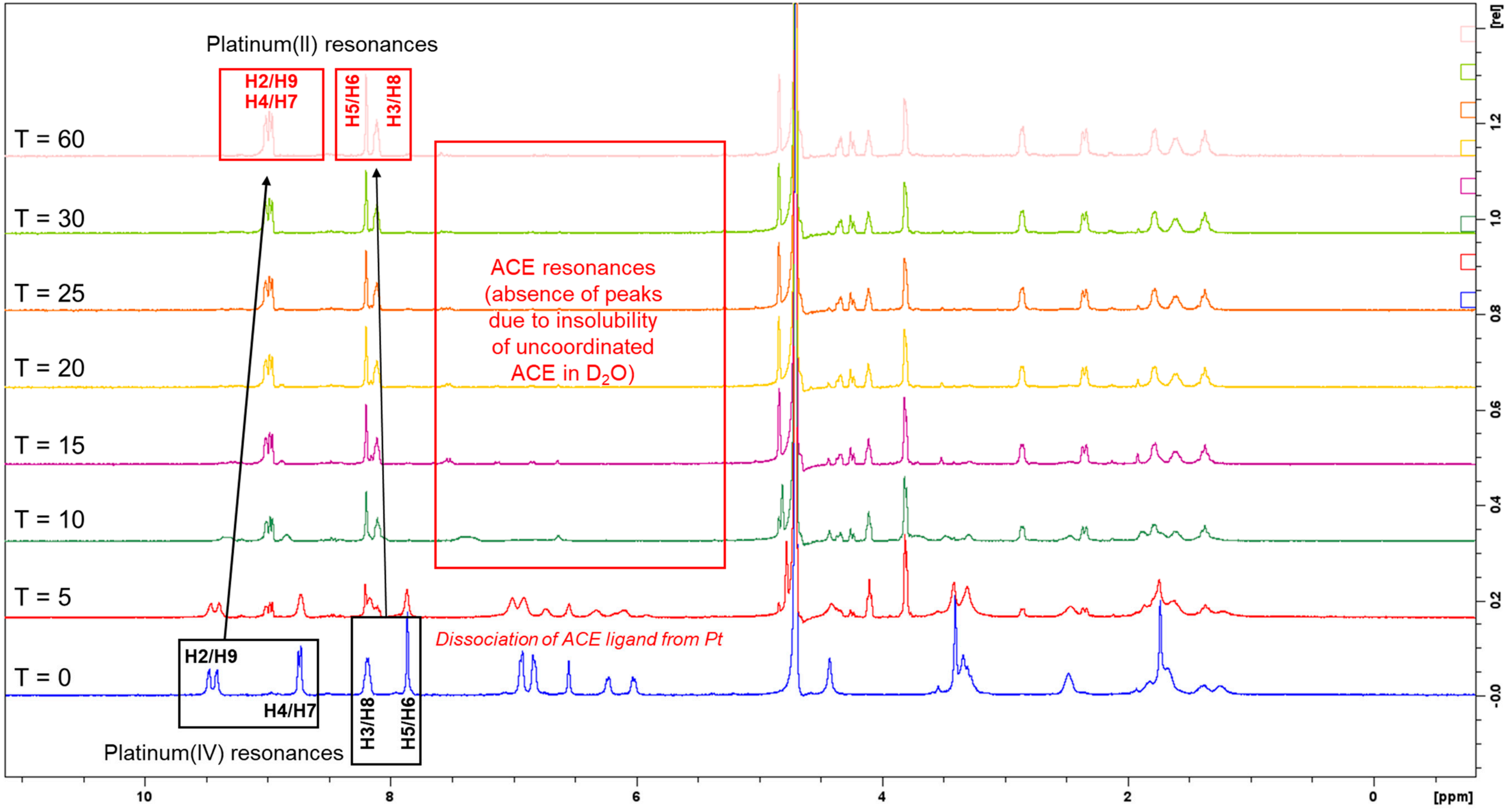

Figure S70: ^{1}H-NMR spectra of **4** with 10 mM PBS (~7.4 pH) and AsA in D_2O at 310.15 K, in different time intervals, highlighting the movement of resonances from the heterocyclic protons and the aromatic protons of the ACE ligand as indicated by the arrows and boxes. **T** represents time in min.

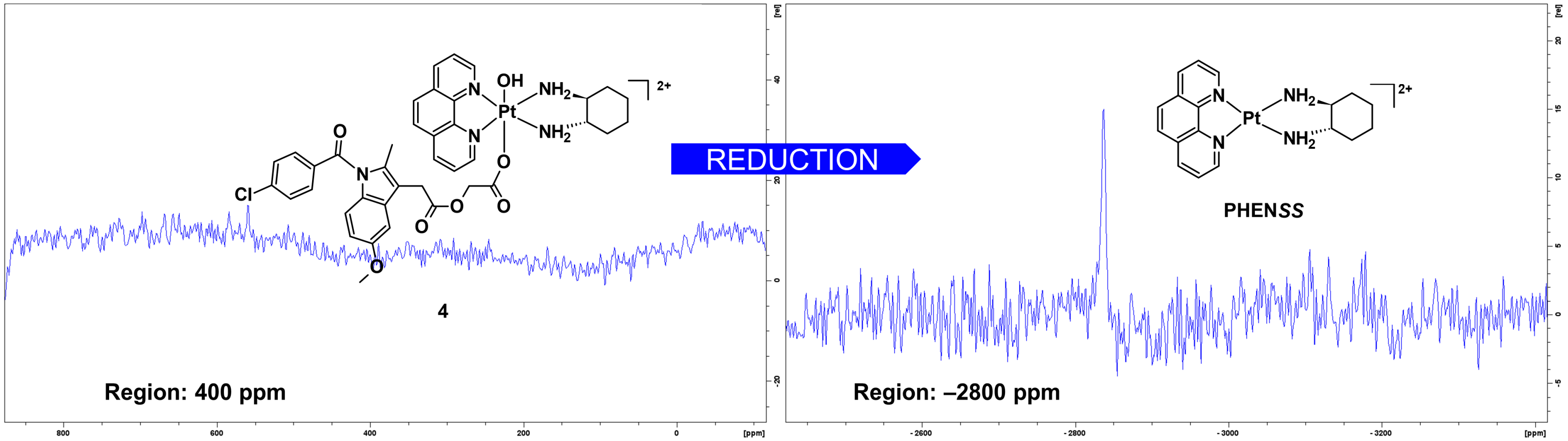

Figure S71: 1D-^{195}Pt-NMR spectra of **4** with 10 mM PBS (~7.4 pH) and AsA in D_2O, within the regions of 400 and **–**2800 ppm at 310.15 K, highlighting its complete reduction after 1 h from the final ^{1}H-NMR experiment. Inset: structures of complex **4** and its corresponding platinum(II) precursor, **PHEN*SS***.

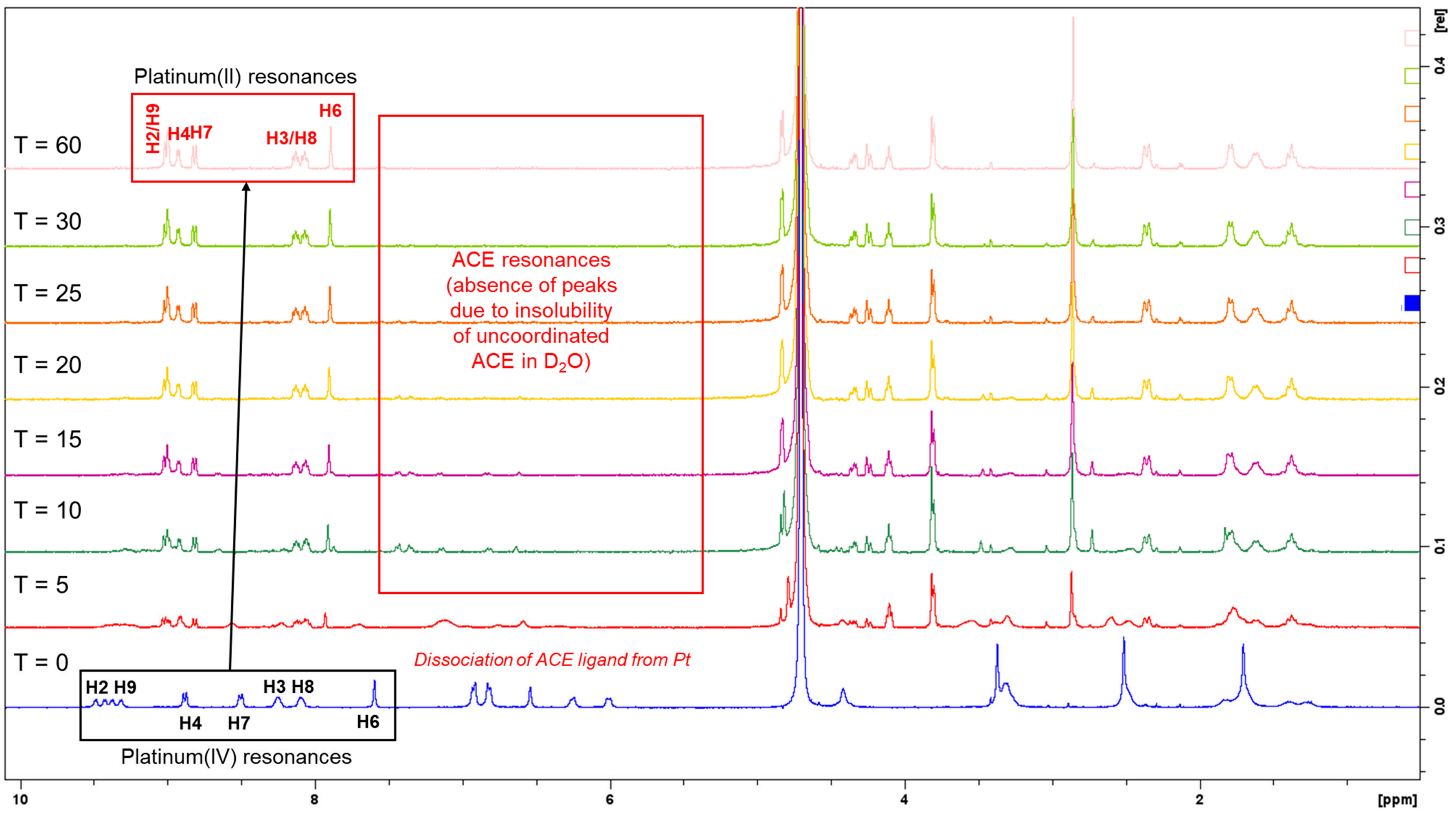

Figure S72: ^{1}H-NMR spectra of **5** with 10 mM PBS (~7.4 pH) and AsA in D_2O at 310.15 K, in different time intervals, highlighting the movement of resonances from the heterocyclic protons and the aromatic protons of the ACE ligand as indicated by the arrows and boxes. **T** represents time in min.

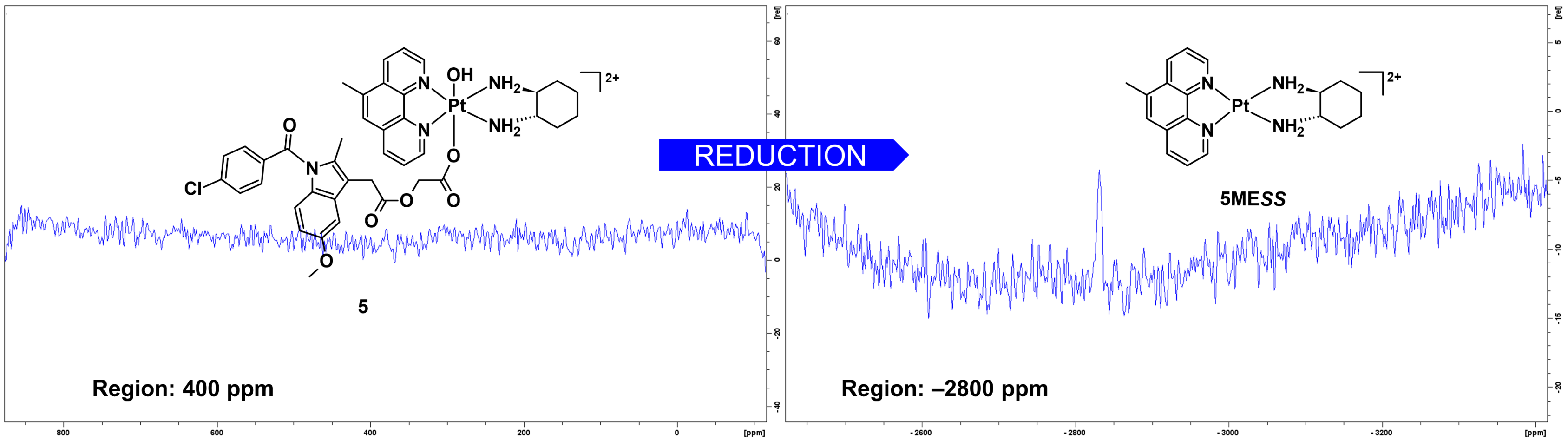

Figure S73: 1D-^{195}Pt-NMR spectra of **5** with 10 mM PBS (~7.4 pH) and AsA in D_2O, within the regions of 400 and **–**2800 ppm at 310.15 K, highlighting its complete reduction after 1 h from the final ^{1}H-NMR experiment. Inset: structures of complex **5** and its corresponding platinum(II) precursor, **5ME*SS***.

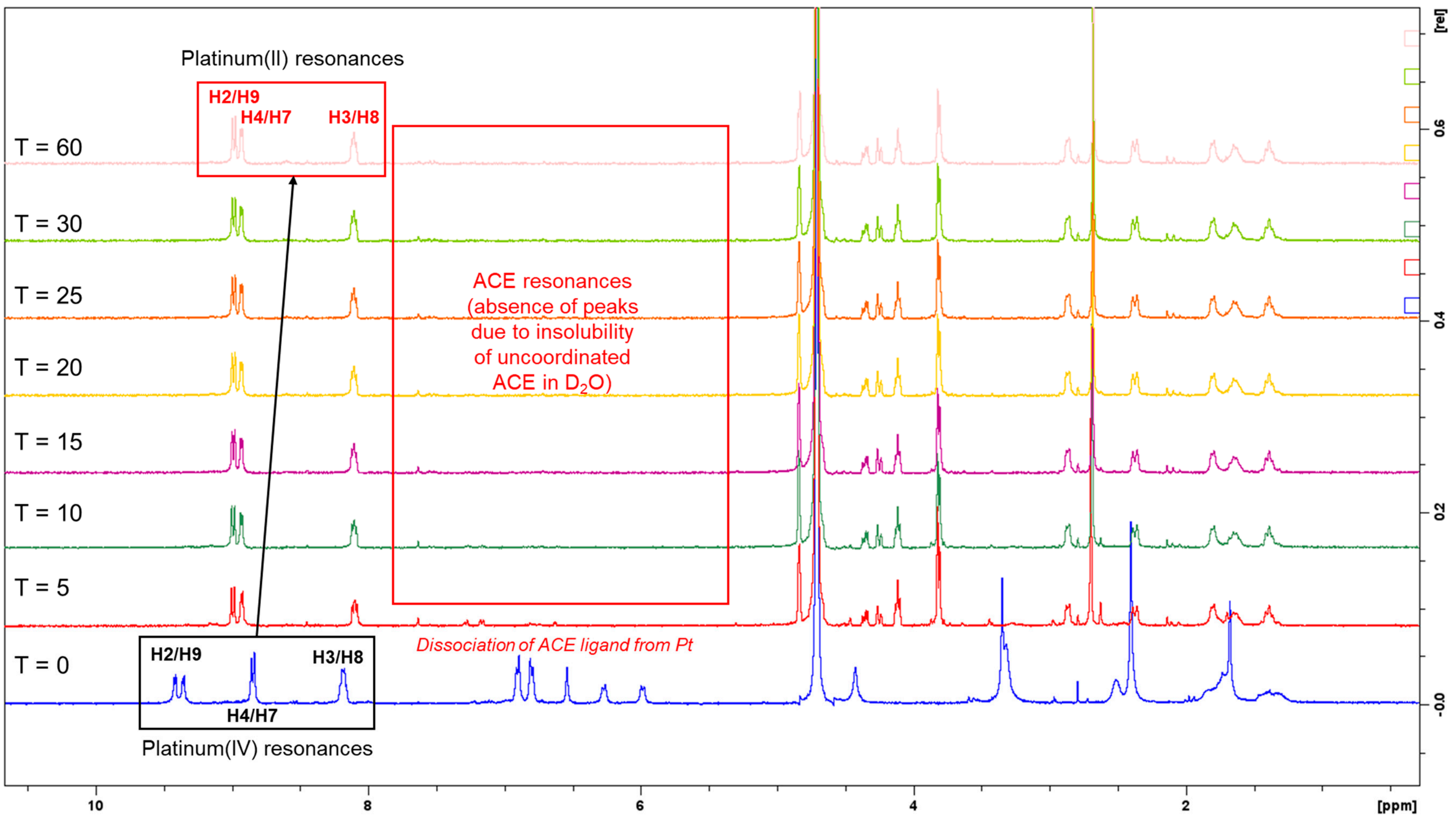

Figure S74: ^{1}H-NMR spectra of **6** with 10 mM PBS (~7.4 pH) and AsA in D_2O at 310.15 K, in different time intervals, highlighting the movement of resonances from the heterocyclic protons and the aromatic protons of the ACE ligand as indicated by the arrows and boxes. **T** represents time in min.

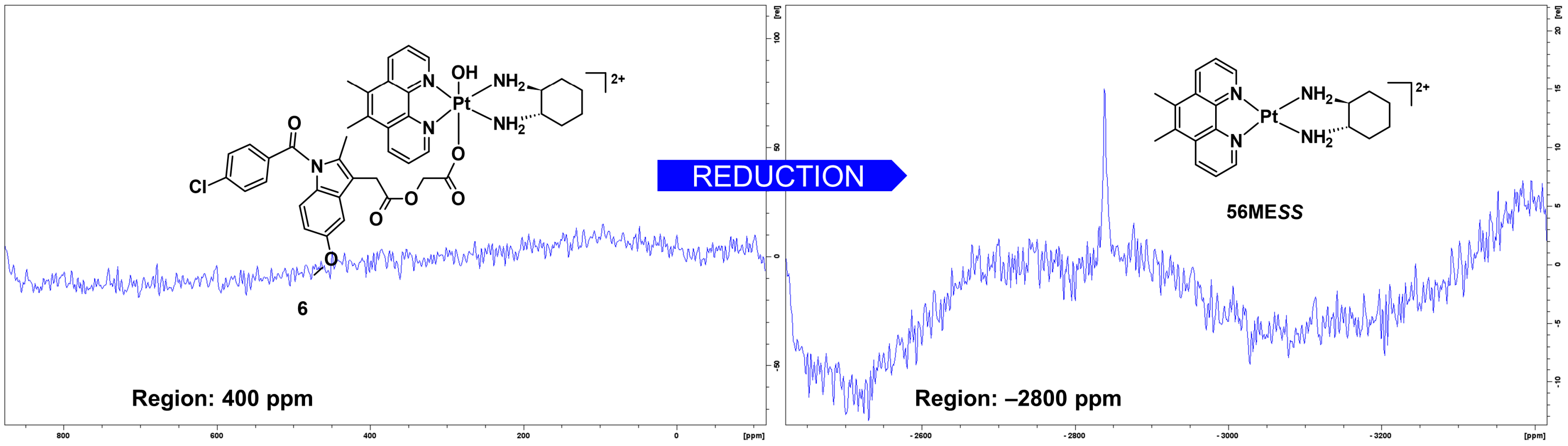

Figure S75: 1D-^{195}Pt-NMR spectra of **6** with 10 mM PBS (~7.4 pH) and AsA in D_2O, within the regions of 400 and **–**2800 ppm at 310.15 K, highlighting its complete reduction after 1 h from the final ^{1}H-NMR experiment. Inset: structures of complex **6** and its corresponding platinum(II) precursor, **56ME*SS***.

Stability Studies of 1–6 in PBS

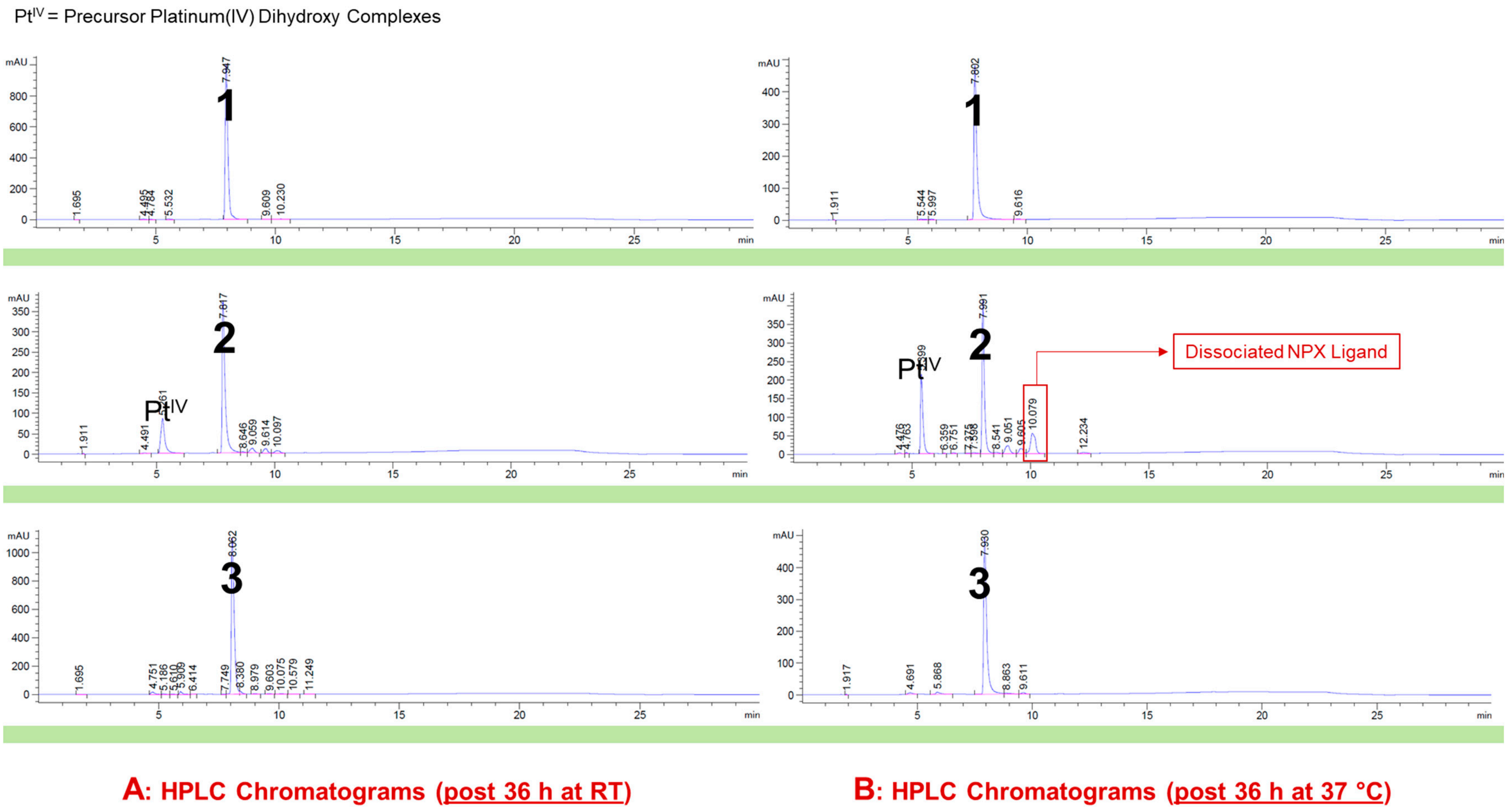

Figure S76: Combined HPLC chromatograms acquired for **1–3** incubated with PBS (~7.4 pH) at room temperature and at 37 °C after 36 h.

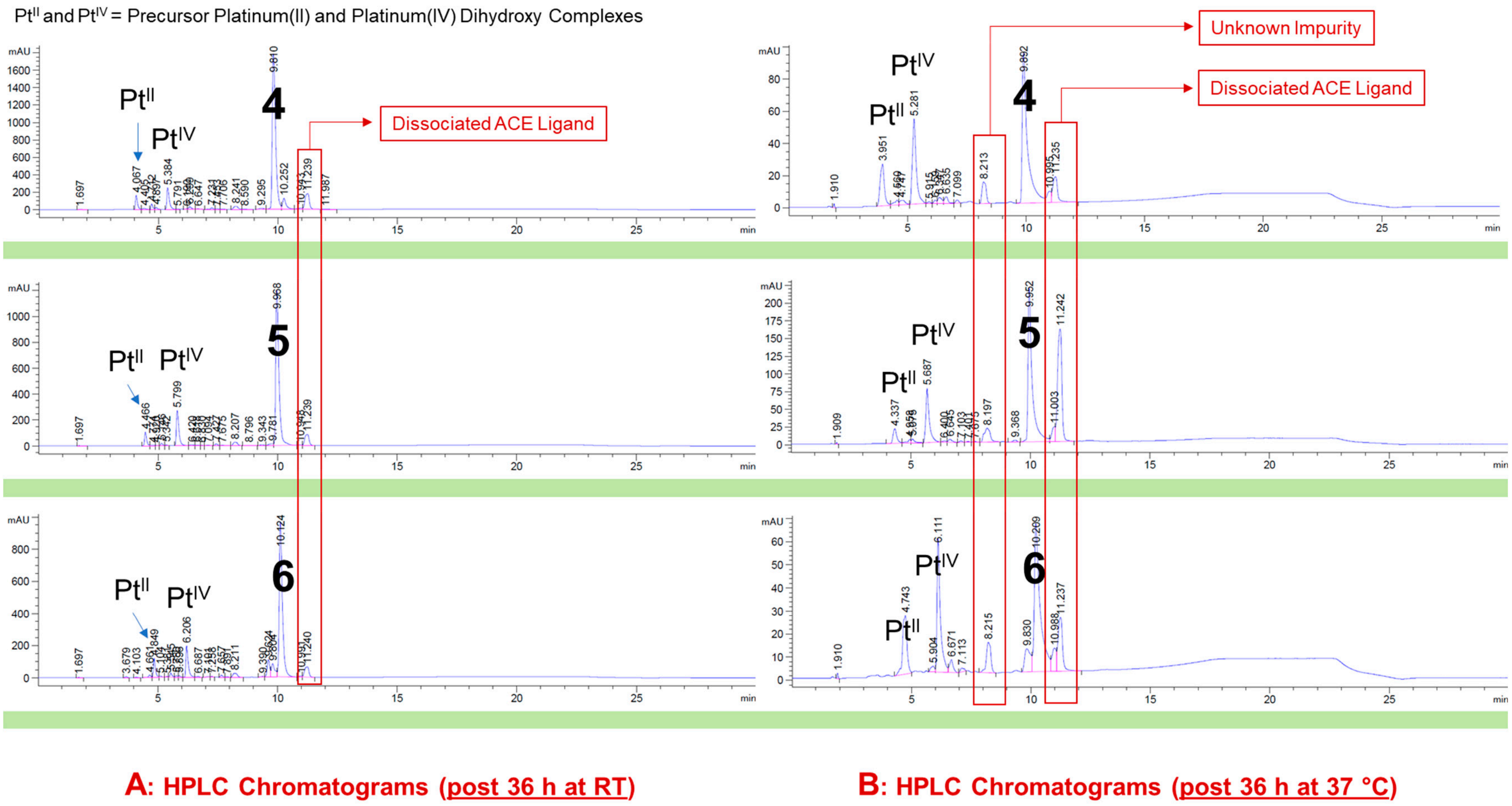

Figure S77: Combined HPLC chromatograms acquired for **4–6** incubated with PBS (~7.4 pH) at room temperature and 37 °C after 36 h.

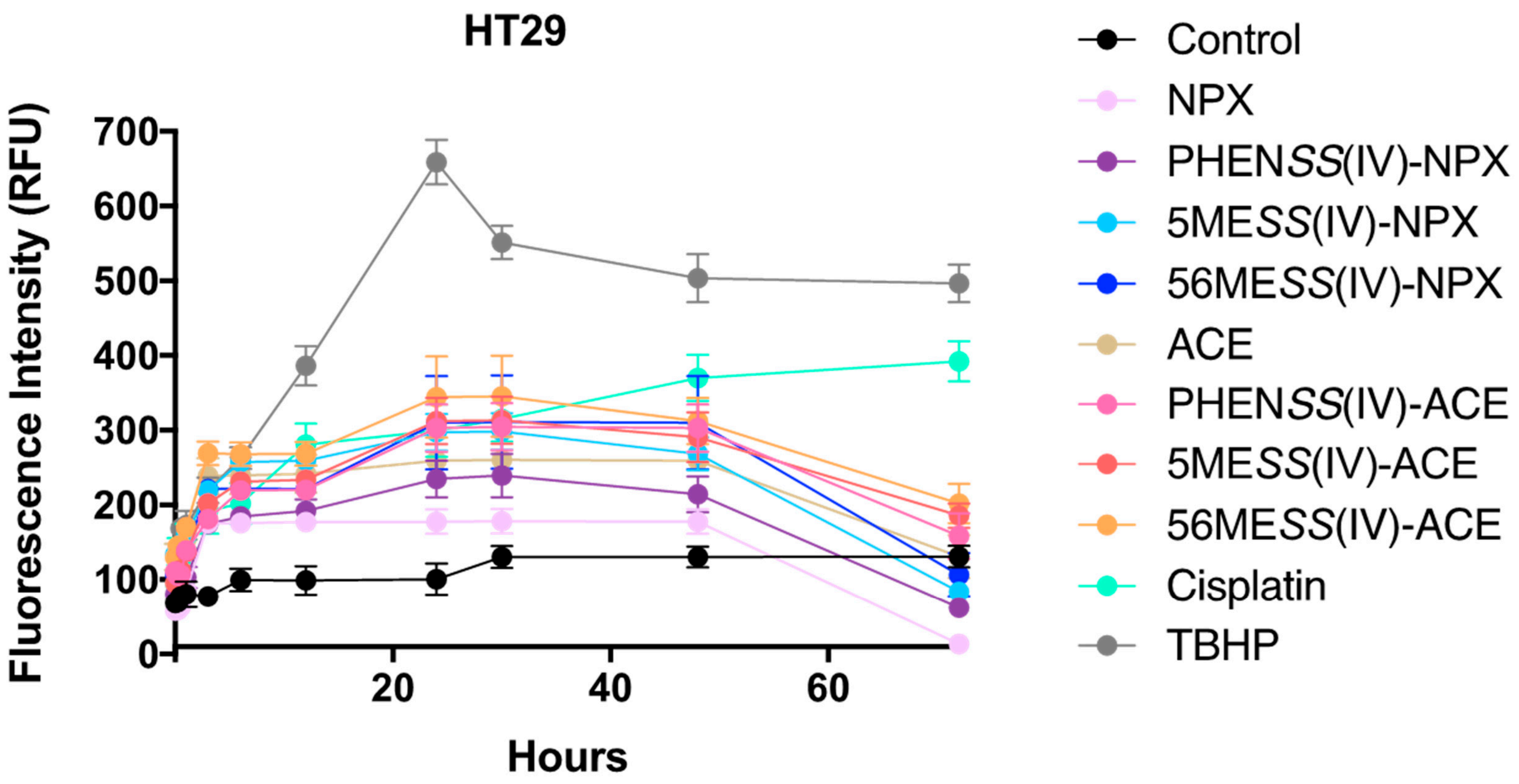

Figure S78: ROS production upon treatment with **1–6**, NPX, ACE, and cisplatin in HT29 colon cells at 0, 0.25, 0.5, 1, 3, 6, 12, 24, 48 and 72 h. **PHEN*SS*(IV)-NPX** (**1**), **5ME*SS*(IV)-NPX** (**2**), **56ME*SS*(IV)-NPX** (**3**), **PHEN*SS*(IV)-ACE** (**4**), **5ME*SS*(IV)-ACE** (**5**), and **56ME*SS*(IV)-ACE** (**6**). TBHP: t-butyl hydroperoxide. Data points denote mean ± SEM. *n* = 3 from three independent experiments where samples were run in triplicates.

COX-2 Inhibition Measurements

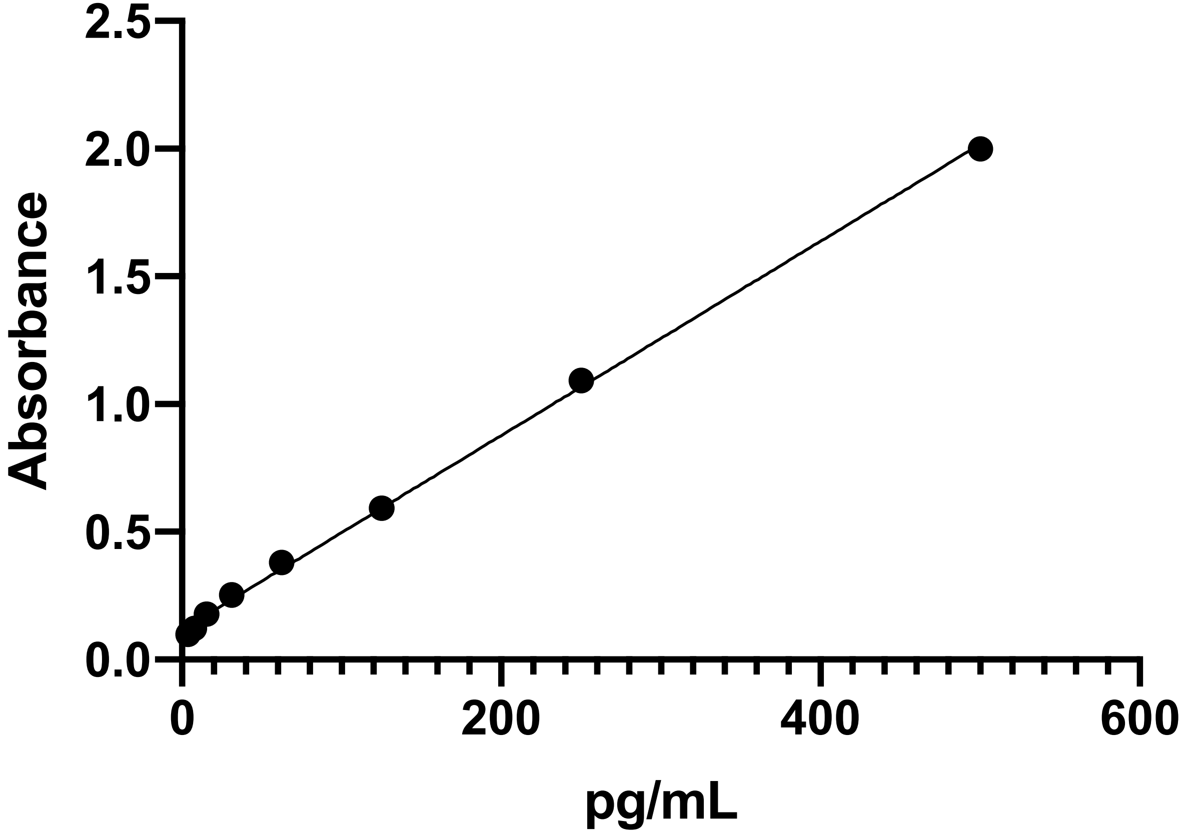

Figure S79: COX-2 calibration curve generated by plotting the absorbance against known concentrations (pg/mL). This curve was used to quantify the inhibition of COX-2 in HT29 cells after a 72 h treatment with complexes **1–6**, NPX, ACE or cisplatin; y= 0.0038x + 0.1167 and R^2= 0.9988
